# Sharing real-world data for public benefit: a qualitative exploration of stakeholder views and perceptions

**DOI:** 10.1186/s12889-023-15035-w

**Published:** 2023-01-19

**Authors:** Susan Baxter, Matthew Franklin, Annette Haywood, Tony Stone, Monica Jones, Suzanne Mason, Kamil Sterniczuk

**Affiliations:** 1 School of Health and Related Research (ScHARR), University of Sheffield, Regent Court, 30 Regent Street, Sheffield, UK; 2 Professional Services, University of Leeds, Woodhouse Lane, LS29JT Leeds, England; 3 Public advisor, Sheffield, England

**Keywords:** Data sharing, Real world data, Public health, Routine data, Information governance, Integrated care, Qualitative

## Abstract

**Background:**

There has been an increasing interest in the use of “real-world” data to inform care decision making that could lead to public health benefit. Routinely collected service and activity data associated with the administration of care services and service-users (such as electronic health records or electronic social care records), hold potential to better inform effective and responsive decision-making about health and care services provided to national and local populations. This study sought to gain an in-depth understanding regarding the potential to unlock real world data that was held in individual organisations, to better inform public health decision-making. This included sharing data between and within health service providers and local governing authorities, but also with university researchers to inform the evidence base.

**Methods:**

We used qualitative methods and carried out a series of online workshops and interviews with stakeholders (senior-level decision-makers and service leads, researchers, data analysts, those with a legal and governance role, and members of the public). We identified recurring themes in initial workshops, and explored these with participants in subsequent workshops. By this iterative process we further refined the themes identified, compared views and perceptions amongst different stakeholder groups, and developed recommendations for action.

**Results:**

Our study identified key elements of context and timing, the need for a different approach, and obstacles including governmental and legal, organisational features, and process factors which adversely affect the sharing of real world data. The findings also highlighted a need for improved communication about data for secondary uses to members of the public.

**Conclusion:**

The Covid-19 pandemic context and changes to organisational structures in the health service in England have provided opportunities to address data sharing challenges. Change at national and local level is required, within current job roles and generating new jobs roles focused on the use and sharing of real-world data. The study suggests that actions can be taken to unlock the potential of real-world data for public health benefit, and provides a series of recommendations at a national level, for organisational leaders, those in data roles and those in public engagement roles.

**Supplementary Information:**

The online version contains supplementary material available at 10.1186/s12889-023-15035-w.

## Background

Over the last two decades, there has been an increasing interest in the use of “real-world” data to inform care decision making that could lead to public benefit [1, 2]. This term refers to data which is collected in routine care, service delivery or clinical practice rather than research studies (especially outside clinical trials) [3]. Routinely collected service and activity data associated with the administration of care services and service-users (such as electronic health records or electronic social care records) hold potential to better inform effective and responsive decision-making about health and care services provided to national and local populations. Sharing of these data between agencies providing healthcare and those providing other local services has potential to underpin the better commissioning of cost-effective care programmes, leading to population health benefits. A 2019 report by The Health Foundation identified several ways that improved use of real world data could inform decision-making by health and social care providers [4]. These included: enabling evaluation of innovations and new models of care to find out if expected changes and benefits were realised; informing changes to service delivery in complex organisations and care systems; better measuring and evaluating improvements; and gaining a better understanding of how patients flow through the system. A UK government policy paper in the wake of the Covid-19 pandemic identified “the power of data” as “one of the most impactful tools at our disposal”, but in order for this to be realised, there was an urgent need to make “appropriate data sharing the norm and not the exception across health, adult social care and public health” [5].

The policy paper, however, notes the challenges in sharing data across health and other agencies, and describes “traditional divisions making it very difficult for local and national leaders across health and care to effectively plan, commission, and develop policy” [5]. In England, Local Authorities (LAs) are responsible for deciding spending priorities and providing social care and some public health services for their local population. Decision-making regarding local health spending and service provision until recently was predominantly made by Clinical Commissioning Groups (CCGs) within the National Health Service (NHS). In 2022 CCGs have been replaced by Integrated Care Systems (ICSs), with joint-working with LAs being part of the ICS’s mandate. While there are potential sizeable benefits in terms of economics, efficiency, and effectiveness from CCGs/ICSs and LAs sharing their data to inform decision-making, there are reports of frustration and limited sharing of available data, together with cultural and skills barriers [6, 7]. Mixed views from the public regarding the NHS sharing data have been described, with a report from The Kings Fund highlighting the need to balance public concerns about data usage, with enabling data to be shared and used by NHS organisations and third parties [8].

In light of the changes required and challenges reported, this study sought to gain an in-depth understanding regarding the potential to unlock real world data that was held in individual organisations to better inform local decision-making This included sharing data between and within the NHS (i.e. CCGs/ICSs) and LAs, but also with university researchers to inform studies and the evidence base. We aimed to explore the views and experiences of a range of stakeholders encompassing NHS and LA staff, university-based researchers, and the general public, regarding the availability and potential utility of sharing routinely collected real-world data, to support public health-related decision-making and improved population health.

## Methods

The study overall encompassed four linked work packages comprising: a mapping review of grey literature reporting examples of the use and linkage of routine data for public health-related decisions; development of a metadata specification and pilot metadata catalogue via stakeholder consultation; use of economic evaluation methods to analyse and present estimates from routine data to inform commissioning; and a series of workshops with stakeholders to explore why, where and how data sharing might be best enabled between health services and LAs. In this paper, we focus on reporting findings from the series of online workshops and interviews with stakeholders, which included senior-level decision-makers and service leads, researchers, data analysts, those with a legal and governance role, and members of the public.

The design and methods used are outlined below, based on the Consolidated Criteria for Reporting Qualitative Research (COREQ) checklist [8, 9].

### Research team

The series of online workshops and interviews were carried out between July 2021 and February 2022. They were led by a female Senior Research Fellow (SB) who has over 20 years of experience carrying out qualitative research. While the interviews were carried out by a single researcher, the workshops were co-facilitated by the study lead (MF), and other members of the team were often present and contributed to discussion. Participants had no prior knowledge of the session lead, but some were already known to other members of the team from communication about the study or other previous research. At the beginning of each session, brief introductions were made by all present.

### Ethical approval and consent to participate

The study received ethical approval from the University of Sheffield School of Health and Related Research Ethics Committee. As the study was classified as UK National Health Service Research, the project was also approved by the Health Research Authority. Information sheets and consent forms were emailed to potential participants prior to data collection, and before each workshop or interview the consent form was screen-shared. Each consent question was read aloud and anyone who did not consent was requested to close their browser and leave the session.

### Research design

The study used methods of online workshops and online interviews with stakeholders in different roles to collect qualitative data. Data collection drew on action research theoretical approaches to policy evaluation [10] and was planned to be cyclical and iterative in order that findings from each workshop informed discussion at subsequent sessions. See Table 1 for a summary of the series of workshops and interviews with senior staff and decision-makers, data analysts and researchers, people with legal and information governance roles, and members of the public. We had planned that the second phase would comprise a further workshop with senior staff and decision-makers, but following limited availability, we replaced the workshop with interviews to increase participation, which was endorsed by our study steering committee. Due to the Covid-19 pandemic all workshops and interviews took place online.

In the study planning and development phase we had determined that falls prevention would be a useful case study focus for the workshops, as stakeholders reported that it was an important issue crossing the boundary of health and local authority (LA) care. This topic area was used to focus discussion (particularly with group 1), although much discussion related to data sharing between LA and health services more broadly.

**Table 1:** Summary of the workshop and interview series

Phases (P#)	Group 1: Decision-makers, clinical directors, and senior clinicians	Group 2: Data analysts and researchers	Group 3: People with a legal or information governance role	Group 4: Members of the public
P#1: Initial discussion of current situation and obstacles to sharing data	Workshop #1	Workshop #2	Workshop #3	N/A
P#2: Further developing understanding of data availability, useability and sharability	Interviews	Workshop #4	Workshop #5	Workshop #6
P#3: Conclusions and recommendations – exploring potential solutions	Workshop #7 and #8 Combined-groups workshops			Workshop #9

### Participant selection

We had agreement to participate from the LA and CCG in two cities in the North of England, together with their Universities. Unfortunately, one of the CCGs withdrew part-way through the study, leaving us with a CCG, LA, and University from one city, and an LA and University from the second city. Leeds has a population of around 798,800, 87% White, and a highly diverse population in terms of level of affluence. Sheffield has a population of around 556,500, 84% White, also with areas of both high and low income, and around 35% of the population living below the poverty line. Leeds ranks fourth and Sheffield fifth in the list of cities with the highest numbers of deprived areas. Both cities have an average life expectancy for men of 79 and 83 for women.

Within these organisations we sought relevant individuals to approach, using our co-applicant contacts and knowledge of their organisations. We aimed for representatives with similar roles in each organisation, within the three groups of commissioners, directors and senior clinicians (service leads), people who analyse data, and people with legal or governance roles. We did not have a target sample size or select from those who we identified, but invited anybody with relevant experience to take part. There was an element of “snowballing”, with participants suggesting others who would have relevant expertise.

For the public workshops we deliberately sought diversity in participants, drawing on our study public co-applicant and public advisory group to draft and distribute an advertising flyer via their networks. We also advertised on the People in Research website (https://www.peopleinresearch.org), used our database of people interested in research, and used student lists to seek potential participants. We requested information on age, gender, ethnicity, area of the UK, and views of/interest in data sharing in responses, and purposively selected from those who responded by scrutinising answers to this information.

### Data collection

Workshops lasted one and a half hours and were recorded using a handheld encrypted device positioned close to the laptop speaker. The format of the phase one workshops consisted of introductions and outline of the study, followed by open discussion and use of Powerpoint slides which were edited live during the discussion. From workshop two onwards we were able to present summaries of the discussion from previous sessions to further explore and develop. This was as an important means of sense checking our understanding of what had been said, and acted as a method of respondent validation to aid the quality/trustworthiness of the research [11].

Interviews typically lasted 45 min to one hour and were recorded using a handheld encrypted data recorder. The interviews consisted of a brief introduction to the study, followed by screen sharing slides summarising workshop findings up to that point, for participants to comment on and discuss further. Interviewees were asked whether there was anything surprising or particularly interesting about what was said, and for anything which was incorrect or they would like to add. See Supplementary material File 1 for the interview topic guide.

The format of the public workshop was divided into two components. The first half of the session comprised introductions and a summary of the research, followed by presentation of brief bullet points from the staff workshop discussions. Questions and comments were invited on these points. In the second half of the session participants were divided into groups to discuss examples of data sharing initiatives which they had been sent to look at beforehand (Supplementary material File 1). Discussion was structured around four questions: do you have any concerns about use of data in this example; did anything particularly interest or surprise you about your case study; would the public fully understand what their data was being used for in this example; and do you think this is a good use of data?

### Data analysis

Data were in the form of recordings from the workshops and interviews. Given the short turn-around time between the series of workshops, interviews and further workshops, rather than being transcribed, the qualitative lead researcher (SB) listened to the recording (usually within a few days) and produced a document containing both researcher notes and verbatim participant quotes. These documents were then used to identify key themes and sub-themes to produce a coding tree. These themes were discussed with members of the team at regular project management groups, and with others at advisory group meetings. We rarely encountered disparity in views regarding coding, but where they occurred we reached agreement via discussion. As mentioned above, subsequent workshops and interviews were used for participant checking via the iterative process of data collection, then data analysis, then re-presentation of findings.

### Reporting

Verbatim quotations from participants have been presented to illustrate key findings, together with reporting of where there was consensus and diversity in views. Selection of quotations has been based on achieving representation across participants and roles. We considered all relevant quotes relating to each theme, and then selected those which offered greatest clarity from staff in different roles and different organisations. Findings from the public workshops will be presented separately from those with staff.

## Results

We carried out nine workshops and an additional five interviews. See Table 2 for a summary of attendees at each workshop and interview participants. In order to preserve confidentiality of participants we have not provided individual characteristics, only role and type of organisation. Many participants attended more than one workshop, or an interview and a workshop. In total there were 18 staff participants included in the study (five from one LA, five from a second LA, six from a CCG, and two from two Universities), and 19 members of the public. Staff participants were all White and twelve were male. Public participants were more diverse with 11 of 19 males, six aged under 30, eight people aged 30 to 60, five aged over 60, and six of the 19 public participants were of non-white ethnicity.

**Table 2:** Summary of participants

Workshop / interview	Description of attendees
Workshop 1	Three attendees, all senior staff within a CCG.
Workshop 2	Five attendees, people with data analysis role from 1 CCG, 2 LAs and 1 university.
Workshop 3	Four attendees, people with legal or information governance roles from 1 CCG and 2 LAs.
Workshop 4	Seven attendees, people with data analysis roles from 1 CCG, 2 LAs, and 1 university.
Workshop 5	Five attendees, people with legal or information governance roles from 1 CCG, 2 LAs, 1 university.
Workshop 6	11 members of the public and three public advisors.
Workshop 7	Seven attendees, individuals who had attended a prior workshop or interview.
Workshop 8	Eight attendees, individuals who had attended a prior workshop or interview.
Workshop 9	16 members of the public, together with 3 members of the study public advisory group.
Staff interviews	Two clinical service leads, one commissioner of services, two senior data analysts.

**Fig. 1:**
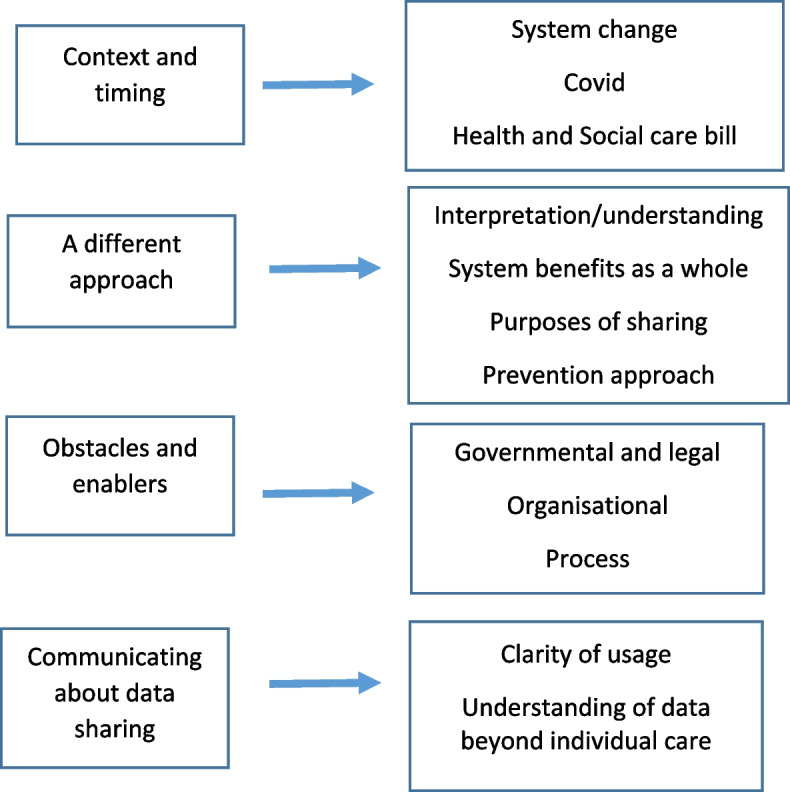
Themes and subthemes in the data

Analysis of the data from the workshops and interviews indicated four main recurring themes and corresponding sub-themes. See Fig. 1 for the coding tree. The slides with notes/responses added during the discussion are available as online supplementary material (File 2).

The first main theme brought together data on perceptions regarding the *context and timing* of changes to data sharing between organisations. Secondly, perceptions relating to the need for *a different approach* if organisations are to extend data sharing. The third theme encompasses perceived *obstacles and enablers* to data sharing between organisations, which is divided into governmental and legal; organisational; and process factors. Analysis of data from the public workshop predominantly contributed data to the fourth theme: *communicating information about data sharing to the public.*

### Context and timing

Participants described how the context of system change within and across organisations contributed to difficulties in decision-making regarding making any changes to data sharing agreements and processes. At the time of the study, considerable re-structuring was underway in the UK National Health Service as part of transitions to ICS and associated integrated care boards (ICBs) and participants emphasised the urgent need for consideration of data sharing:

“All the impending changes in ICS, ICBs etc…there has to be a change in the law on data sharing for these new structures to work….there absolutely has to be as these new structures can’t legally work under the present system” (Combined workshop).

At the time of the study, the Covid-19 pandemic was a challenging context that had skewed organisational priorities, but was reported to have acted as an enabler in showing what can and might be achieved:

“We will see if this opens things up. COPI shows that LAs and NHS can meaningfully share data to improve health” (Legal and IG workshop).

The Control of Patient Information (COPI) Notices enacted during and due to the Covid-19 pandemic had permitted organisations to share data far more freely than allowed under normal circumstances, but still within current legal and information governance frameworks:

“Increased information sharing, in Covid was a response to the extraordinary circumstances we were in. The reality is that it is being worked through and the emergency is ending. Organisations will rightly revert to mainstream approaches on the frameworks that exist” (Legal and IG workshop).

At the time of the workshops these COPI notices were due to end in their current format, and there were hopes that progress made might be sustained:

“More opportunities are presenting themselves so might not revert or land somewhere better…we have more people in organisations who understand the data sharing landscape, will be a lot of push from NHS and LA so things don’t revert back” (Data analysts workshop).

It was agreed that an important priority was finding a way to continue the data sharing and uses that had been established for public benefit:

“We need to find a legitimate legal way of doing what we have been doing over the last 18 months realistically on a day to day basis going forward” (Legal and IG workshop).

Participants perceived that the success of data sharing during the pandemic indicated greater anxiety within organisations than is necessary. It was reported that prior to the pandemic there had been the “beginnings of exploration of sharing and having shared data” (Data analysts workshop) but these had been somewhat limited in nature.

Another key contextual factor highlighted was the Health and Care Bill 2021. Although detail regarding what would be contained in the new legislation was very limited at the time of the study, it was perceived as an opportunity for change however, many participants were pessimistic as to whether it would permit new data sharing arrangements:

“The new Act going through parliament referred to changing the law to improve data flow for commissioners so I suspect that will be just for commissioners/NHS” (Interview 4).

### A different approach

Decision-makers and those who led services repeatedly emphasised that interpretation and understanding is key, and that data has to be translated into a useable form for people who are not analysts:

“The way the data relates to actual practice is the tricky bit and the bit you need to understand if you want to interpret it correctly” (Data analysts workshop).

Better understanding of the data landscape is needed, including what data might be available and what might be useful to answer key questions. This gap in understanding was within organisations, but was also particularly apparent between organisations, with each not being fully aware of what each other has:

“I can’t remember the last time I sat down with anyone from the council and said have you got anything new and exciting which you could give us” (Data analysts workshop).

It was emphasised that progress in exploring the utility of new sources was currently limited by the reality that “data returns are the driver for what data are required” (Senior staff workshop).

The need for “a different approach” was particularly apparent in discussions regarding organisations having different priorities and drivers. Progress requires better recognition of potential benefits for the system as a whole, and “seeing the bigger picture”. If system level rather than organisational-level decisions were to become a reality however, enhanced communication and co-operation are required.

Some participants in LAs bemoaned a persisting “medical model view”, and perceived that health services were not appreciating the potential value of LA data for population health decision-making:

“Part of it is a lack of understanding from the CCG on how they could use LA data, we know how we could use GP data. I have been trying to stimulate that conversation for years” (Data analysts workshop).

Participants perceived that health services and local authorities had different concepts of prevention, with a tendency in health services for a focus downstream rather than at a population level, for example:

“When you are talking to services they are talking in their paradigm…when someone has developed a problem….NHS is a treatment concept not a primary prevention concept….commissioners do not commission preventive services only treatment” (Interview 4).

Participants in legal and information governance roles emphasised that data flows from the functions of organisations, and it is essential to think strategically about the purpose(s) of data sharing in regard to organisational function. As one participant summarised:

“Any changes in legislation would have to focus on the purpose of data sharing and give new powers for bodies to share information to better enable their functions” (Legal and IG workshop).

It was highlighted that LAs have a much broader remit of services (functions) making the purpose of them having health data less clear. This creates a need for an approach where there is confidence that LAs will use health data appropriately.

### Obstacles and enablers

#### Governmental and legal

Participants described how structures at a national level such as different government departments lead to information being processed in different ways, and different advice being issued to health services versus LAs and even different areas of LA provision:

“Things are disjointed…two white papers, one on levelling up, one on disparities being run by separate departments and separate civil servants (Data analysts workshop).

There was considerable frustration voiced in interviews and workshops at the restrictions placed on sharing routine data:

“LA don’t share with health, not that they won’t share they can’t LA would love to push the button on it, it would make all our lives so much easier, but if any of us receive it we would have to report and you get fined for sharing information out of the IG clearance” (Interview 2).

“The CCG does not own NHS data so cannot give it to LAs” (Legal and IG workshop).

However, there was also the suggestion from some participants that the purpose for collecting (and then potentially sharing) data was not always clearly defined:

“What we tend to do is collect lots and lots of information….without always knowing what we are collecting it for” (Interview 1).

This highlighted that data protection balances are important:

“Legislative frameworks always have to strike the balance for individuals, to ensure safeguards for individuals” (Legal and IG workshop).

It was also noted that the COPI Notices enacted during the Covid pandemic had been driven by having a clear purpose for data sharing:

“The critical issue was we were able to make a very strong consistent case for why we needed access to the data – health protection” (Legal and IG workshop).

Participants in the legal and IG workshop highlighted that the NHS Act 2006, leading into the Health and Social Care Act 2012, distinguishes the role and function of the NHS from that of LAs. This results in health services and LAs having different legal bases. LAs may only access such data for commissioning/population health purposes which must remain separate from their role as providers of other services:

“We hit buffers regarding what the council are responsible for versus what the NHS are responsible for. Those purposes don’t align. Data we held for one purpose couldn’t be sent to the council for another purpose.” (Legal and IG workshop).

The consideration of legal requirements relating to *purpose* versus *function* of organisations became an important focus of discussion in the legal and IG workshops. Potential solutions to current challenges required addressing the legal basis:

“You could manage that in legislation with a clear legislative boundary – move from function to purpose – so if there was a legal gateway saying that health information could be shared between health organisations and social care organisations for the purpose of improving health and social care outcomes – make that subject to sets of boundaries in regulations and guidance you have the enabling provision and you can put safeguards around that. So moving it out of function and into purpose. This is me imagining brand new legislation which would be like a magic wand” (Legal and IG workshop).

### Organisational

At an organisational level, workshop participants highlighted the need for senior level buy in to set the right organisational culture and risk-appetite regarding information governance and data sharing. They spoke of the need for ownership at a senior level in order to drive projects forward:

“The buy in from senior management helps, in the initial phase is crucial because of the amount of work from staff required” (Data analysts workshop).

Limited capacity/capability to deal with and interrogate data was frequently described as a challenge, and limited availability of people with specialist IT skills to put data into interpretable forms:

“People are not trained to question the data, trained to produce data over the years….that is the part people are struggling with…what is it telling me…what do I want to do now” (Interview 3).

Also, limitations on technology and systems:

“A factor is also interoperability of IT systems and infrastructure, not just staff capabilities, but also IT capabilities” (Legal and IG workshop).

Participants described differing organisational priorities and perspectives that affected moving forwards with data sharing. LAs faced considerable financial challenges meaning their priorities were budgets and finances. LAs were described as smaller and fragmented, and “sensitive to the boundaries they are operating in, sensitive about enabling legal powers, and cautious over pushing against boundaries” (Legal and IG workshop).The NHS commissioning cycle differs from LA financial years:

“We sometimes find the NHS….quite constraining…the way they do their contracting is very prescriptive….we work on a yearly basis….we try not to but when your budget is stretched it is what you do” (Interview 1).

### Process

In the data analysts and researcher workshop, it was noted that “the data linkage and anonymisation process is not simple, and the complexity has time and resource constraints”. Participants described delays before receipt of some data following validation and cleaning:

“If you want to do anything with the data that is not related to hands on patient care the data is around 2 months behind” (Data analysts workshop).

Senior staff participants outlined inconsistency or omission in recording data between organisations/services, which made comparability and usefulness more limited:

“Liquid Logic is local authority data but there isn’t a data dictionary or national data set. It is not a criticism….it is a different world….not coded in the same way….the quality is different.” (Interview 4)

Data analysts reported that individual organisations often had their own local configurations of data systems for internal purposes, as available software could be modifiable/customisable and therefore harder to share:

“It might be the same database as the same company build it, but the interpretation of data might be different because of the way we set things up in system terms” (Data analysts workshop).

Also, the intellectual property held by a software provider can limit what is distributable:

“There are restrictions how you can distribute the information, LAs are a client of the company so they have to be careful what they distribute” (Data analysts workshop).

Participants emphasised how linking data requires being able to identify the same person in different sets of data. This is challenging if data are fully anonymised:

“We don’t need names but we need personal characteristics, we have to be able to distinguish – you have got to know age, ethnicity, gender or you can’t do this properly” (Legal and IG workshop).

A key point of discussion in the later workshops was around definition of what is classed as anonymisation versus pseudonymisation, also definitions of personal data, de-personalised data, and patient information. There was consensus amongst participants that these terms were currently ill-defined, and in particular the concept of pseudonymisation needed further clarity:

“Unless we use pseudonymised data in the correct way we are going to carry on with siloed working. The technology is there why can’t we use it – surely it can’t be that difficult to get a clear definition of anonymised and pseudo-anonymised data” (Legal and IG workshop).

While all participants experienced a lack of data sharing between organisations, it was suggested that “the process does already exist” for requesting data to be shared. However, it was recognised that existing processes can be lengthy and frustrating:

“We each have a gateway for a specific purpose, what you need it for today – if you want to stray off that course, not quite in the scope for what you have access for you have to go all the way back through the process – that is the frustrating bit” (Legal and IG workshop).

CCGs, as commissioners of health services, have a precedent-set pathway for access to de-personalised patient-level healthcare data. The process was perceived to be less clear for LAs, with more effort required to gain approval for healthcare data:

“There are solutions but they are long-winded and you have to specify specific purposes to sharing the data. Because of the many functionalities in the council that could be a problem” (Legal and IG workshop).

### Communicating about data sharing to the public

At the staff workshops, there was reference to public concerns regarding the sharing of health data:

“Have had objections from patients [named area] has highest proportion opting out but these people are in the highest area of need” (Data analysts workshop).

However, it was also recognised that the public did perceive potential benefits:

“There is an appetite from patients for this. They want organisations to talk to one another but don’t want them to sell data commercially” (Data analysts workshop).

At the first public workshop we explored knowledge of and views regarding the sharing of data between health services and LAs. Participants were aware of contextual factors such as changes in healthcare structures (such as integrated care systems) and voiced uncertainty whether this “would change everything”. Following presentation of findings from the staff workshops, members of the public commented particularly on how data might be used in regard to particular population subgroups:

“How is this data going to help who are ethnic minorities or is it we don’t understand their lifestyle so we are not going to do anything…how is this data going to help ethnic minorities”.

There was uncertainty regarding the quality of the data that was being used, with questions regarding accuracy and completeness:

“One thing concerns me is the accuracy of the data that is being used to make major decisions”.

There was divergence in views regarding the safety of data:

“More and more people are saying no to sharing data because you hear about data breaches all the time”.

“There is protection, if the numbers in a particular group get too small it is dropped. My experience of using data is that it is very strong. Groups undergo very heavy checking from NHS departments to keep the data safe”.

It was apparent that most participants struggled to move beyond concepts of medical notes/individual health records being shared for the purpose of improving individual care, to the concept of larger sets of data being used for wider decision-making purposes. Those present however, indicated a desire to know more about the data, in particular its purpose, how it is shared and used, in order to fully understand and be able to give an opinion on its use:

“The data would be helpful if the benefits are highlighted better, the benefits of what they are doing need to be pinpointed”.

“The aims might be good but the public need to know how they got to deciding these aims”.

## Discussion

Analysis of data from a series of workshops and interviews with senior staff and decision-makers, data analysts, people with a legal or information governance role, and members of the public provides an understanding of views and experiences regarding sharing of data between health services and local authorities in two localities in Northern England. Analysis of data indicates key themes relating to the context for change, taking a different approach, obstacles and enablers in the form of governmental and legal, organisational, and process factors, and communicating about data sharing to the public.

There was a perception amongst participants that change could and should take place. As one participant put it “having you all round the table together it seems like there are a lot more possible solutions than thought at large”. It was interesting how little discord in views was apparent, both when the groups met separately and when they came together. Perhaps the frustrations experienced, and recognition of the limitations of the current situation, provided a common goal regarding the need for change. The data indicate that changes need to be actioned at a number of different levels, both internal and external to organisations. The final workshop brought all the groups together to discuss options for making progress, and we discussed and itemised a list of actions for different actors. Table 3 provides a summary of the actions identified for different stakeholders and levels developed from this final workshop.

**Table 3:** Summary of priority areas for action at different levels derived from the final workshops

Level of action	Actions
**National level**	Provide/clarify legislation/legal gateways to enable data sharing
	Clarifying what is meant by pseudonymisation, and the associated legal underpinnings
	Address issues of funding and resourcing for data analysis Adopt greater consistency within government departments regarding policy and messaging
	Ensure compatibility in terms of drivers for data sharing between health and non-health care services
**Organisational leaders**	Ensure senior-level and system leadership for data sharing Identify a dedicated staff role for data sharing and ensure there is transparency around who is in this role (e.g. transparent job title)
	Leadership should avoid a default “no” to data sharing resulting from perceived risk, and consider opportunities and benefits rather than focus on risks
	Organisations should take a more proactive approach, exploring local solutions, putting systems and local efforts in place to enable data sharing, and prepare for change happening Organisational cultures need to better embrace a collective approach of working for the greater good Ensure that organisations are not losing sight of the bigger picture, focus on clarifying the purpose of data sharing geared towards improvements for the end user
	Organisational leaders should be enabled to perceive sharing as necessary for the benefit of the local population
	Build on business intelligence elements in the new integrated care systems as an opportunity to break down barriers Investigate the capacity for data analytics, level of work required and resource implications for system improvements
**Those in data roles**	Explore locally how could or should the data machine work; for example development of integrated data architecture, data views, a system of systems approach, interoperability, or draw on a trusted research environments approach
	Work together to ensure consistency across organisations
	Streamline the process of making data understandable
	Create links locally to know who has what information available, how and when to use it, who can use it best, and whose responsibility is it to ensure approvals Develop a forum to have regular conversations with colleagues in other organisations
	Develop minimum data standards at an organisational level
	Data owners should ensure they have clarity regarding what they are able to share – understanding of the legal/IG basis
	Clarify the purpose of obtaining data, while being realistic and focussing on what data is needed for a specific purpose
	Work with other organisations to agree objectives and work on shared priorities
	Understand and work with what is already available, ensuring potential is being realised
	Understand how data sets flow in new environments (e.g. ICS, changing geographies)
	Develop a central place for users of data to access which is user friendly
**Those in public engagement roles**	Ensure that public buy in is fully considered in any changes to data sharing arrangements
	Draw on organisation communication teams to promote understanding amongst the general public

The four themes identified through this qualitative study reflects current research evidence which generally suggests that real-world data can be used for public benefit; however, there are concerns with its use, both from staff and public perspectives, which is limiting the potential to use this data and achieve optimum levels of public benefit. Although there are organisations external to government mandated bodies which are attempting to improve knowledge and access to real-world data produced by the current care system, such as Health Data Research UK (HDR UK), change needs to occur within the current care systems. This change is required at both national and local levels, and stemming from existing or new staff roles (e.g. data engineering, analytical, legal and IG roles).

Recent UK Governments have enacted legislation and published numerous research, guidance and policy documents with the common theme of increasing availability, use and re-use of real world data collected from across the public sector [1–18]. They set out how the Government intends to address a number of the priority areas for action identified in this study. Most of these documents propose the creation of centralised data platforms and funding has already been identified in some cases [18–20]. However these policy documents are less clear on where *local* government – the ultimate source of much of this data – fit within this data strategy; specifically how they are expected to routinely contribute to these centralised data platforms the data they collect with increasing variety, volume and velocity. In order to realise the full benefits of greater public sector data sharing there is a need to address the acknowledged variation in local organisations’ maturity around data management, data quality and IT systems and infrastructure [15].

Health Data Research UK (HDR UK) have developed a Data Utility Framework to support effective health data curation [21] it is a user-centred designed framework for objectively evaluating the likely utility of specific healthcare datasets. This is of value both for potential users of health data, and for data custodians to identify the areas to provide the optimal value for data curation investment.

### Study limitations

This study was based on data collected in a single region of England, so this needs to be considered when judging applicability and generalisability of the findings. However, we are not aware of any particular features of our participating organisations that would make them unique from other NHS or LAs organisations in England. The data were collected during the Covid-19 pandemic at a time of considerable pressure on services, so this may have limited participation from staff. Beyond achieving participation from staff in different roles within different organisations we are not able to make any claims regarding representativeness of our sample. Given the brief timescale for the study and timetabled programme of workshops we did not follow up with participants who had been unable to attend a session. One organisation (a CCG) withdrew during the study, which limited involvement to a single CCG and two LAs; however, despite the limited sample, we believe that the results offer learning which is of value to policymakers, organisations and services. While we recognise the limitation of having a single researcher carrying out the initial coding, we believe that the ongoing process of participant checking and team discussion aided consistency in analysis of the data, and clarity of the key themes.

## Conclusion

Our study identified key elements of context and timing, the need for a different approach, and obstacles including governmental and legal, organisational features, and process factors influencing the sharing of real world data. We described the challenges inherent in, and need for improved communication about data for secondary uses to members of the public. Opportunities for unlocking data following the Covid-19 pandemic were identified, but there was pessimism that change would continue/happen. By enacting change at the national and local level, within current job roles and generating new jobs roles focused on the use and sharing of real-world data, the study suggests that actions can be taken to unlock the potential of real-world data for public health benefit.

## Supplementary Information

**Additional file 1.**

**Additional file 2.**

## Data Availability

The datasets used and/or analysed during the current study are available from the corresponding author on reasonable request.

## Abbreviations

CCG: Clinical Commissioning group
COPI: Control of Patient Information
ICSs: Integrated Care Systems
IG: Information governance
NHS: National Health Service
LAs: Local Authorities
