# Supplementary material for: Sharing real-world data for public benefit: a qualitative exploration of stakeholder views and perceptions
Source: BMC Public Health. 2023 Jan 19;23:133. doi: 10.1186/s12889-023-15035-w (PMC9849106; doi:10.1186/s12889-023-15035-w)
Supplement: Supplementary file 1 — Additional file 1. [file 12889_2023_15035_MOESM1_ESM.docx]

**Unlocking Data online interview outline**

1. Participants who have not previously attended a workshop should be sent the participant information sheet and consent form in advance.
2. Screen share the consent form and confirm consent and recording.
3. Introduce the project to those who have not previously been involved.
4. Confirm the cyclical nature of the workshops and interviews.
5. Screenshare slide with main findings from workshop 1

Question – Is there anything which is surprising or interesting about what was said at this workshop?

Question – Is there anything which you would like to add to the discussion, or aspects which did not come up in discussion?

1. Screenshare slide with main findings from workshop 2

Question – Is there anything which is surprising or interesting about what was said at this workshop?

Question – Is there anything which you would like to add to the discussion, or aspects which did not come up in discussion?

1. Screenshare slide with main findings from workshop 3

Question – Is there anything which is surprising or interesting about what was said at this workshop?

Question – Is there anything which you would like to add to the discussion, or aspects which did not come up in discussion?

Thanks and end
