# Supplementary material for: Sharing real-world data for public benefit: a qualitative exploration of stakeholder views and perceptions
Source: BMC Public Health. 2023 Jan 19;23:133. doi: 10.1186/s12889-023-15035-w (PMC9849106; doi:10.1186/s12889-023-15035-w)
Supplement: Supplementary file 2 — Additional file 2. [file 12889_2023_15035_MOESM2_ESM.pptx]

## Slide 1
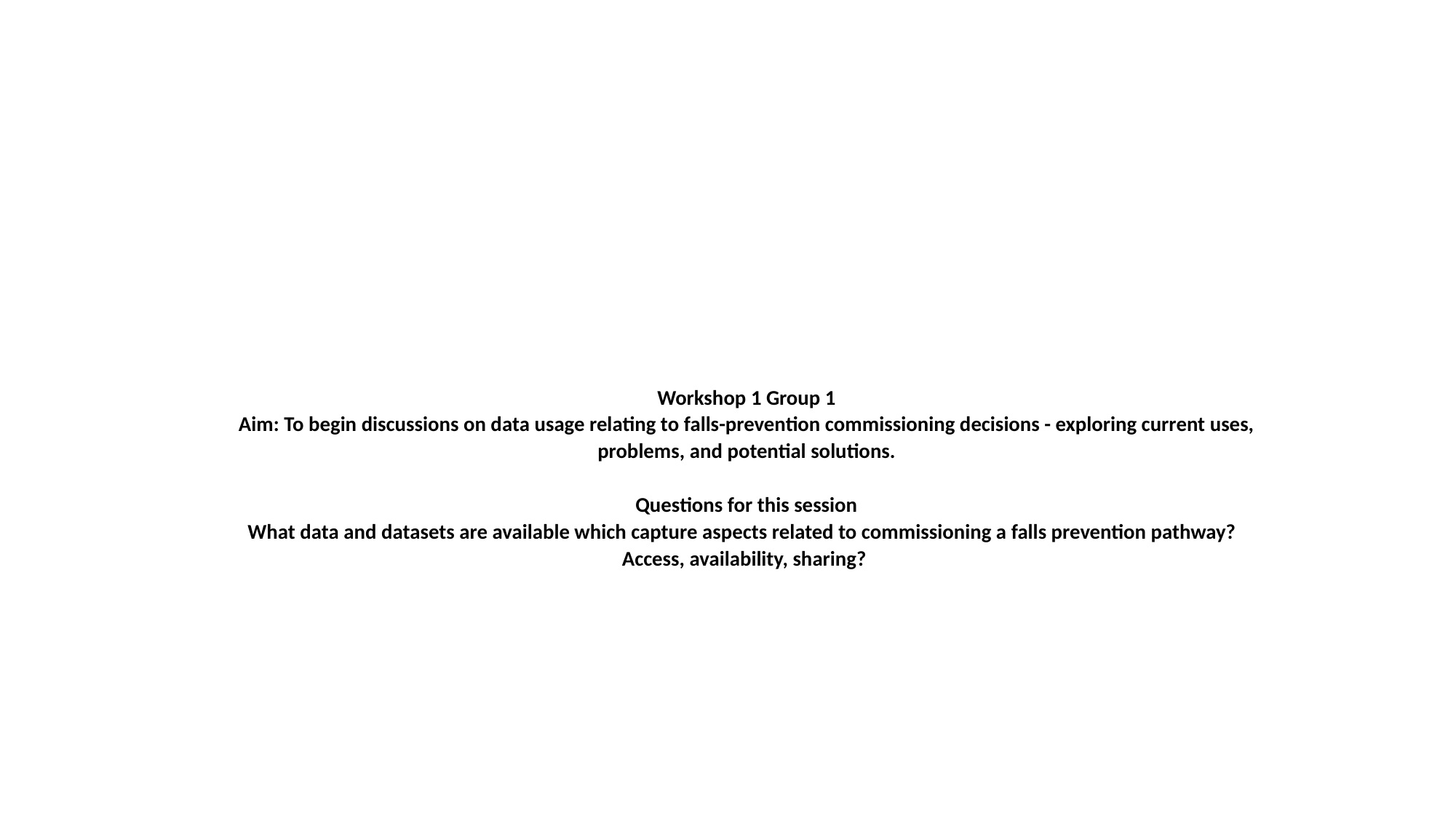

# Workshop 1 Group 1 Aim: To begin discussions on data usage relating to falls-prevention commissioning decisions - exploring current uses, problems, and potential solutions.   Questions for this session What data and datasets are available which capture aspects related to commissioning a falls prevention pathway? Access, availability, sharing?

## Slide 2
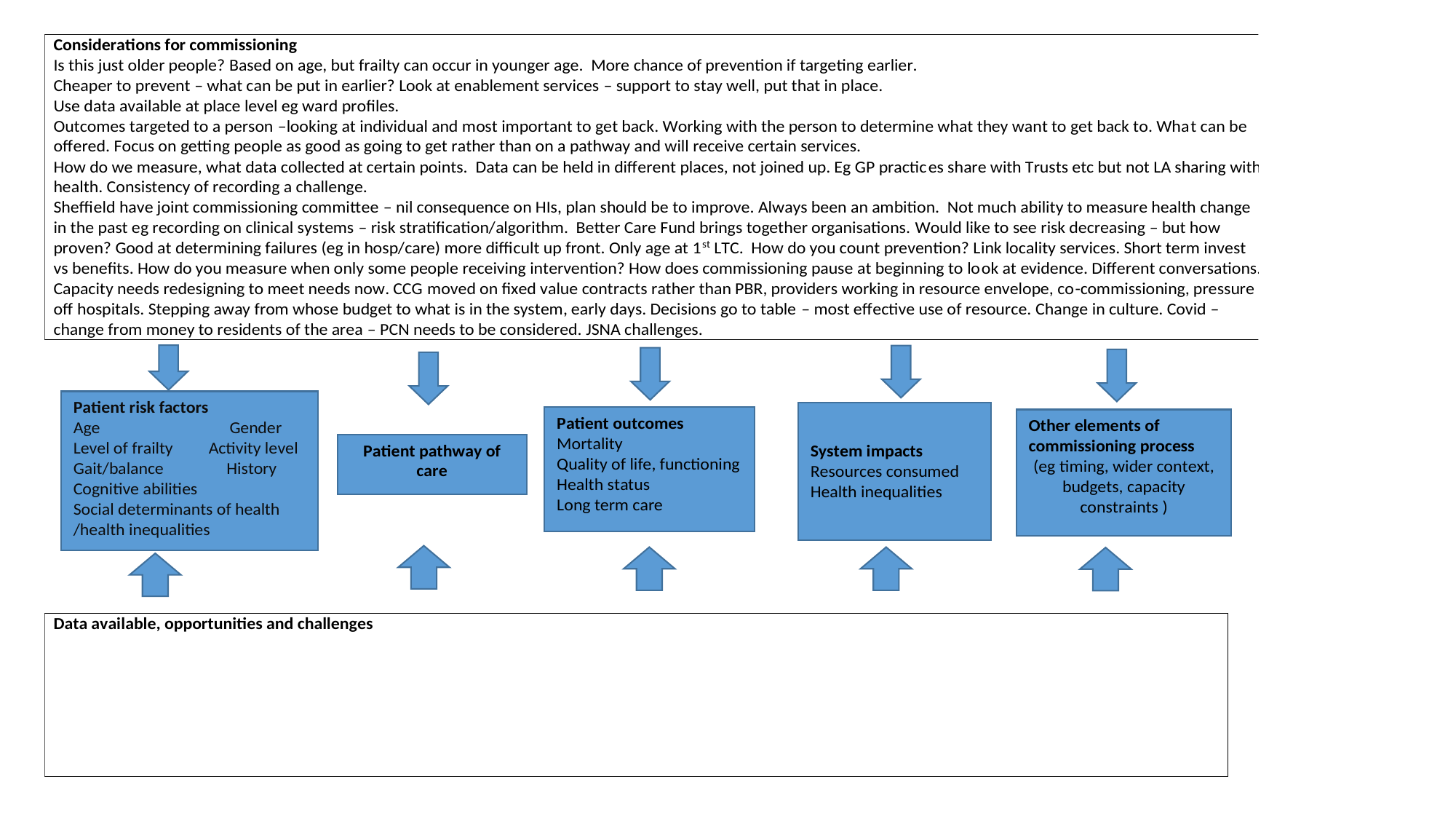

## Slide 3
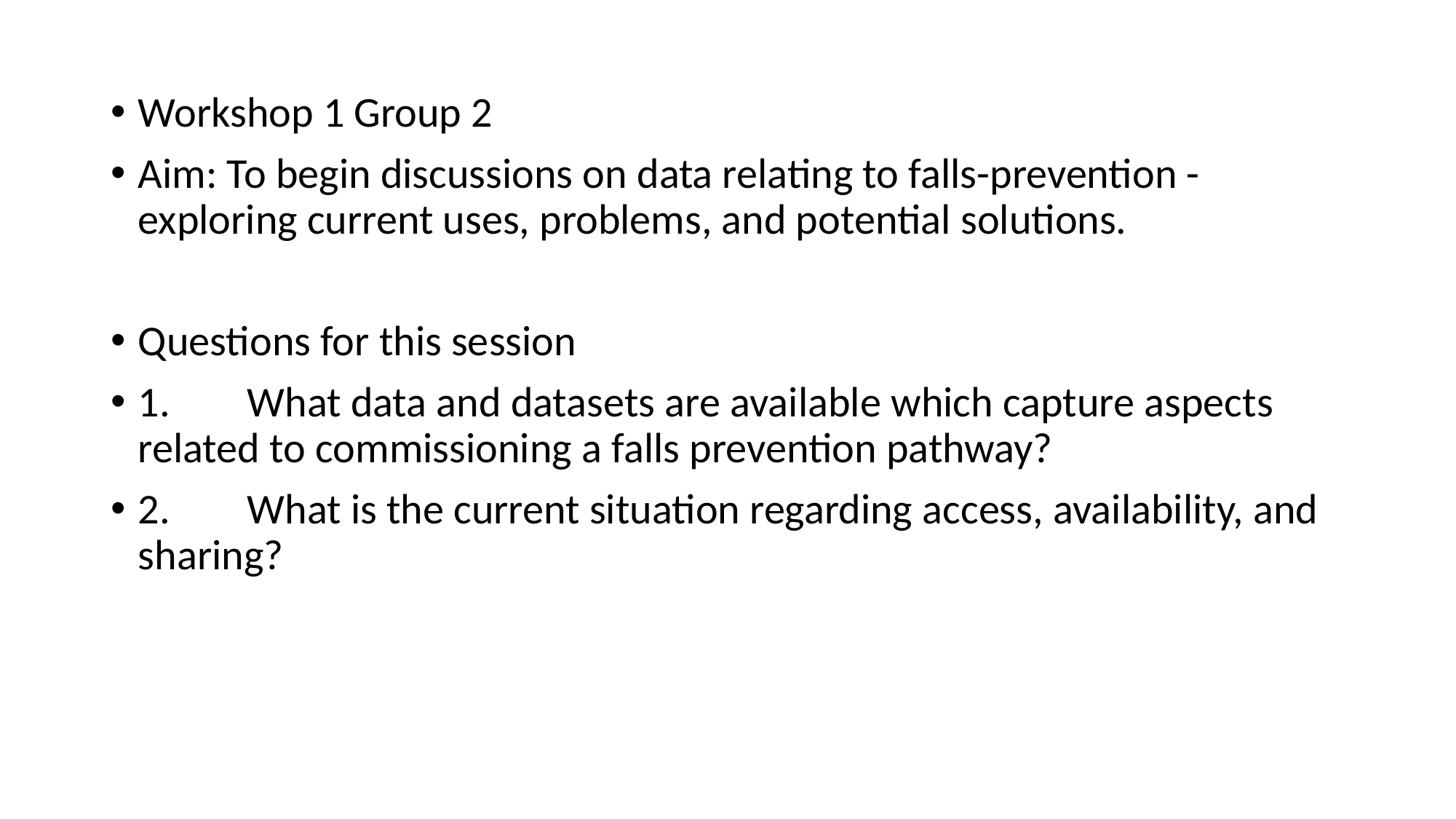

Workshop 1 Group 2
Aim: To begin discussions on data relating to falls-prevention - exploring current uses, problems, and potential solutions.
Questions for this session
1.	What data and datasets are available which capture aspects related to commissioning a falls prevention pathway?
2.	What is the current situation regarding access, availability, and sharing?

## Slide 4
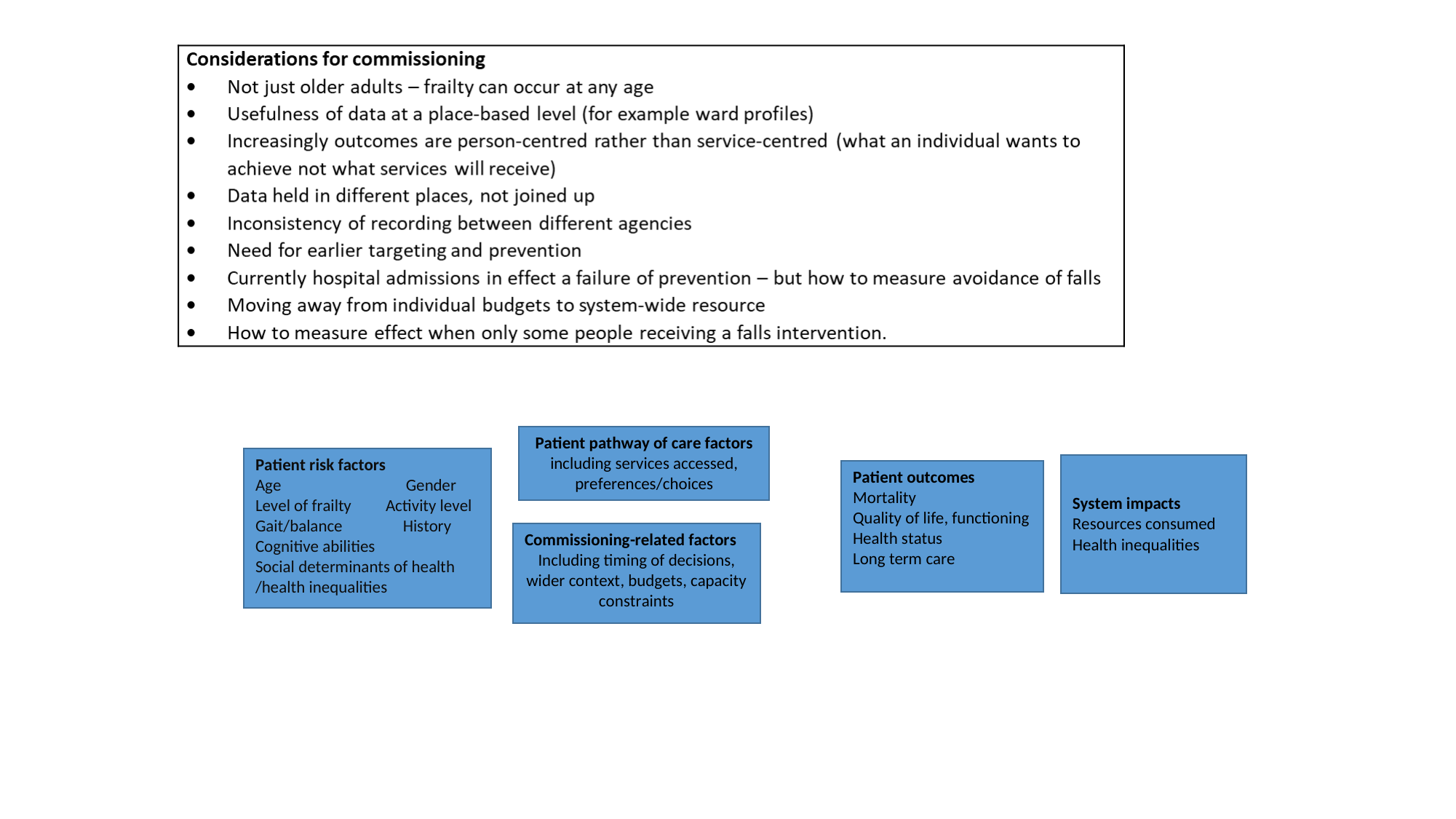

## Slide 5
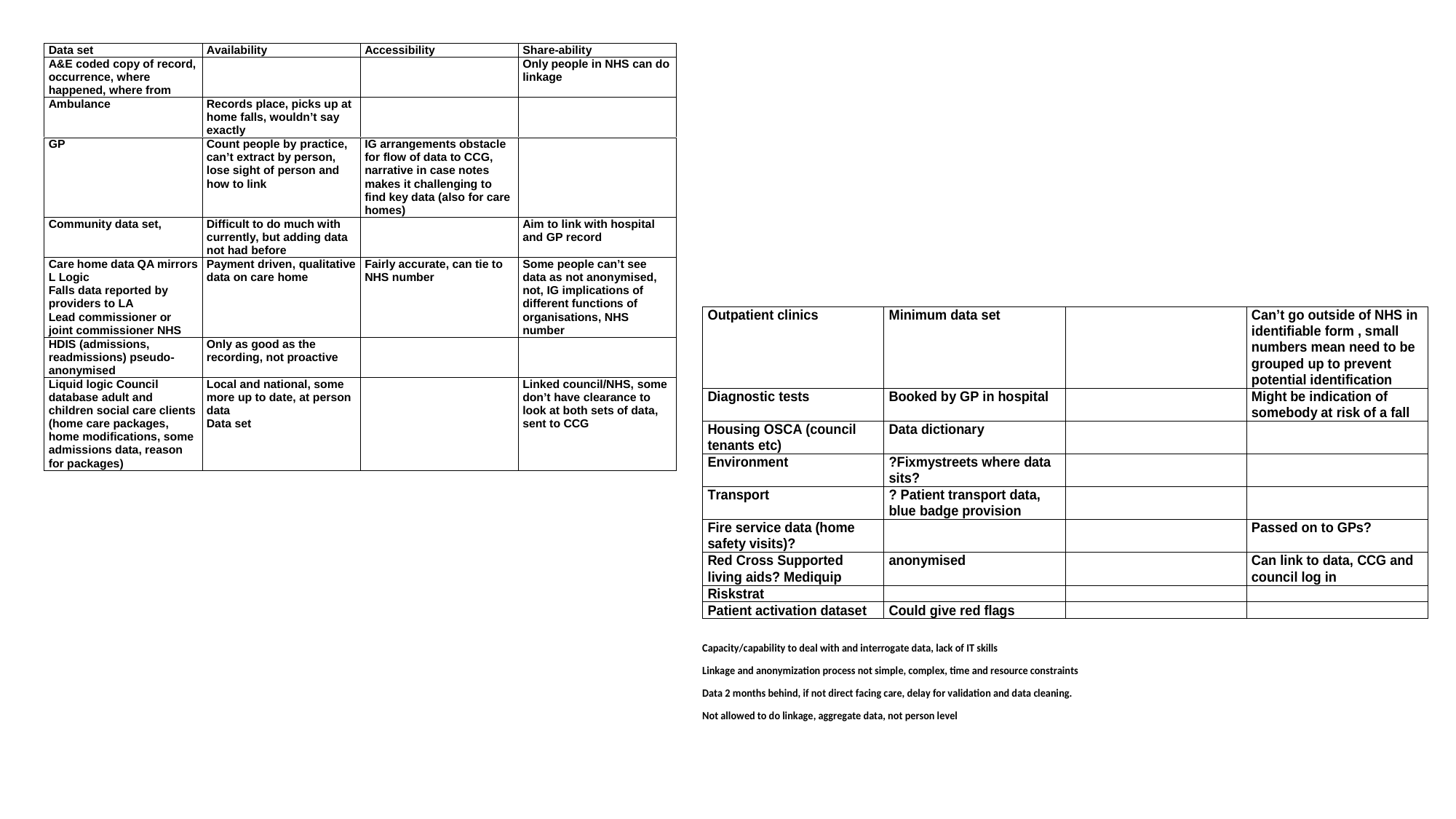

## Slide 6
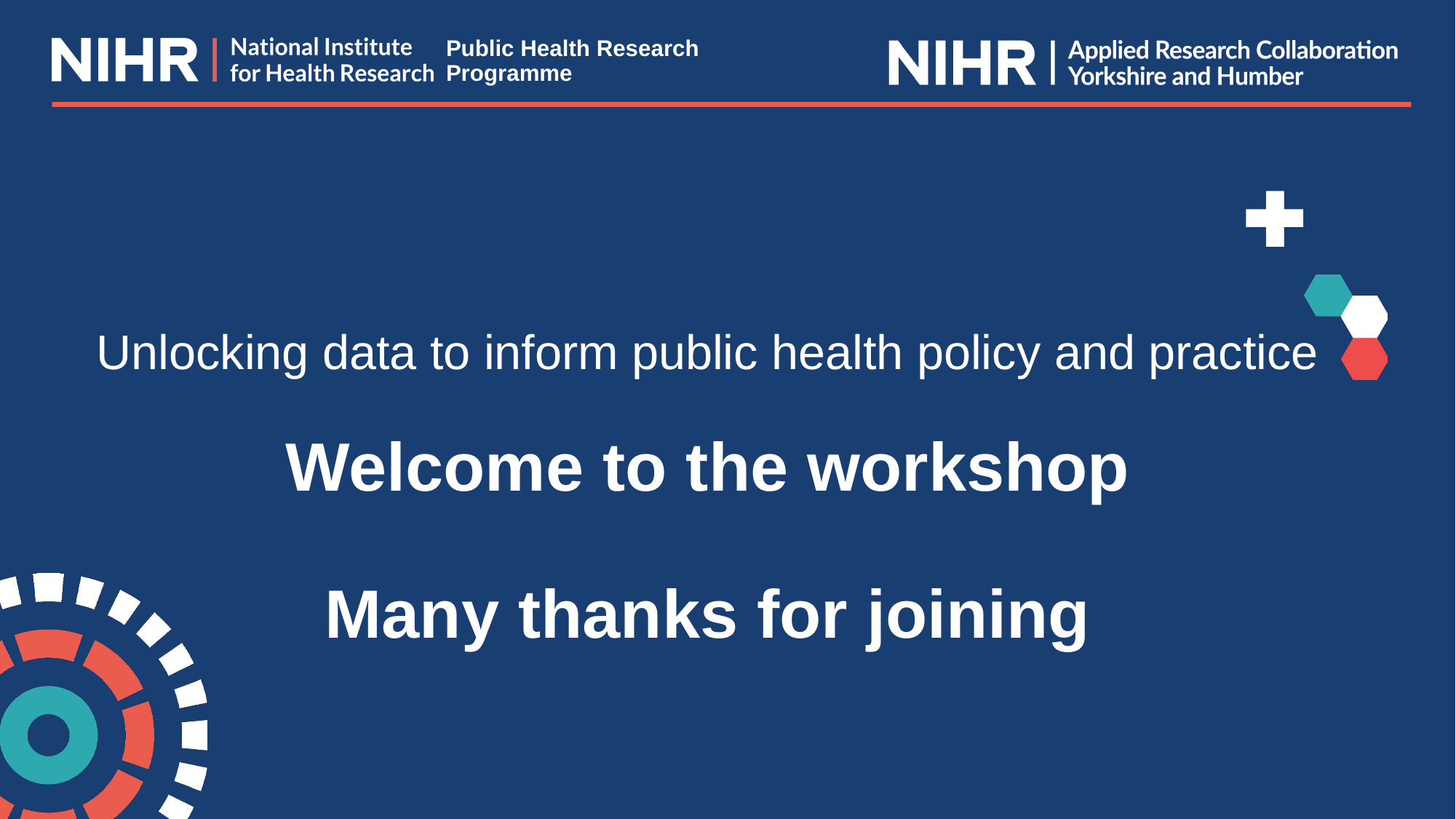

Public Health Research
Programme
# Unlocking data to inform public health policy and practice Welcome to the workshop Many thanks for joining

## Slide 7
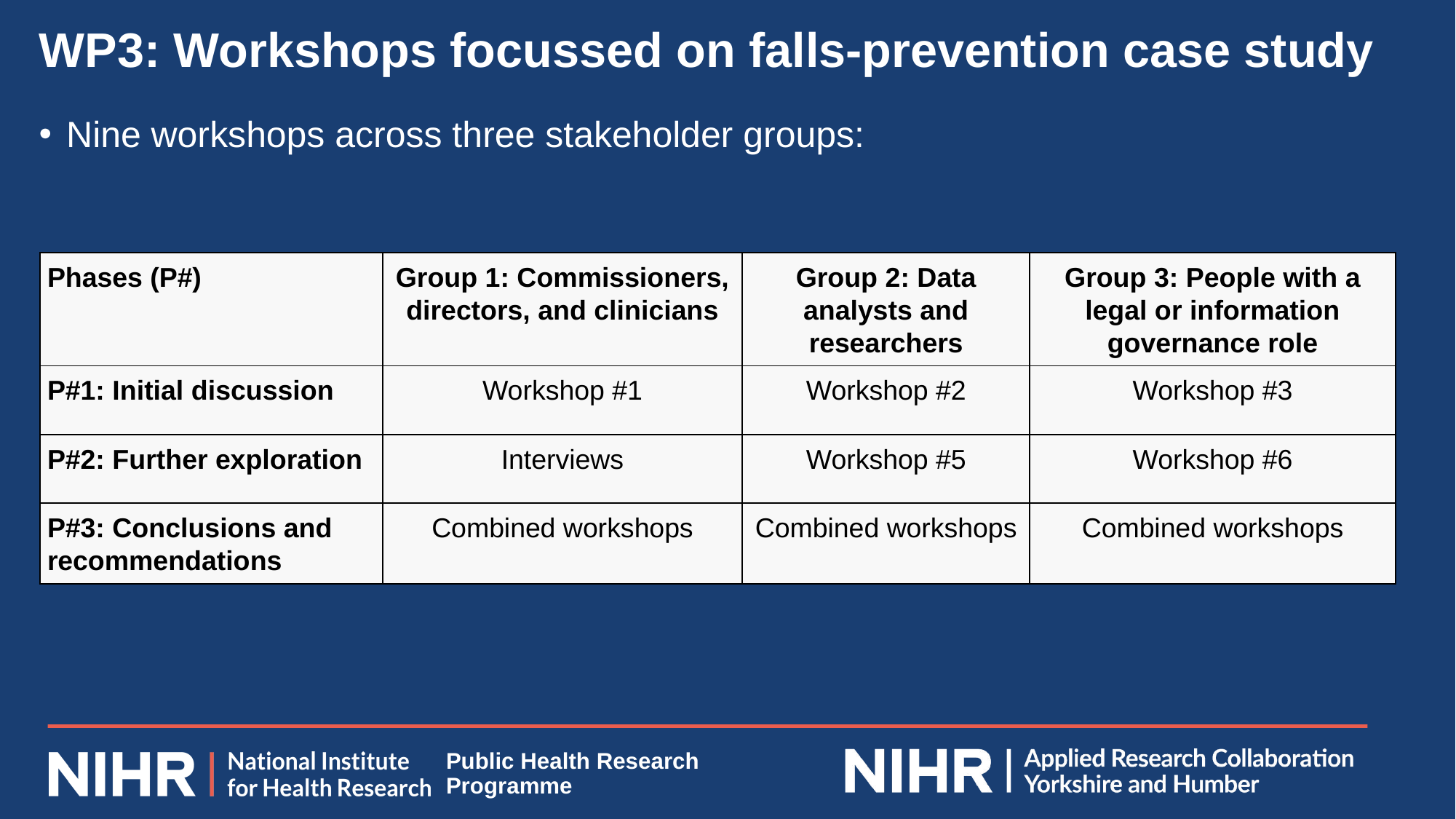

# WP3: Workshops focussed on falls-prevention case study
Nine workshops across three stakeholder groups:
| Phases (P#) | Group 1: Commissioners, directors, and clinicians | Group 2: Data analysts and researchers | Group 3: People with a legal or information governance role |
| --- | --- | --- | --- |
| P#1: Initial discussion | Workshop #1 | Workshop #2 | Workshop #3 |
| P#2: Further exploration | Interviews | Workshop #5 | Workshop #6 |
| P#3: Conclusions and recommendations | Combined workshops | Combined workshops | Combined workshops |
Public Health Research
Programme

## Slide 8
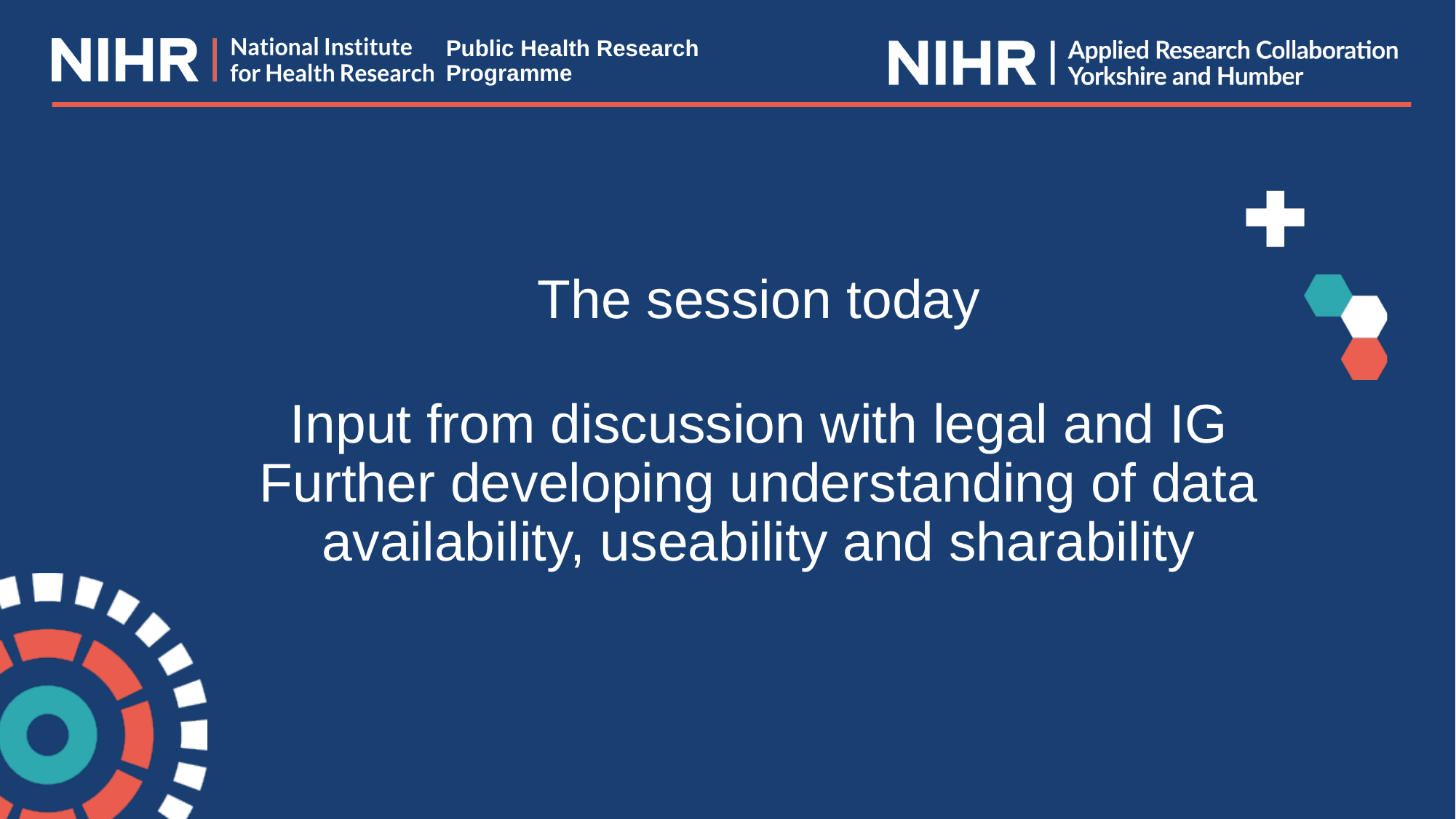

Public Health Research
Programme
# The session today
Input from discussion with legal and IG
Further developing understanding of data availability, useability and sharability

## Slide 9
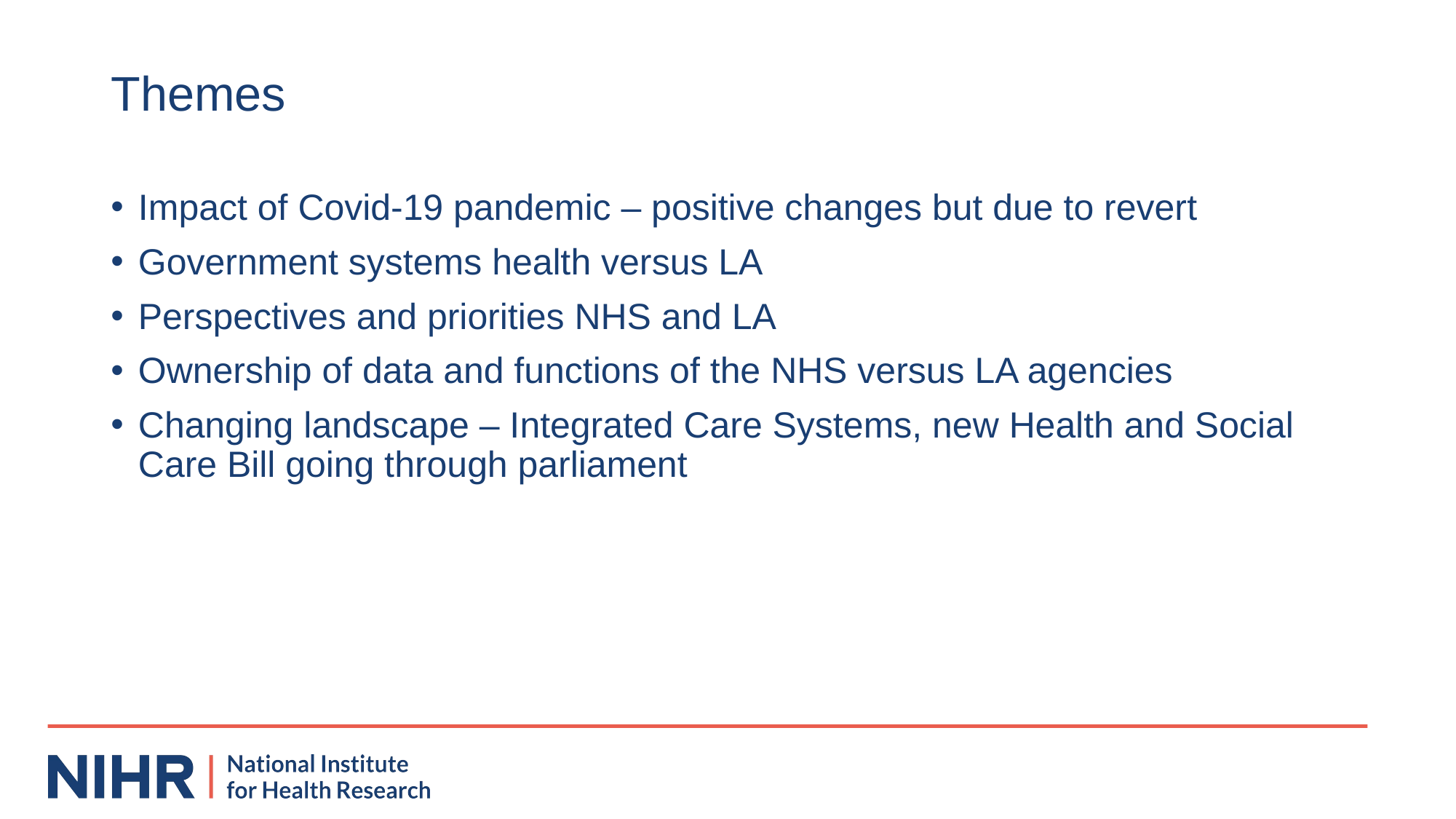

# Themes
Impact of Covid-19 pandemic – positive changes but due to revert
Government systems health versus LA
Perspectives and priorities NHS and LA
Ownership of data and functions of the NHS versus LA agencies
Changing landscape – Integrated Care Systems, new Health and Social Care Bill going through parliament

## Slide 10
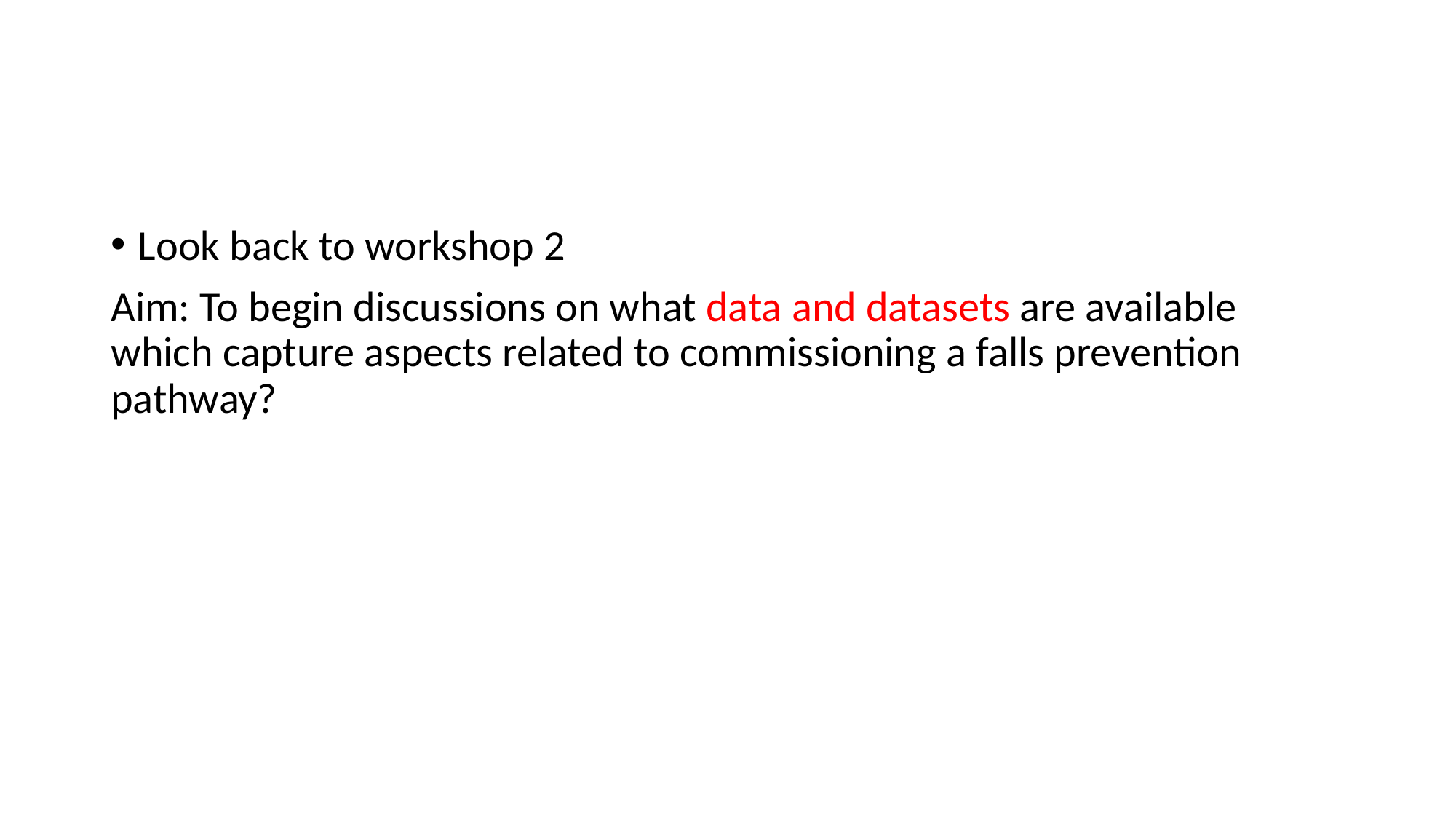

Look back to workshop 2
Aim: To begin discussions on what data and datasets are available which capture aspects related to commissioning a falls prevention pathway?

## Slide 11
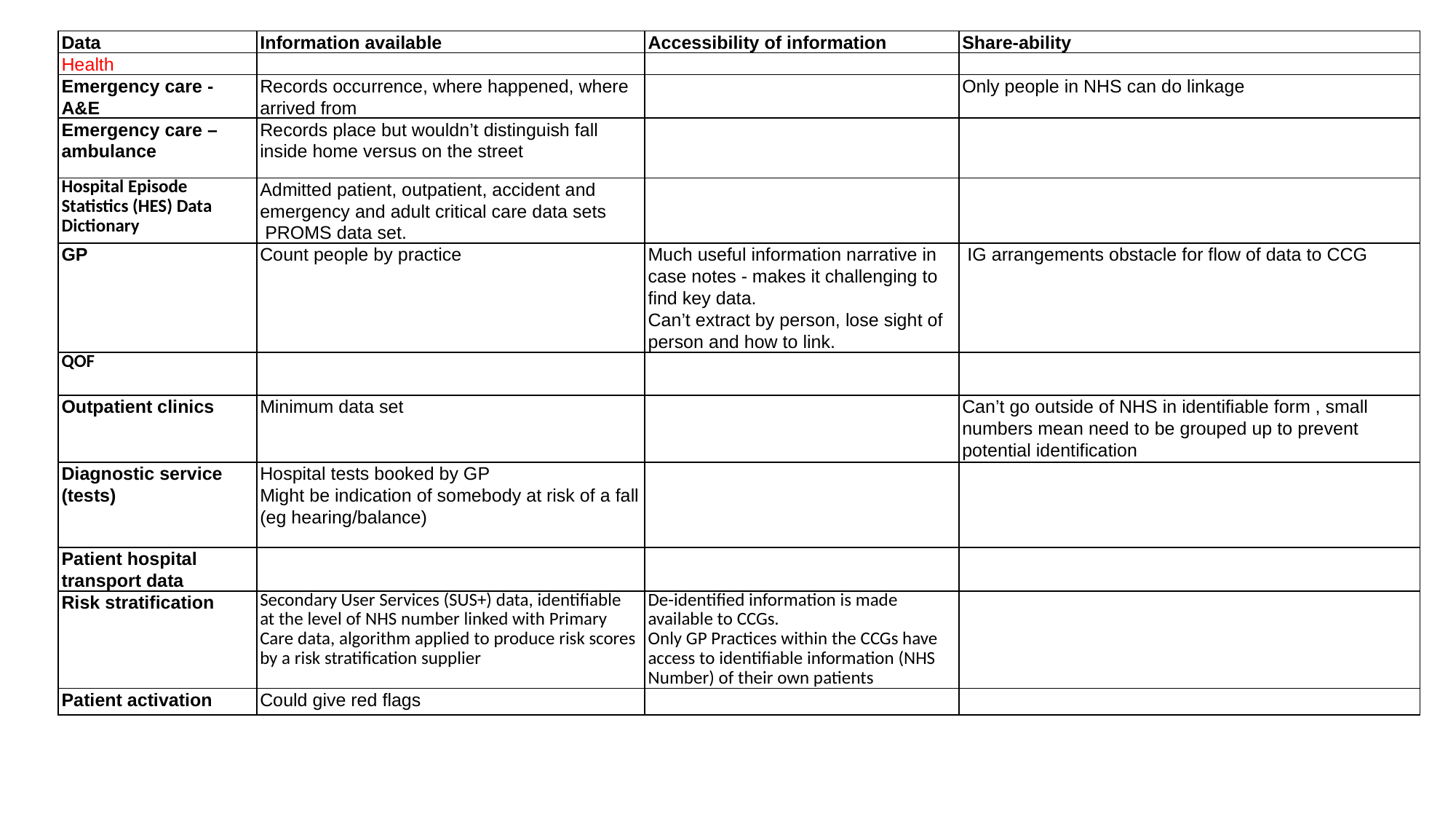

| Data | Information available | Accessibility of information | Share-ability |
| --- | --- | --- | --- |
| Health | | | |
| Emergency care - A&E | Records occurrence, where happened, where arrived from | | Only people in NHS can do linkage |
| Emergency care – ambulance | Records place but wouldn’t distinguish fall inside home versus on the street | | |
| Hospital Episode Statistics (HES) Data Dictionary | Admitted patient, outpatient, accident and emergency and adult critical care data sets  PROMS data set. | | |
| GP | Count people by practice | Much useful information narrative in case notes - makes it challenging to find key data. Can’t extract by person, lose sight of person and how to link. | IG arrangements obstacle for flow of data to CCG |
| QOF | | | |
| Outpatient clinics | Minimum data set | | Can’t go outside of NHS in identifiable form , small numbers mean need to be grouped up to prevent potential identification |
| Diagnostic service (tests) | Hospital tests booked by GP Might be indication of somebody at risk of a fall (eg hearing/balance) | | |
| Patient hospital transport data | | | |
| Risk stratification | Secondary User Services (SUS+) data, identifiable at the level of NHS number linked with Primary Care data, algorithm applied to produce risk scores by a risk stratification supplier | De-identified information is made available to CCGs. Only GP Practices within the CCGs have access to identifiable information (NHS Number) of their own patients | |
| Patient activation | Could give red flags | | |

## Slide 12
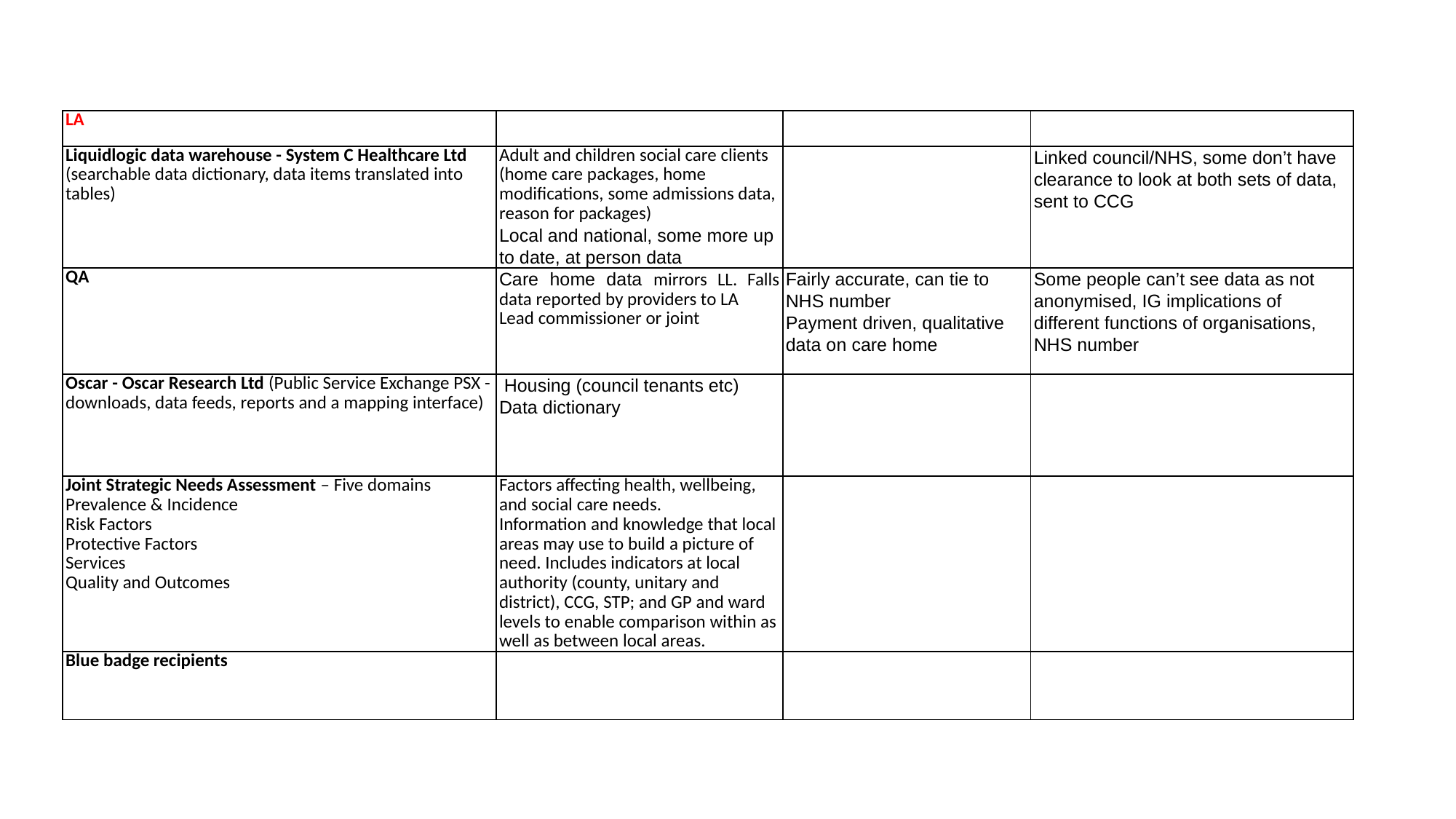

| LA | | | |
| --- | --- | --- | --- |
| Liquidlogic data warehouse - System C Healthcare Ltd (searchable data dictionary, data items translated into tables) | Adult and children social care clients (home care packages, home modifications, some admissions data, reason for packages) Local and national, some more up to date, at person data | | Linked council/NHS, some don’t have clearance to look at both sets of data, sent to CCG |
| QA | Care home data mirrors LL. Falls data reported by providers to LA Lead commissioner or joint | Fairly accurate, can tie to NHS number Payment driven, qualitative data on care home | Some people can’t see data as not anonymised, IG implications of different functions of organisations, NHS number |
| Oscar - Oscar Research Ltd (Public Service Exchange PSX - downloads, data feeds, reports and a mapping interface) | Housing (council tenants etc) Data dictionary | | |
| Joint Strategic Needs Assessment – Five domains Prevalence & Incidence Risk Factors Protective Factors Services Quality and Outcomes | Factors affecting health, wellbeing, and social care needs. Information and knowledge that local areas may use to build a picture of need. Includes indicators at local authority (county, unitary and district), CCG, STP; and GP and ward levels to enable comparison within as well as between local areas. | | |
| Blue badge recipients | | | |

## Slide 13
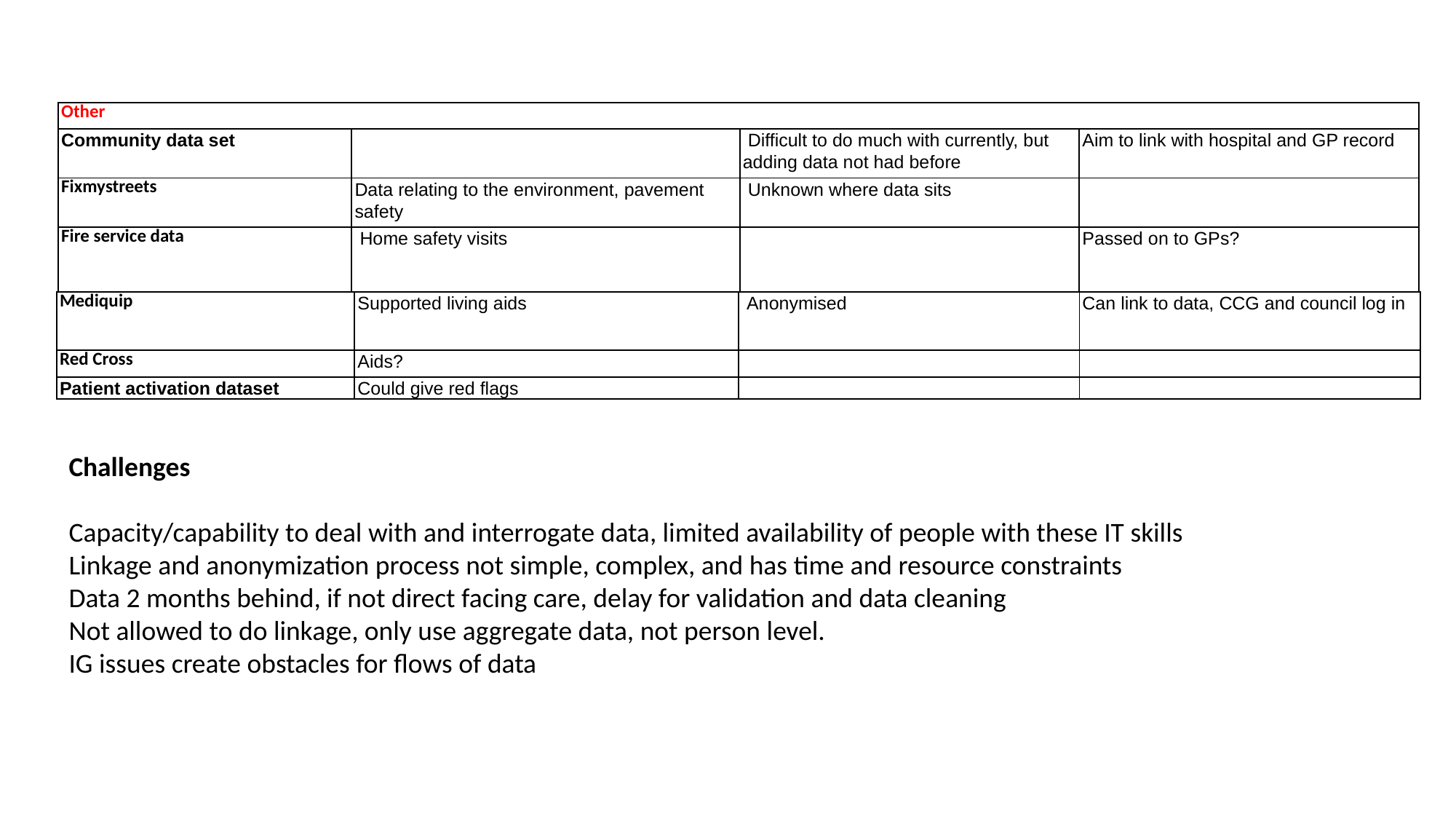

| Other | | | |
| --- | --- | --- | --- |
| Community data set | | Difficult to do much with currently, but adding data not had before | Aim to link with hospital and GP record |
| Fixmystreets | Data relating to the environment, pavement safety | Unknown where data sits | |
| Fire service data | Home safety visits | | Passed on to GPs? |
| Mediquip | Supported living aids | Anonymised | Can link to data, CCG and council log in |
| --- | --- | --- | --- |
| Red Cross | Aids? | | |
| Patient activation dataset | Could give red flags | | |
Challenges
Capacity/capability to deal with and interrogate data, limited availability of people with these IT skills
Linkage and anonymization process not simple, complex, and has time and resource constraints
Data 2 months behind, if not direct facing care, delay for validation and data cleaning
Not allowed to do linkage, only use aggregate data, not person level.
IG issues create obstacles for flows of data

## Slide 14
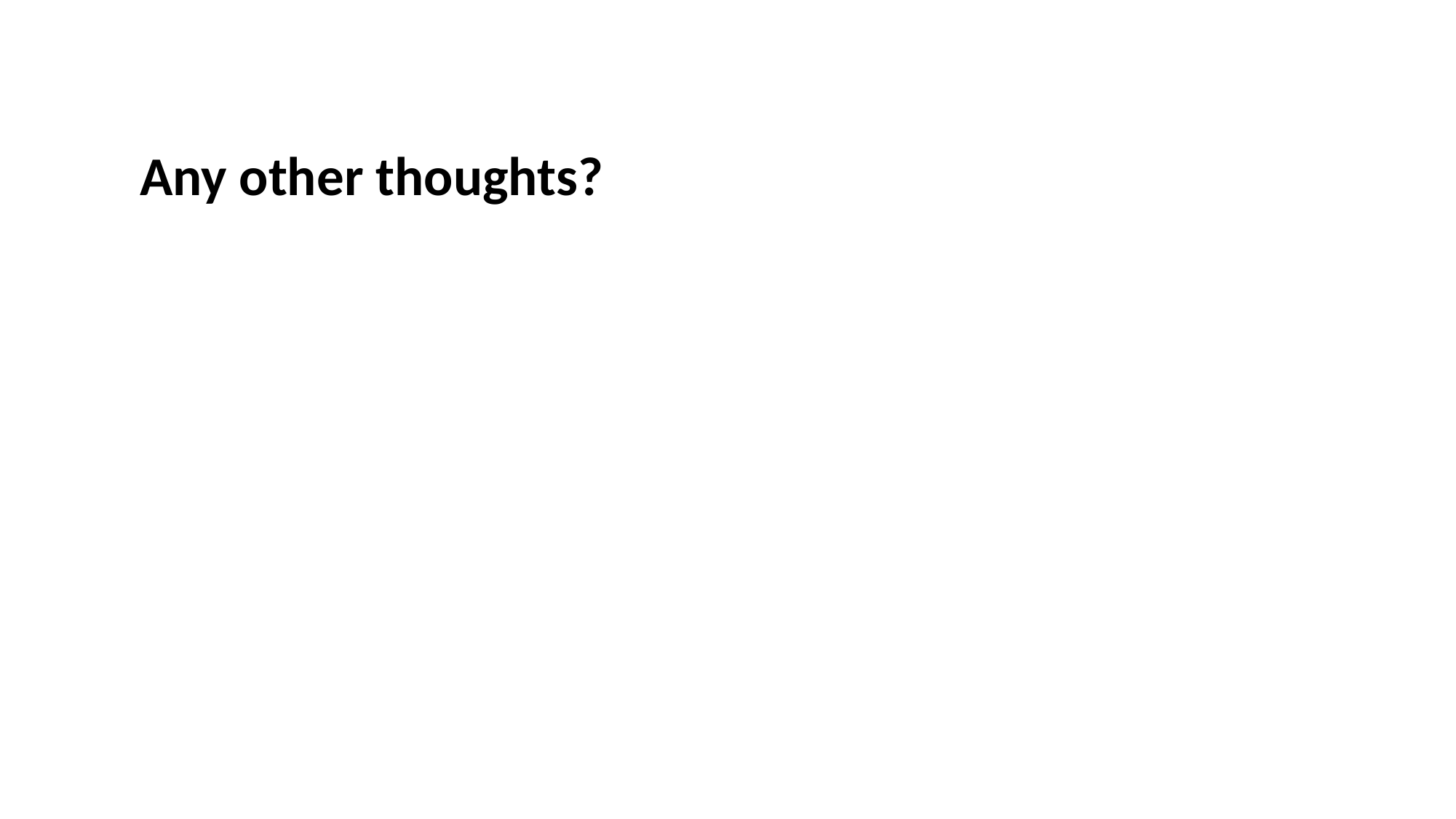

Any other thoughts?

## Slide 15
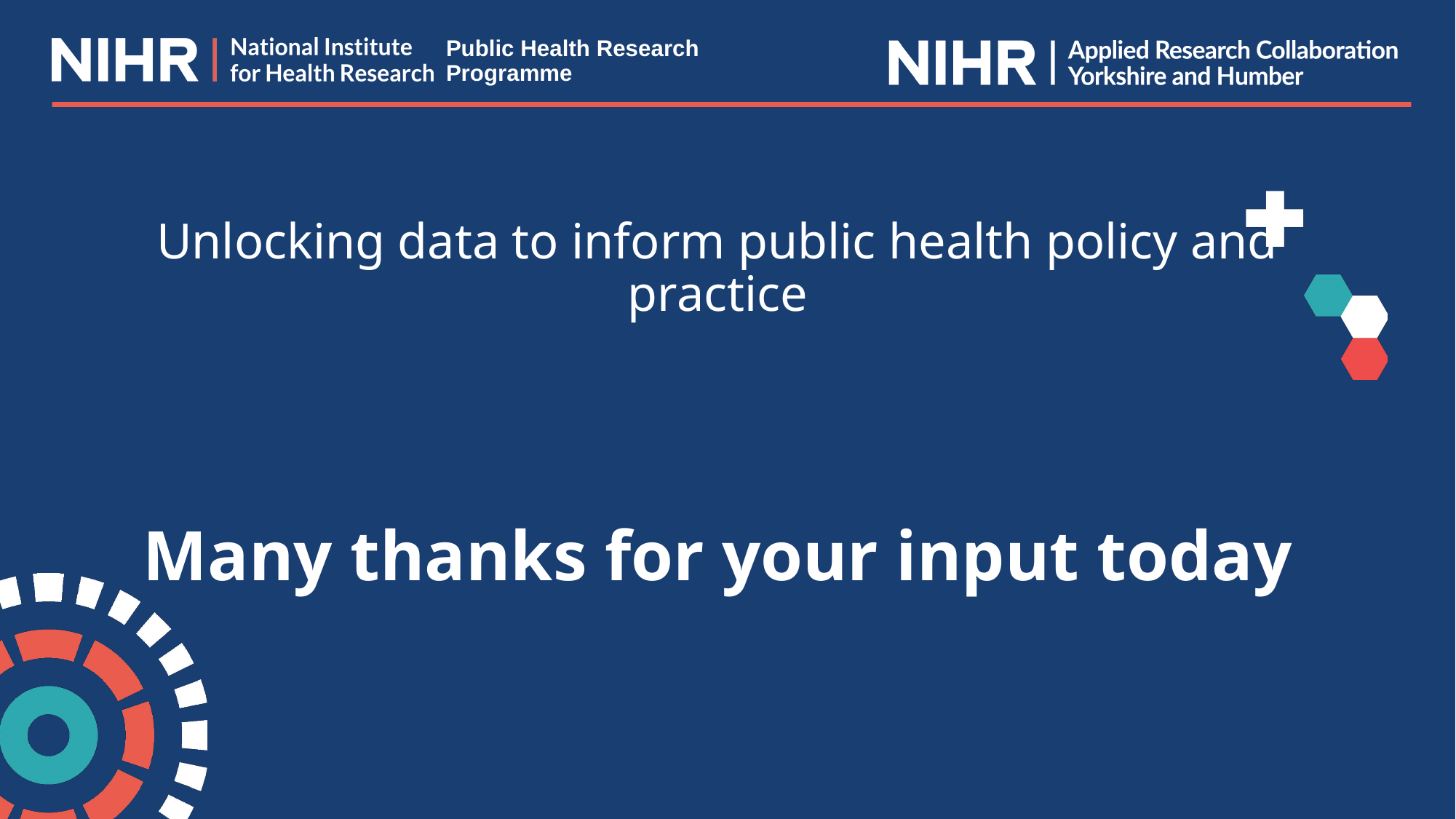

Public Health Research
Programme
# Unlocking data to inform public health policy and practice Many thanks for your input today

## Slide 16
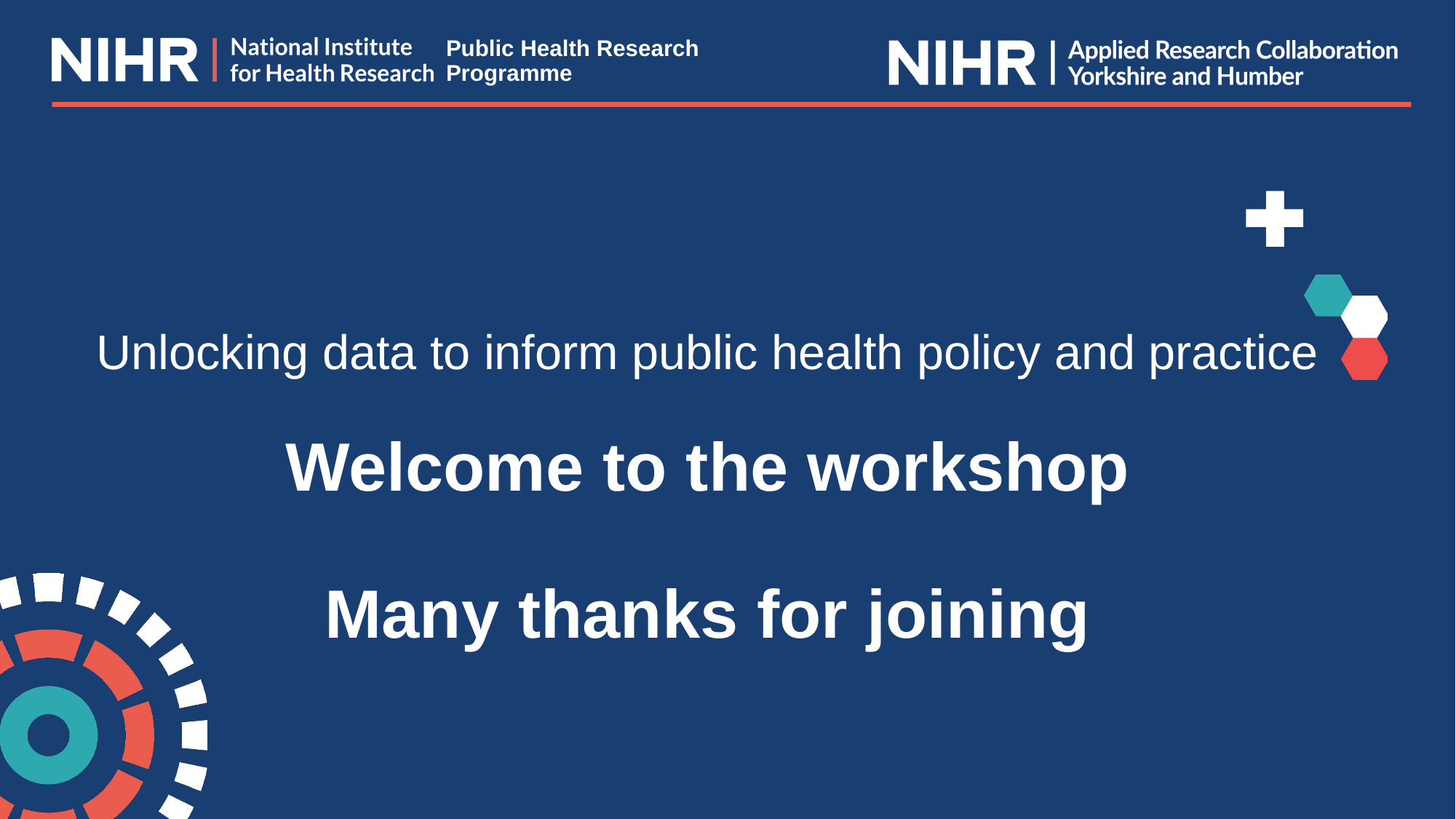

Public Health Research
Programme
# Unlocking data to inform public health policy and practice Welcome to the workshop Many thanks for joining

## Slide 17
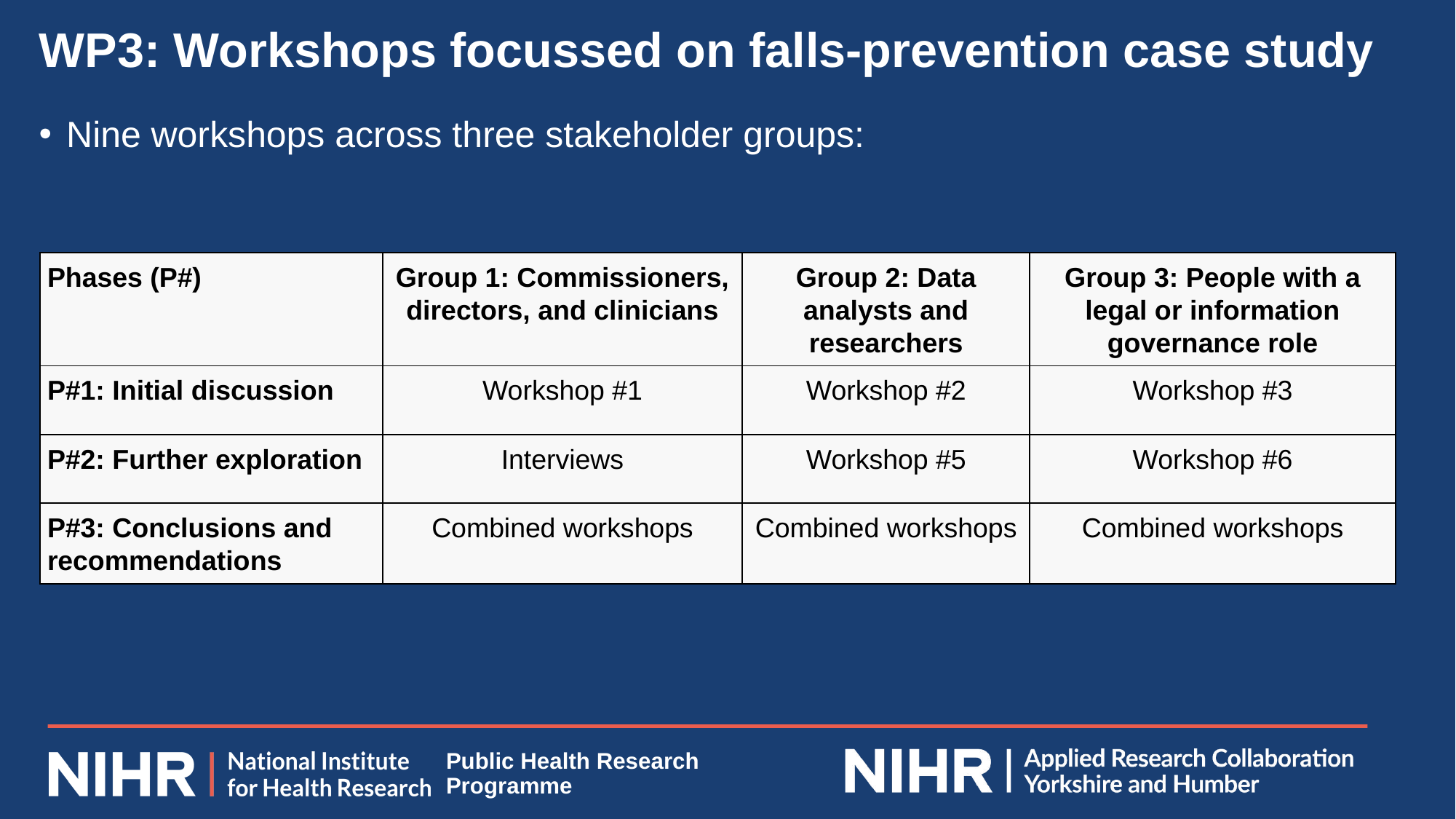

# WP3: Workshops focussed on falls-prevention case study
Nine workshops across three stakeholder groups:
| Phases (P#) | Group 1: Commissioners, directors, and clinicians | Group 2: Data analysts and researchers | Group 3: People with a legal or information governance role |
| --- | --- | --- | --- |
| P#1: Initial discussion | Workshop #1 | Workshop #2 | Workshop #3 |
| P#2: Further exploration | Interviews | Workshop #5 | Workshop #6 |
| P#3: Conclusions and recommendations | Combined workshops | Combined workshops | Combined workshops |
Public Health Research
Programme

## Slide 18
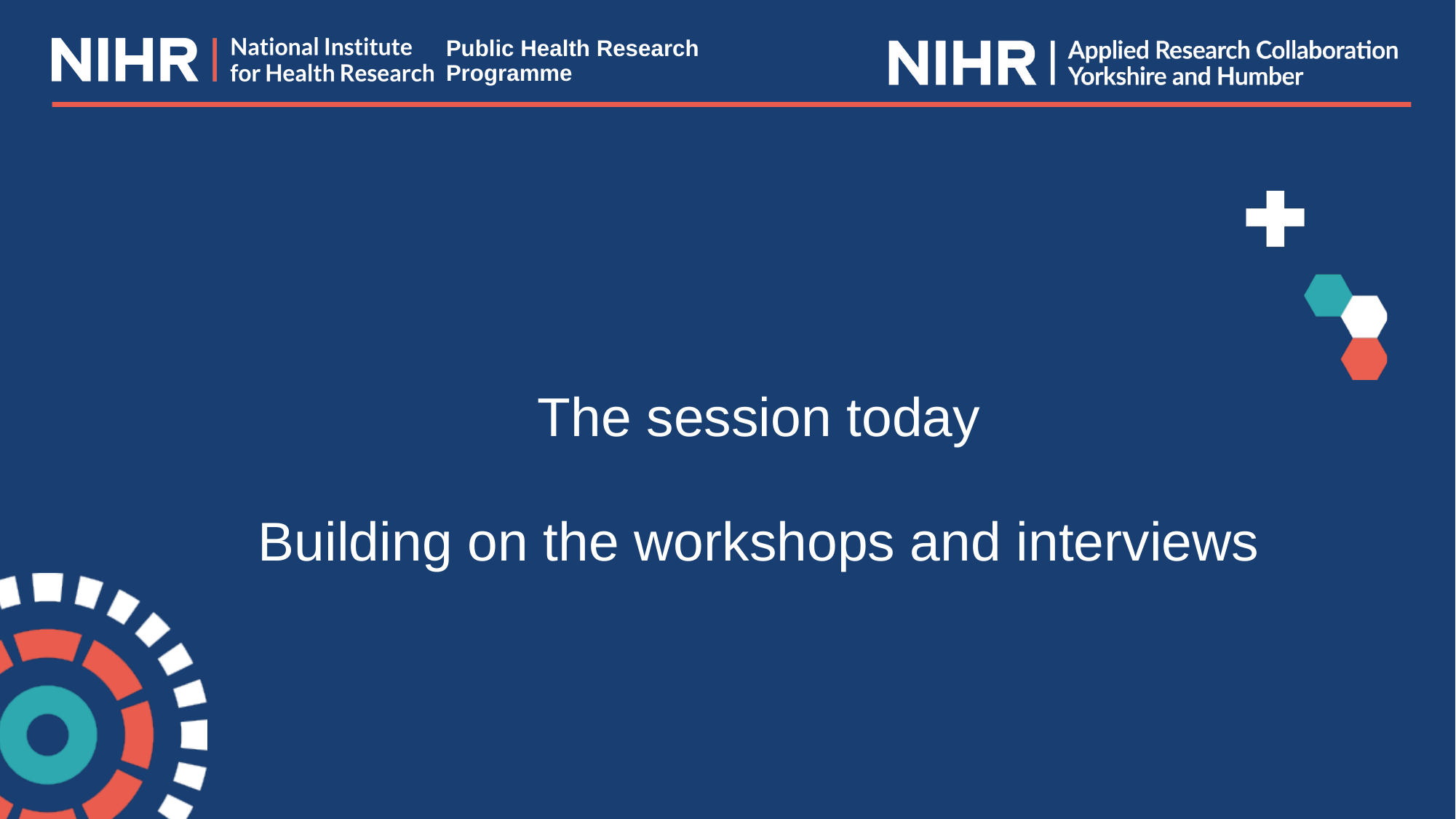

Public Health Research
Programme
# The session today
Building on the workshops and interviews

## Slide 19
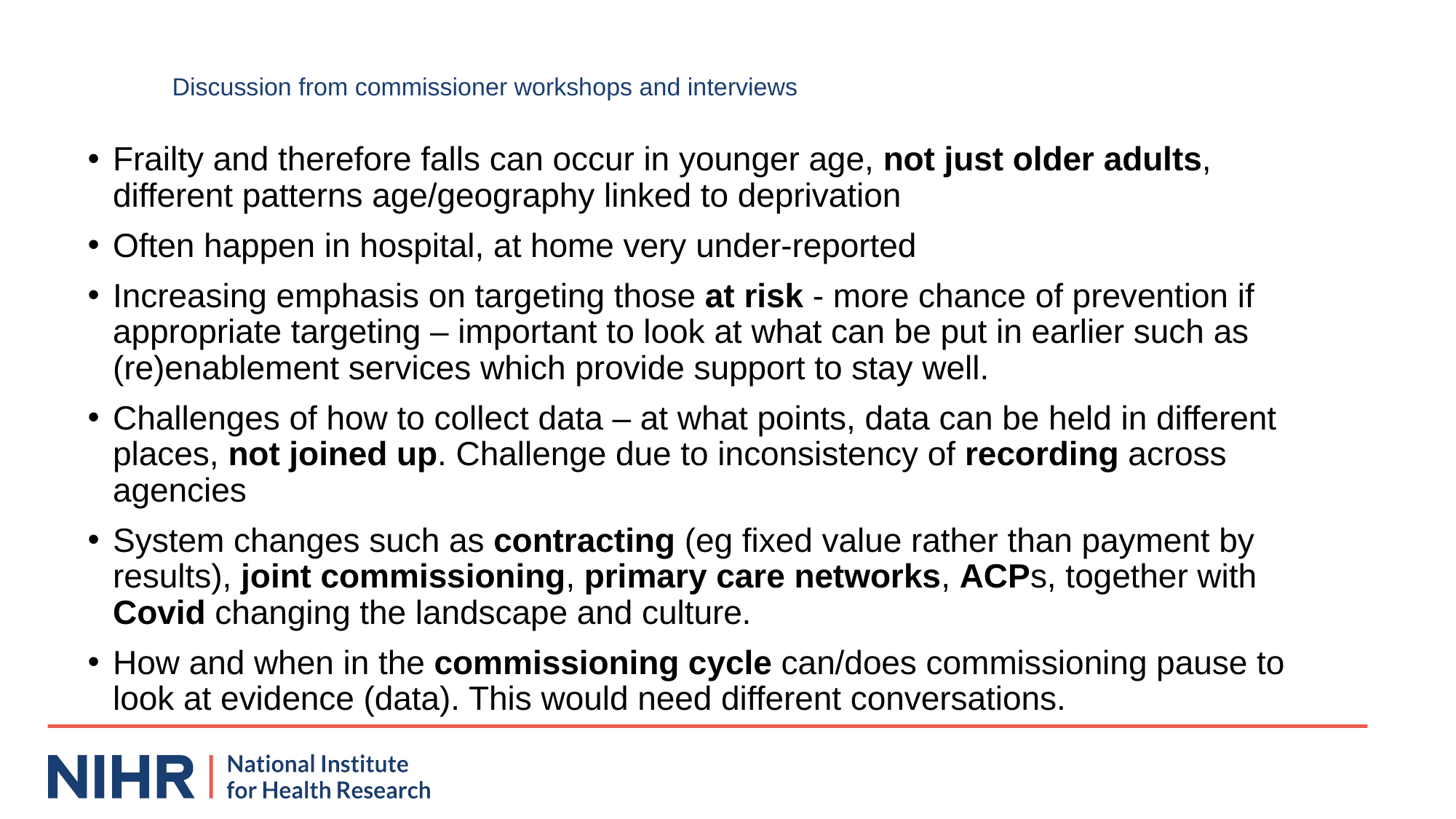

# Discussion from commissioner workshops and interviews
Frailty and therefore falls can occur in younger age, not just older adults, different patterns age/geography linked to deprivation
Often happen in hospital, at home very under-reported
Increasing emphasis on targeting those at risk - more chance of prevention if appropriate targeting – important to look at what can be put in earlier such as (re)enablement services which provide support to stay well.
Challenges of how to collect data – at what points, data can be held in different places, not joined up. Challenge due to inconsistency of recording across agencies
System changes such as contracting (eg fixed value rather than payment by results), joint commissioning, primary care networks, ACPs, together with Covid changing the landscape and culture.
How and when in the commissioning cycle can/does commissioning pause to look at evidence (data). This would need different conversations.

## Slide 20
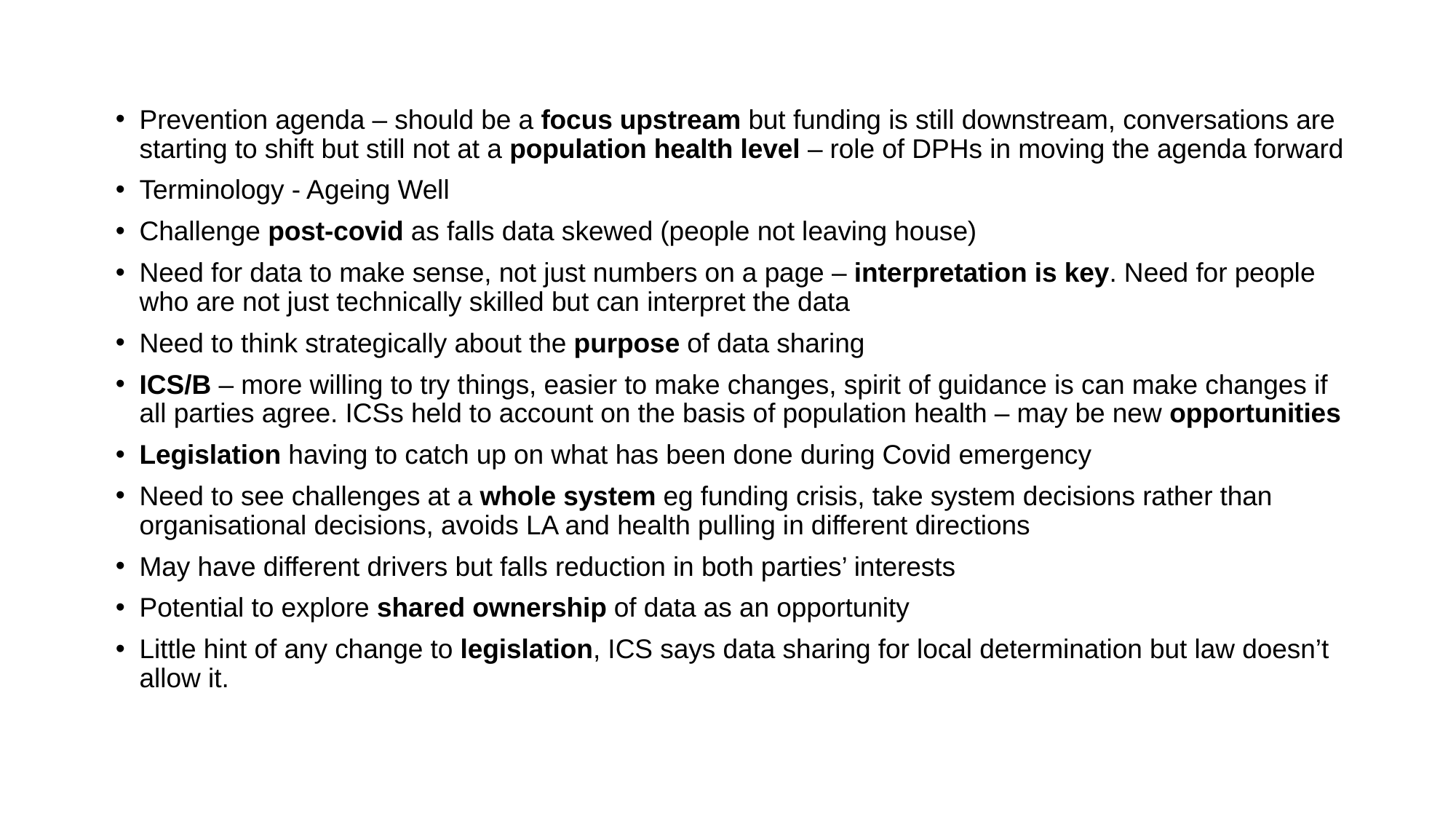

Prevention agenda – should be a focus upstream but funding is still downstream, conversations are starting to shift but still not at a population health level – role of DPHs in moving the agenda forward
Terminology - Ageing Well
Challenge post-covid as falls data skewed (people not leaving house)
Need for data to make sense, not just numbers on a page – interpretation is key. Need for people who are not just technically skilled but can interpret the data
Need to think strategically about the purpose of data sharing
ICS/B – more willing to try things, easier to make changes, spirit of guidance is can make changes if all parties agree. ICSs held to account on the basis of population health – may be new opportunities
Legislation having to catch up on what has been done during Covid emergency
Need to see challenges at a whole system eg funding crisis, take system decisions rather than organisational decisions, avoids LA and health pulling in different directions
May have different drivers but falls reduction in both parties’ interests
Potential to explore shared ownership of data as an opportunity
Little hint of any change to legislation, ICS says data sharing for local determination but law doesn’t allow it.

## Slide 21
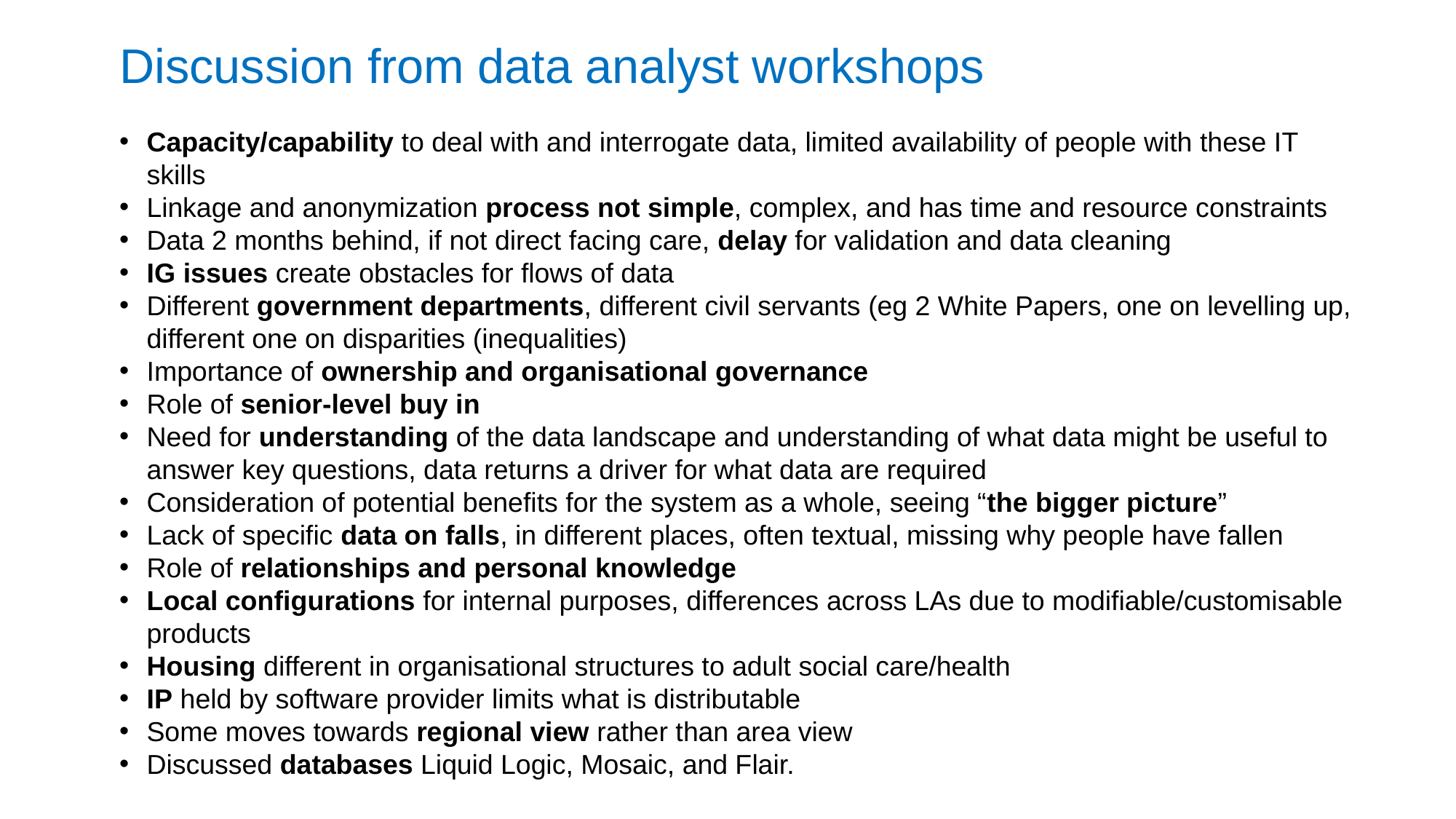

# Discussion from data analyst workshops
Capacity/capability to deal with and interrogate data, limited availability of people with these IT skills
Linkage and anonymization process not simple, complex, and has time and resource constraints
Data 2 months behind, if not direct facing care, delay for validation and data cleaning
IG issues create obstacles for flows of data
Different government departments, different civil servants (eg 2 White Papers, one on levelling up, different one on disparities (inequalities)
Importance of ownership and organisational governance
Role of senior-level buy in
Need for understanding of the data landscape and understanding of what data might be useful to answer key questions, data returns a driver for what data are required
Consideration of potential benefits for the system as a whole, seeing “the bigger picture”
Lack of specific data on falls, in different places, often textual, missing why people have fallen
Role of relationships and personal knowledge
Local configurations for internal purposes, differences across LAs due to modifiable/customisable products
Housing different in organisational structures to adult social care/health
IP held by software provider limits what is distributable
Some moves towards regional view rather than area view
Discussed databases Liquid Logic, Mosaic, and Flair.

## Slide 22
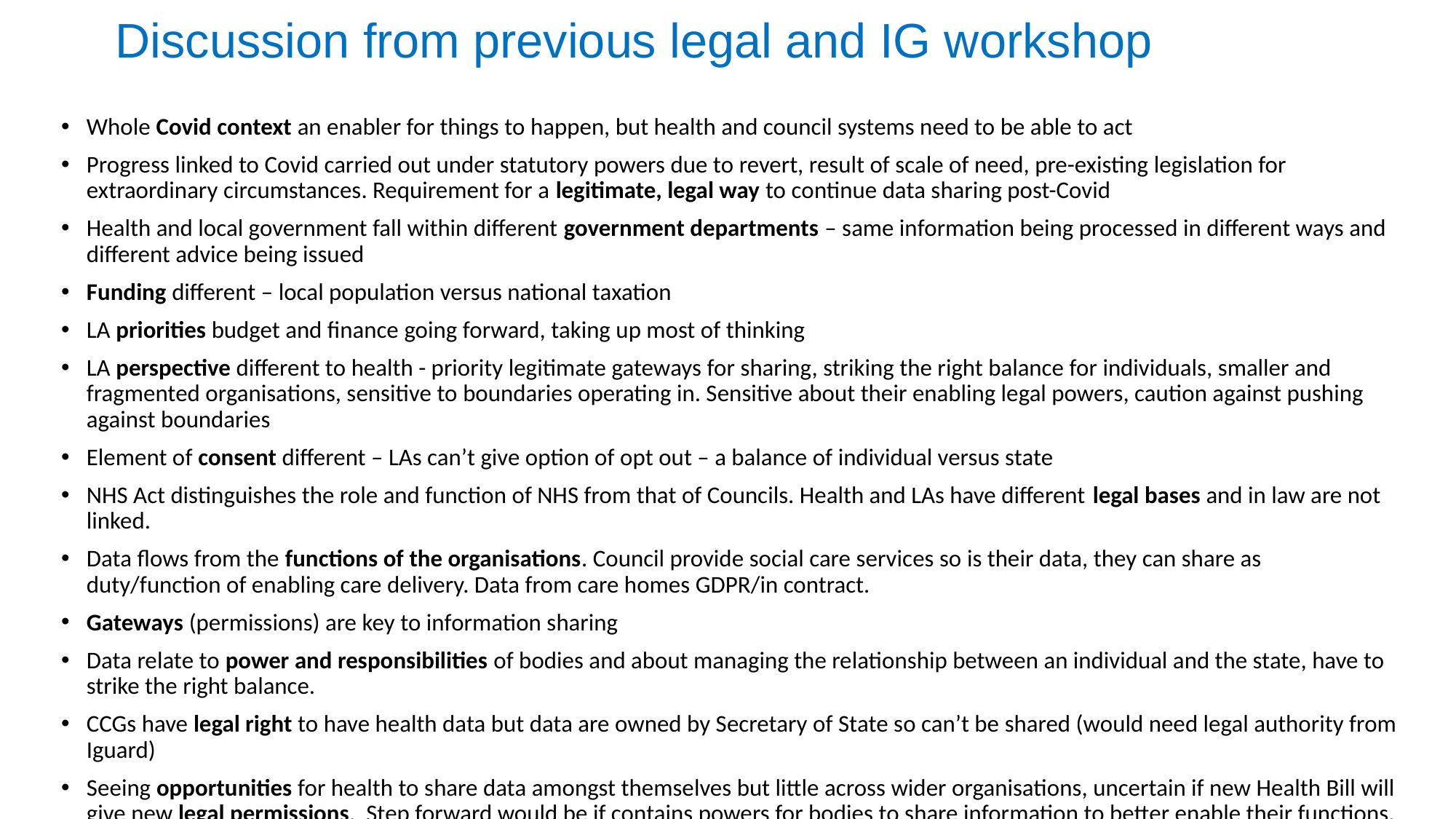

# Discussion from previous legal and IG workshop
Whole Covid context an enabler for things to happen, but health and council systems need to be able to act
Progress linked to Covid carried out under statutory powers due to revert, result of scale of need, pre-existing legislation for extraordinary circumstances. Requirement for a legitimate, legal way to continue data sharing post-Covid
Health and local government fall within different government departments – same information being processed in different ways and different advice being issued
Funding different – local population versus national taxation
LA priorities budget and finance going forward, taking up most of thinking
LA perspective different to health - priority legitimate gateways for sharing, striking the right balance for individuals, smaller and fragmented organisations, sensitive to boundaries operating in. Sensitive about their enabling legal powers, caution against pushing against boundaries
Element of consent different – LAs can’t give option of opt out – a balance of individual versus state
NHS Act distinguishes the role and function of NHS from that of Councils. Health and LAs have different legal bases and in law are not linked.
Data flows from the functions of the organisations. Council provide social care services so is their data, they can share as duty/function of enabling care delivery. Data from care homes GDPR/in contract.
Gateways (permissions) are key to information sharing
Data relate to power and responsibilities of bodies and about managing the relationship between an individual and the state, have to strike the right balance.
CCGs have legal right to have health data but data are owned by Secretary of State so can’t be shared (would need legal authority from Iguard)
Seeing opportunities for health to share data amongst themselves but little across wider organisations, uncertain if new Health Bill will give new legal permissions. Step forward would be if contains powers for bodies to share information to better enable their functions.

## Slide 23
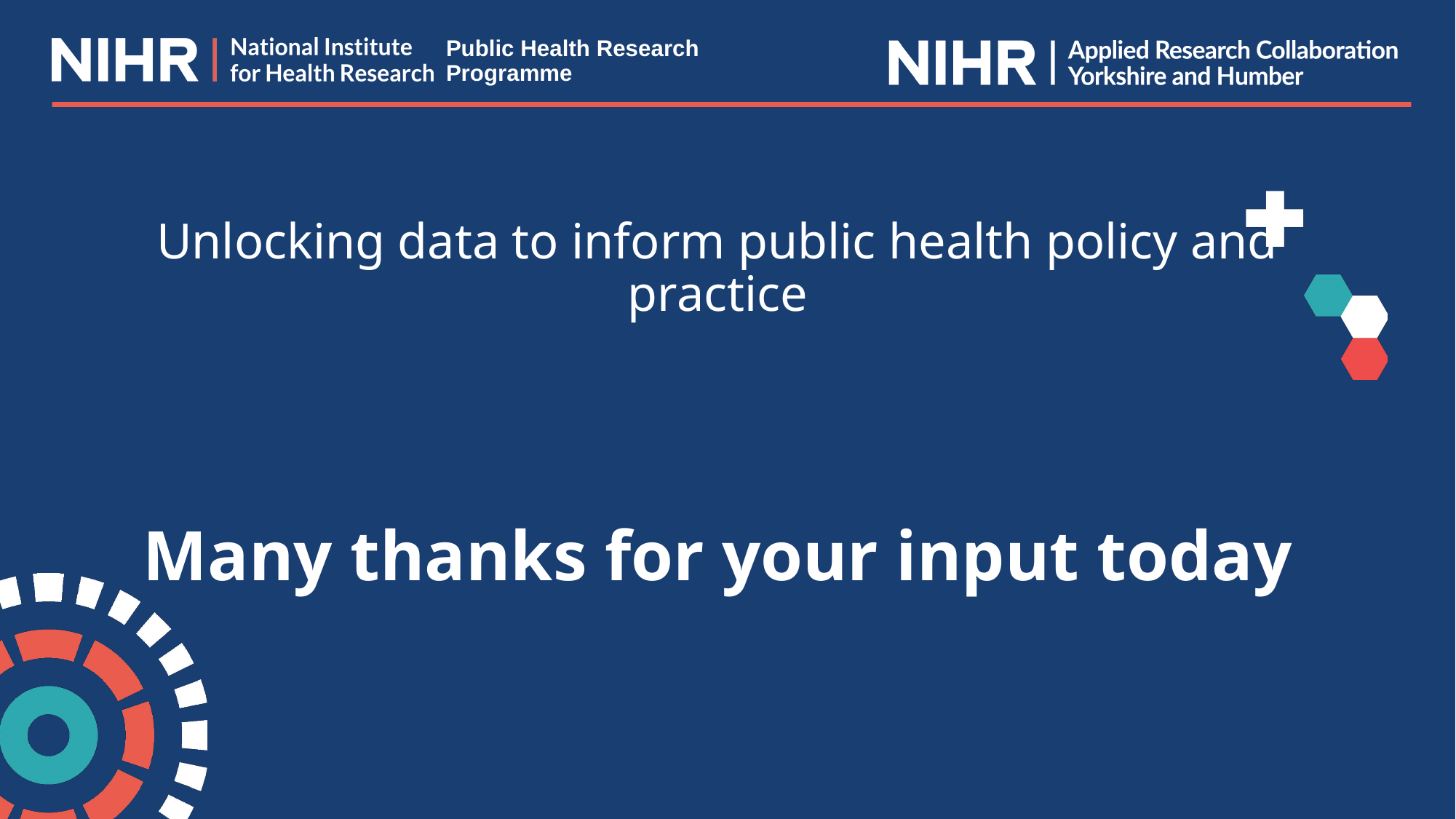

Public Health Research
Programme
# Unlocking data to inform public health policy and practice Many thanks for your input today

## Slide 24
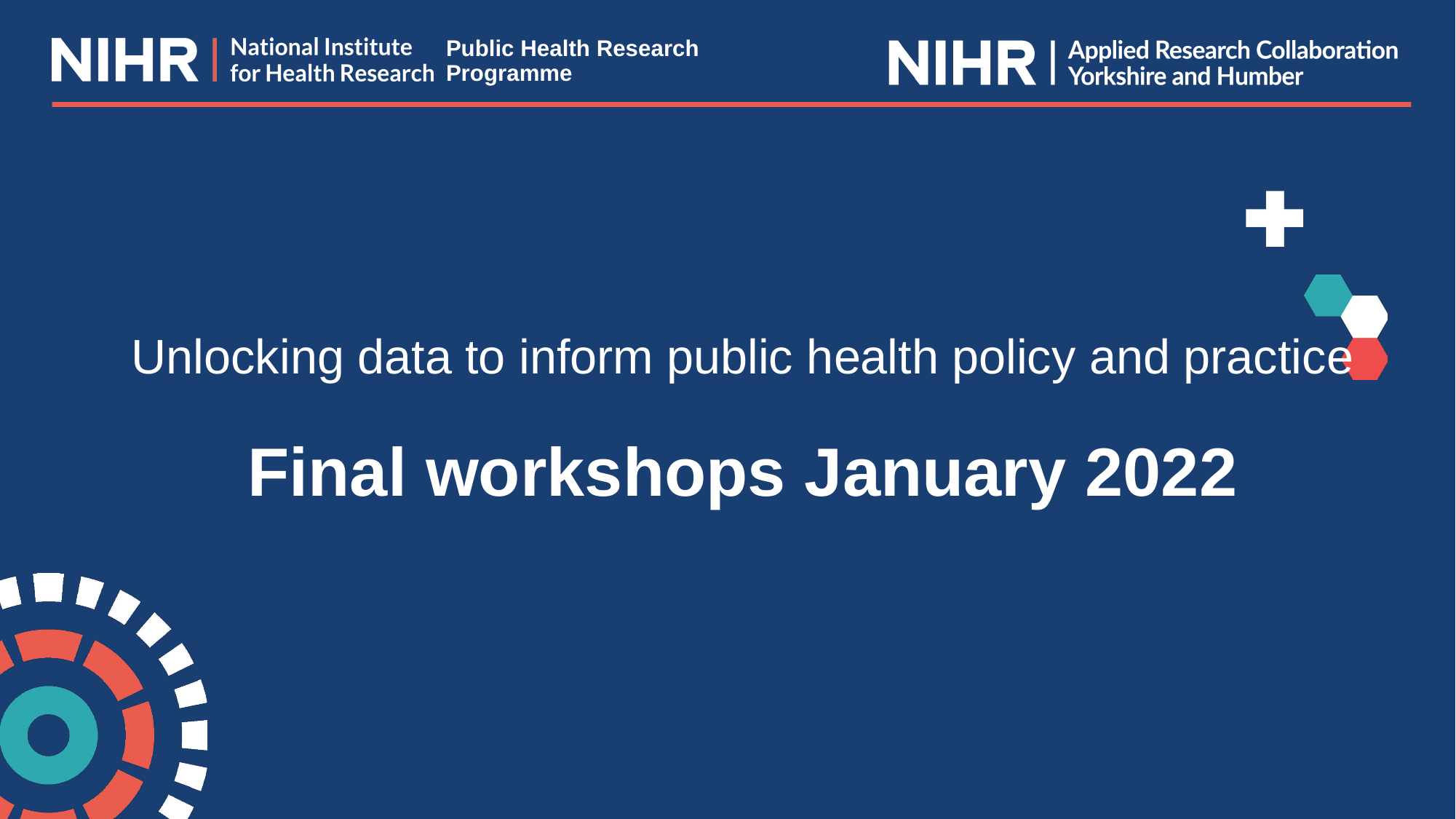

Public Health Research
Programme
# Unlocking data to inform public health policy and practice Final workshops January 2022

## Slide 25
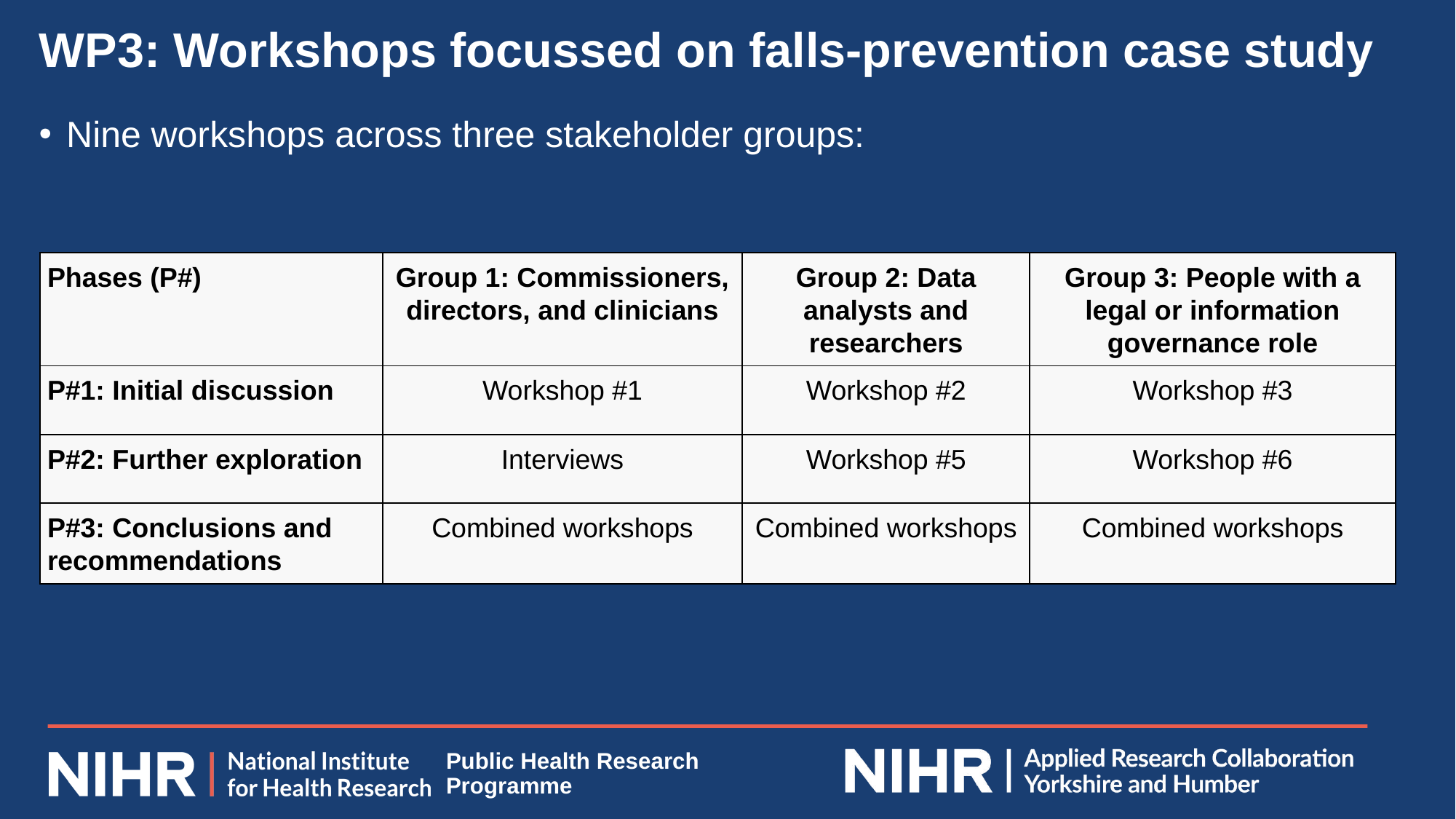

# WP3: Workshops focussed on falls-prevention case study
Nine workshops across three stakeholder groups:
| Phases (P#) | Group 1: Commissioners, directors, and clinicians | Group 2: Data analysts and researchers | Group 3: People with a legal or information governance role |
| --- | --- | --- | --- |
| P#1: Initial discussion | Workshop #1 | Workshop #2 | Workshop #3 |
| P#2: Further exploration | Interviews | Workshop #5 | Workshop #6 |
| P#3: Conclusions and recommendations | Combined workshops | Combined workshops | Combined workshops |
Public Health Research
Programme

## Slide 26
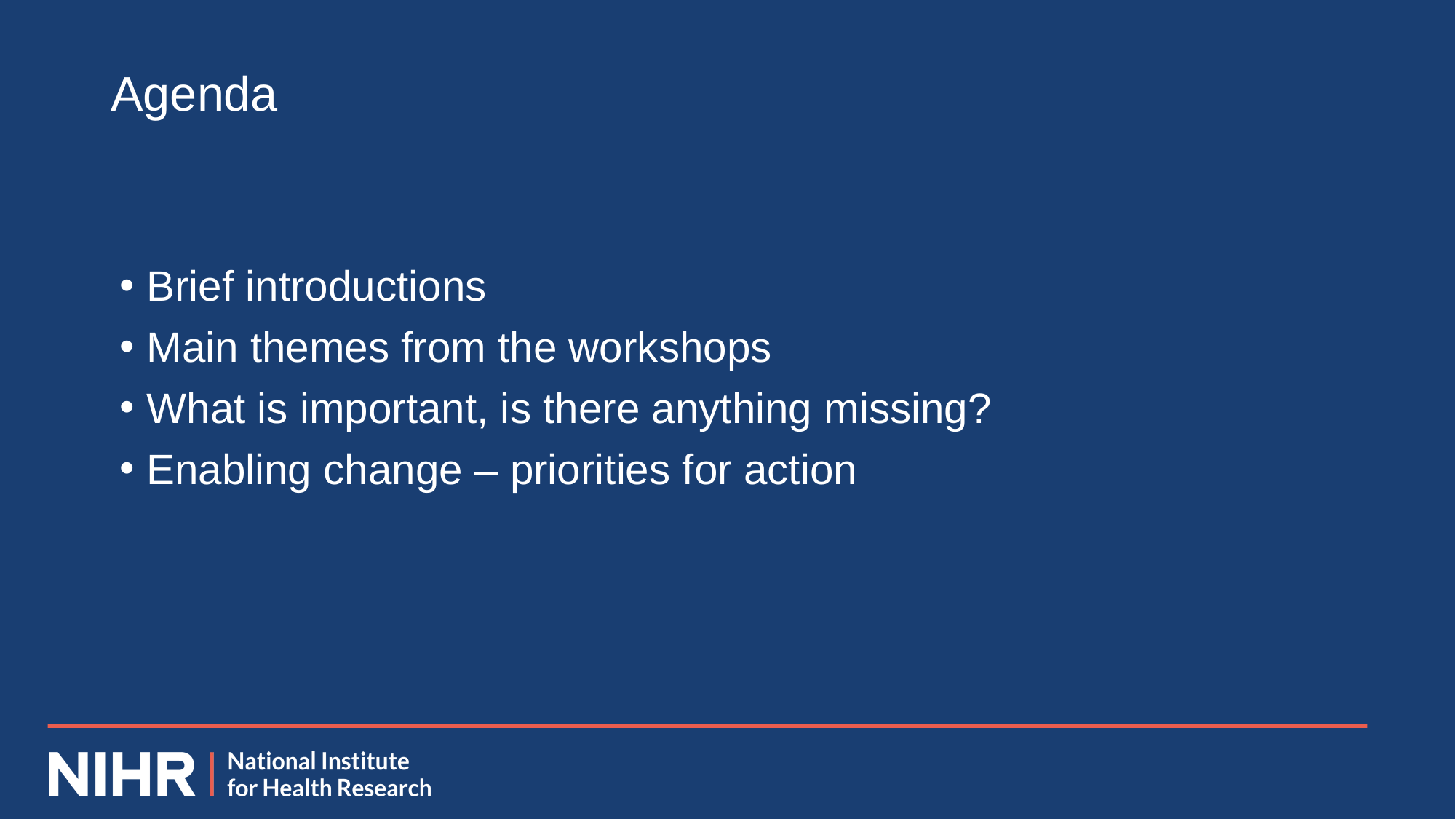

# Agenda
Brief introductions
Main themes from the workshops
What is important, is there anything missing?
Enabling change – priorities for action

## Slide 27
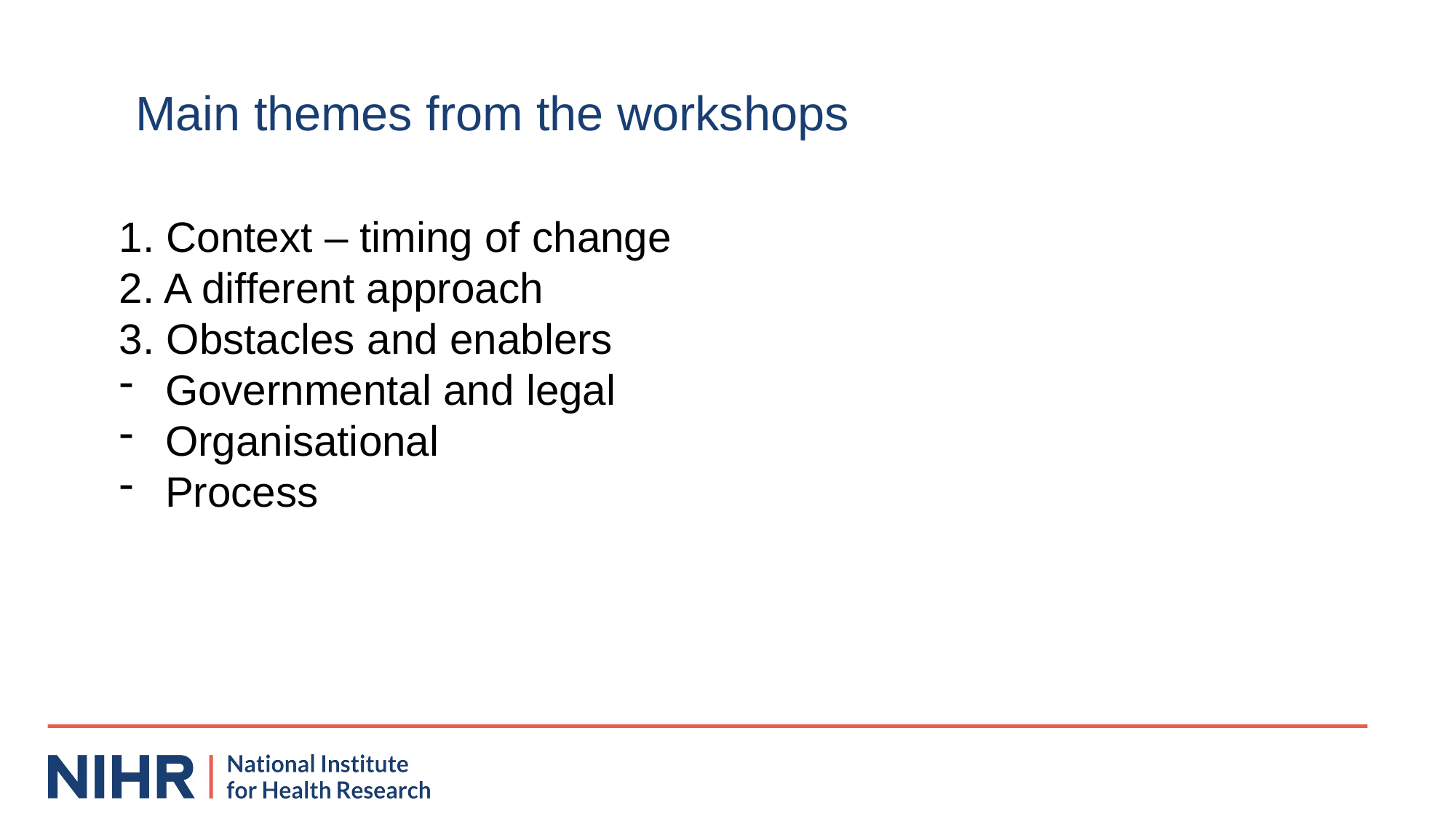

# Main themes from the workshops
1. Context – timing of change
2. A different approach
3. Obstacles and enablers
 Governmental and legal
 Organisational
 Process

## Slide 28
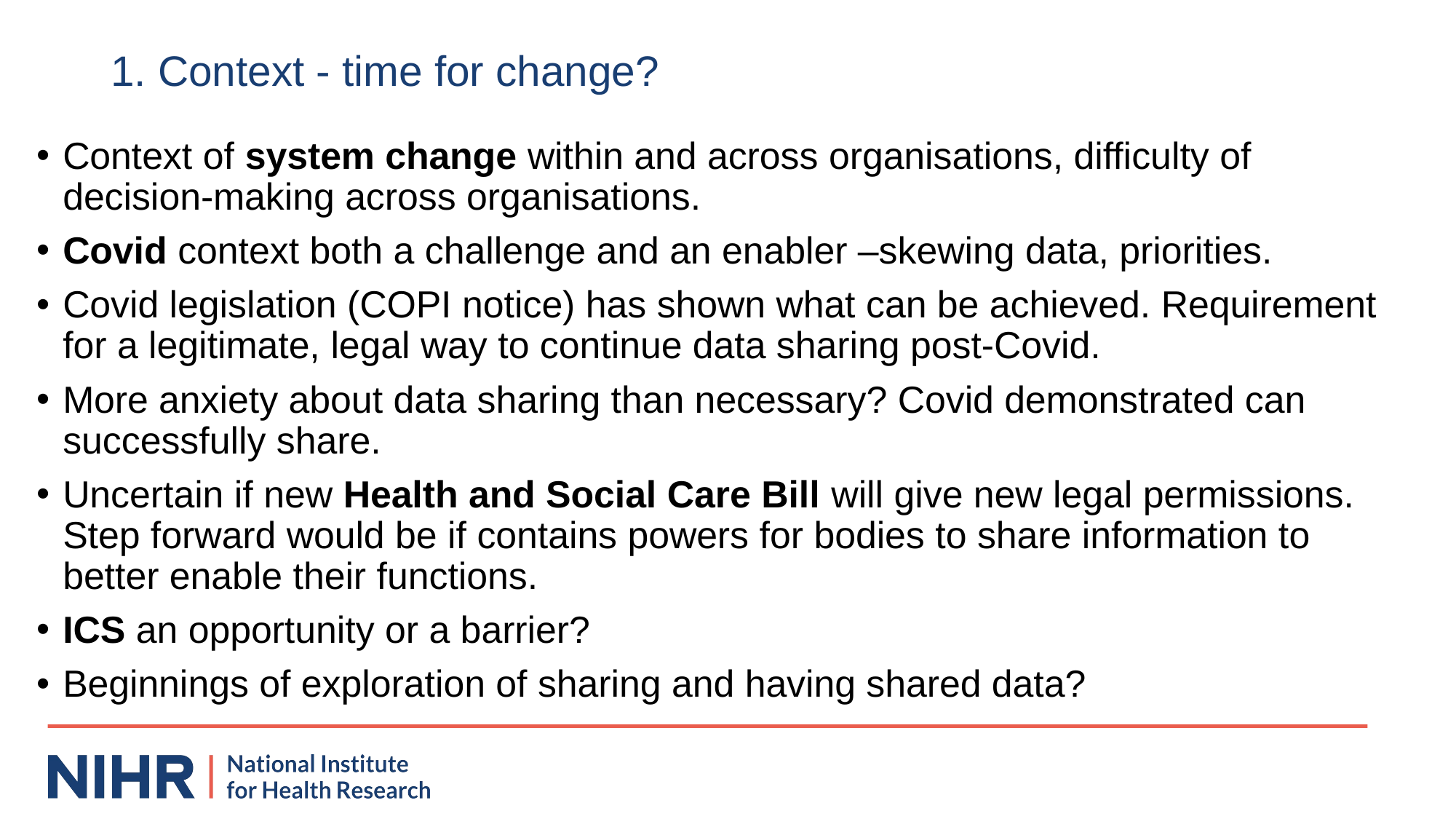

# 1. Context - time for change?
Context of system change within and across organisations, difficulty of decision-making across organisations.
Covid context both a challenge and an enabler –skewing data, priorities.
Covid legislation (COPI notice) has shown what can be achieved. Requirement for a legitimate, legal way to continue data sharing post-Covid.
More anxiety about data sharing than necessary? Covid demonstrated can successfully share.
Uncertain if new Health and Social Care Bill will give new legal permissions. Step forward would be if contains powers for bodies to share information to better enable their functions.
ICS an opportunity or a barrier?
Beginnings of exploration of sharing and having shared data?

## Slide 29
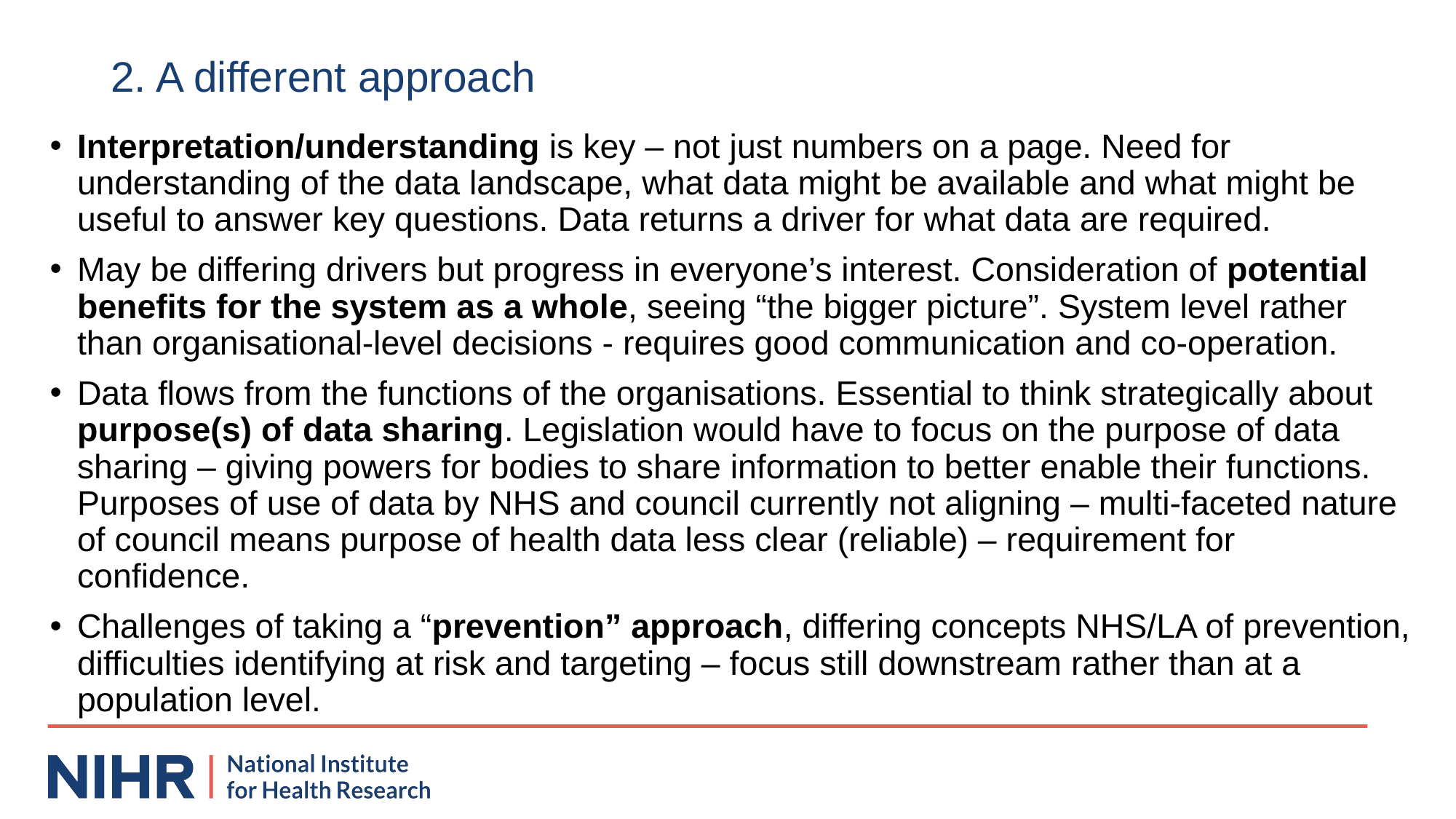

# 2. A different approach
Interpretation/understanding is key – not just numbers on a page. Need for understanding of the data landscape, what data might be available and what might be useful to answer key questions. Data returns a driver for what data are required.
May be differing drivers but progress in everyone’s interest. Consideration of potential benefits for the system as a whole, seeing “the bigger picture”. System level rather than organisational-level decisions - requires good communication and co-operation.
Data flows from the functions of the organisations. Essential to think strategically about purpose(s) of data sharing. Legislation would have to focus on the purpose of data sharing – giving powers for bodies to share information to better enable their functions. Purposes of use of data by NHS and council currently not aligning – multi-faceted nature of council means purpose of health data less clear (reliable) – requirement for confidence.
Challenges of taking a “prevention” approach, differing concepts NHS/LA of prevention, difficulties identifying at risk and targeting – focus still downstream rather than at a population level.

## Slide 30
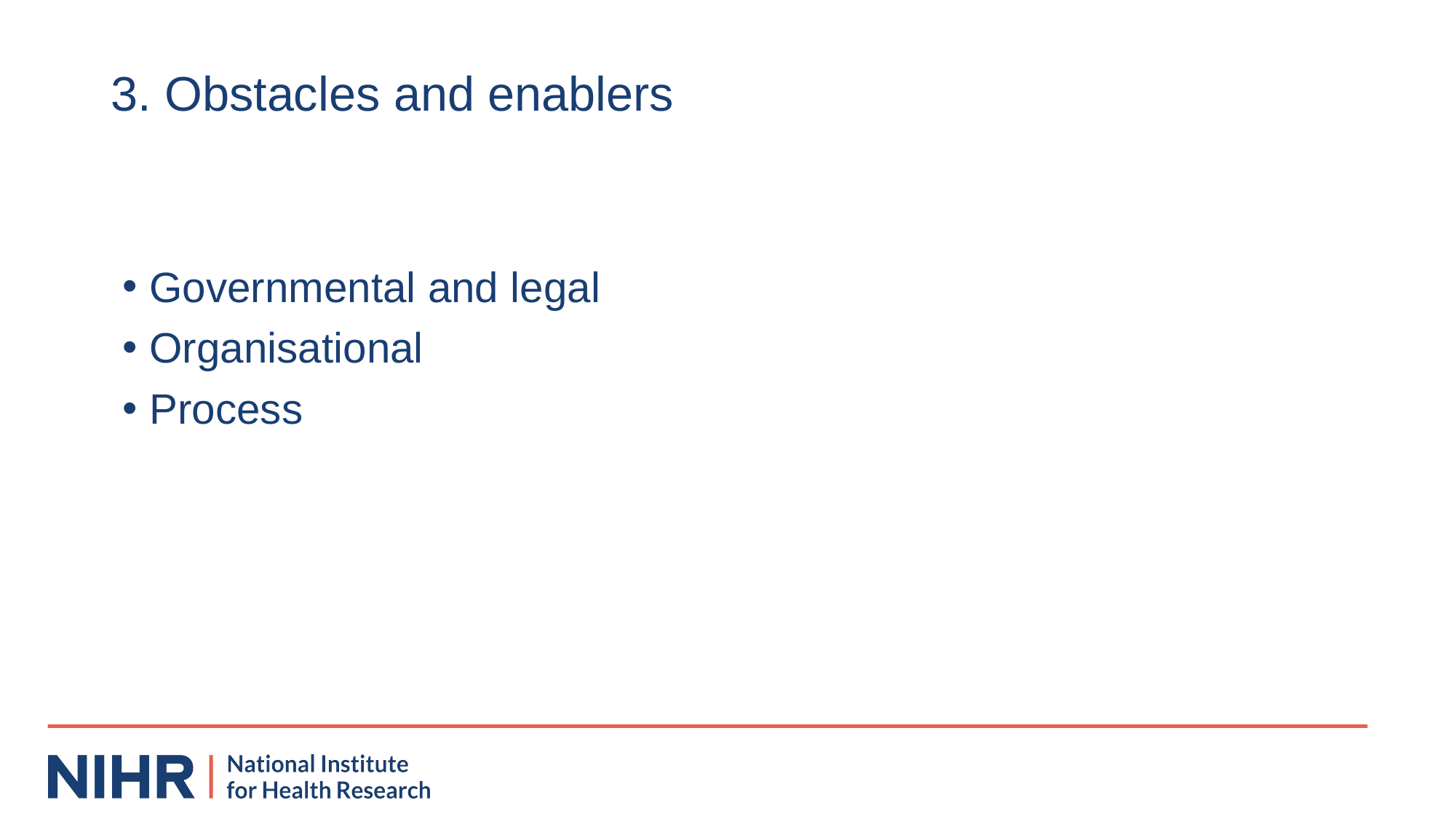

# 3. Obstacles and enablers
Governmental and legal
Organisational
Process

## Slide 31
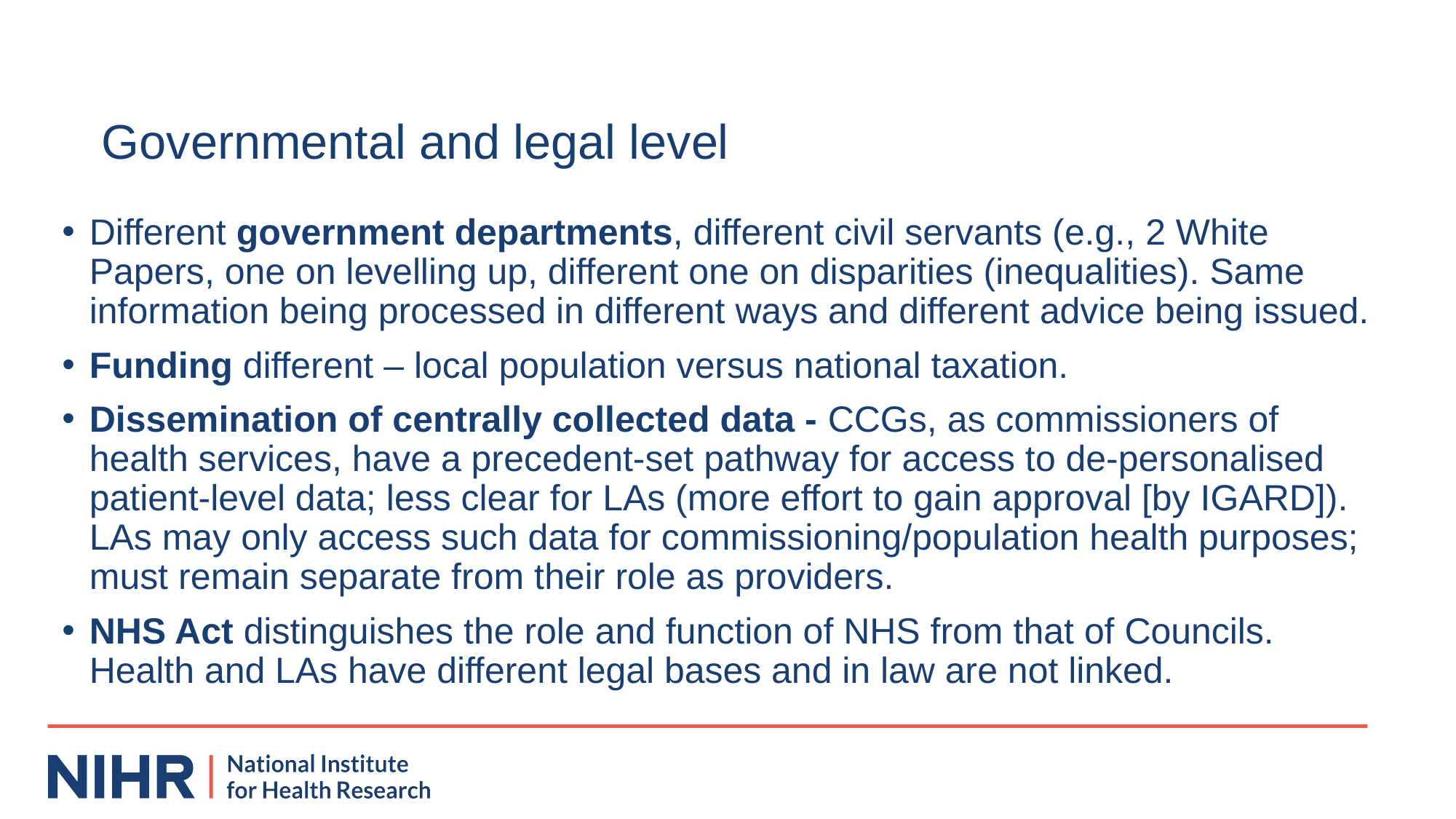

# Governmental and legal level
Different government departments, different civil servants (e.g., 2 White Papers, one on levelling up, different one on disparities (inequalities). Same information being processed in different ways and different advice being issued.
Funding different – local population versus national taxation.
Dissemination of centrally collected data - CCGs, as commissioners of health services, have a precedent-set pathway for access to de-personalised patient-level data; less clear for LAs (more effort to gain approval [by IGARD]). LAs may only access such data for commissioning/population health purposes; must remain separate from their role as providers.
NHS Act distinguishes the role and function of NHS from that of Councils. Health and LAs have different legal bases and in law are not linked.

## Slide 32
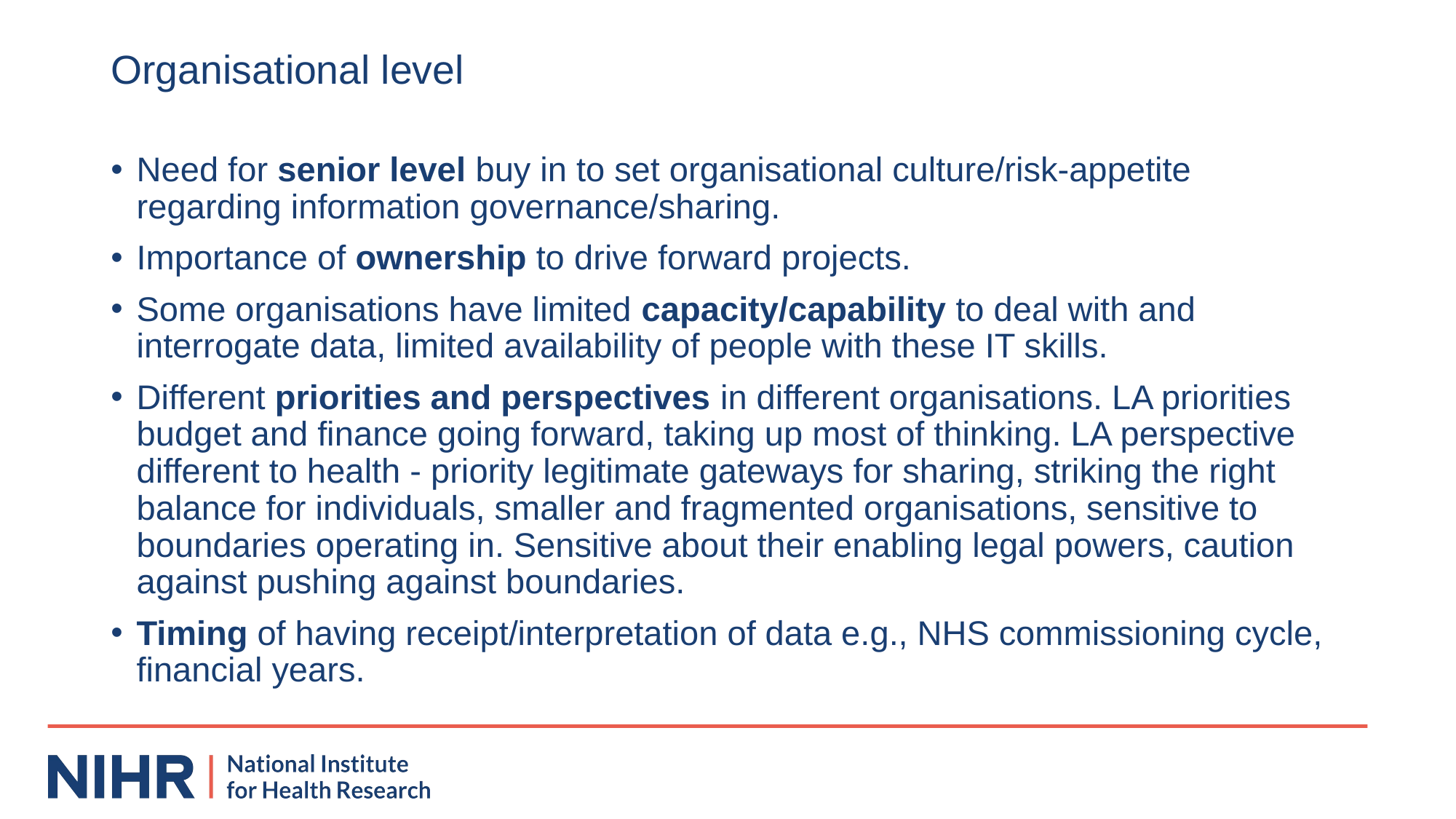

# Organisational level
Need for senior level buy in to set organisational culture/risk-appetite regarding information governance/sharing.
Importance of ownership to drive forward projects.
Some organisations have limited capacity/capability to deal with and interrogate data, limited availability of people with these IT skills.
Different priorities and perspectives in different organisations. LA priorities budget and finance going forward, taking up most of thinking. LA perspective different to health - priority legitimate gateways for sharing, striking the right balance for individuals, smaller and fragmented organisations, sensitive to boundaries operating in. Sensitive about their enabling legal powers, caution against pushing against boundaries.
Timing of having receipt/interpretation of data e.g., NHS commissioning cycle, financial years.

## Slide 33
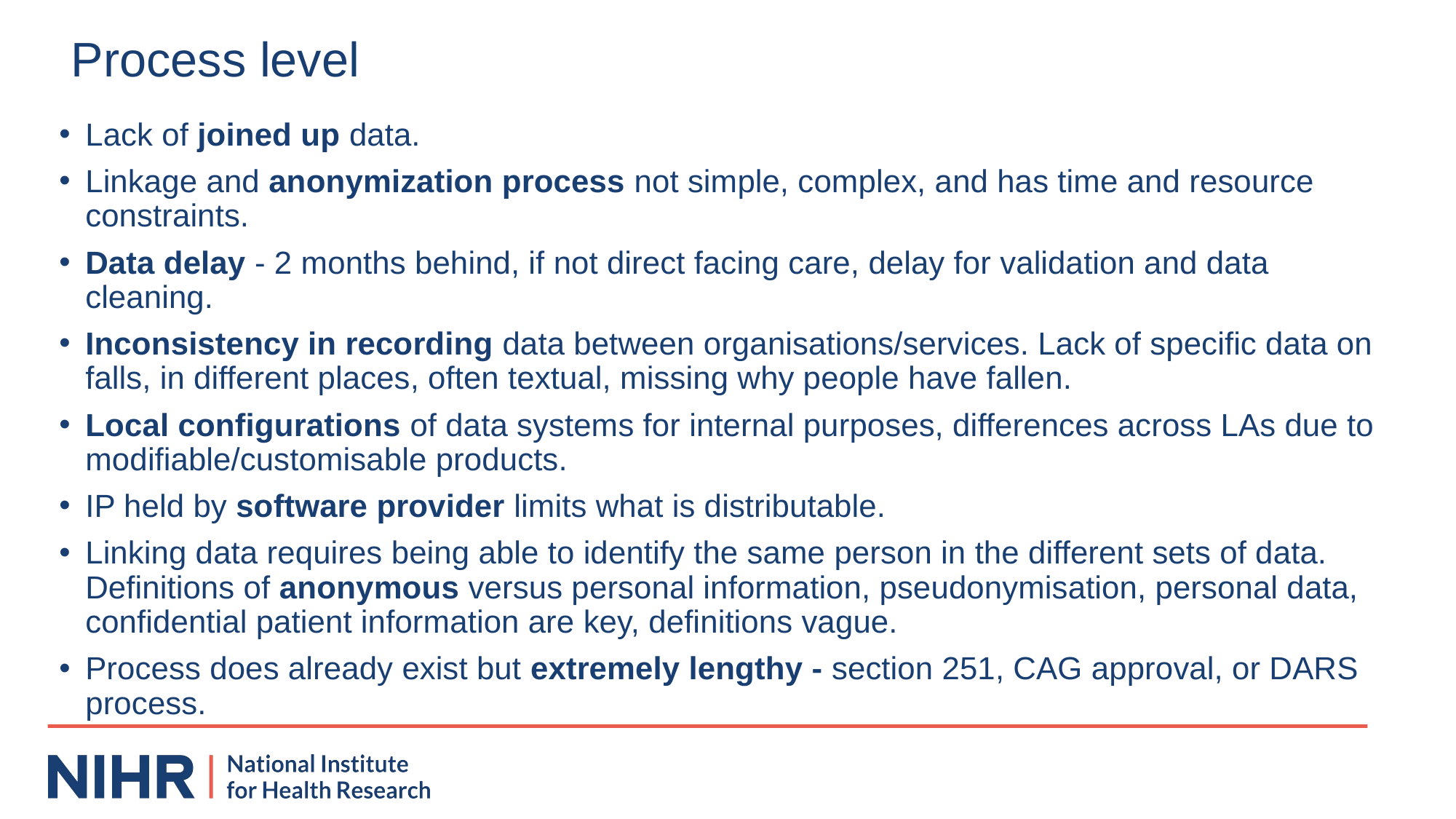

# Process level
Lack of joined up data.
Linkage and anonymization process not simple, complex, and has time and resource constraints.
Data delay - 2 months behind, if not direct facing care, delay for validation and data cleaning.
Inconsistency in recording data between organisations/services. Lack of specific data on falls, in different places, often textual, missing why people have fallen.
Local configurations of data systems for internal purposes, differences across LAs due to modifiable/customisable products.
IP held by software provider limits what is distributable.
Linking data requires being able to identify the same person in the different sets of data. Definitions of anonymous versus personal information, pseudonymisation, personal data, confidential patient information are key, definitions vague.
Process does already exist but extremely lengthy - section 251, CAG approval, or DARS process.

## Slide 34
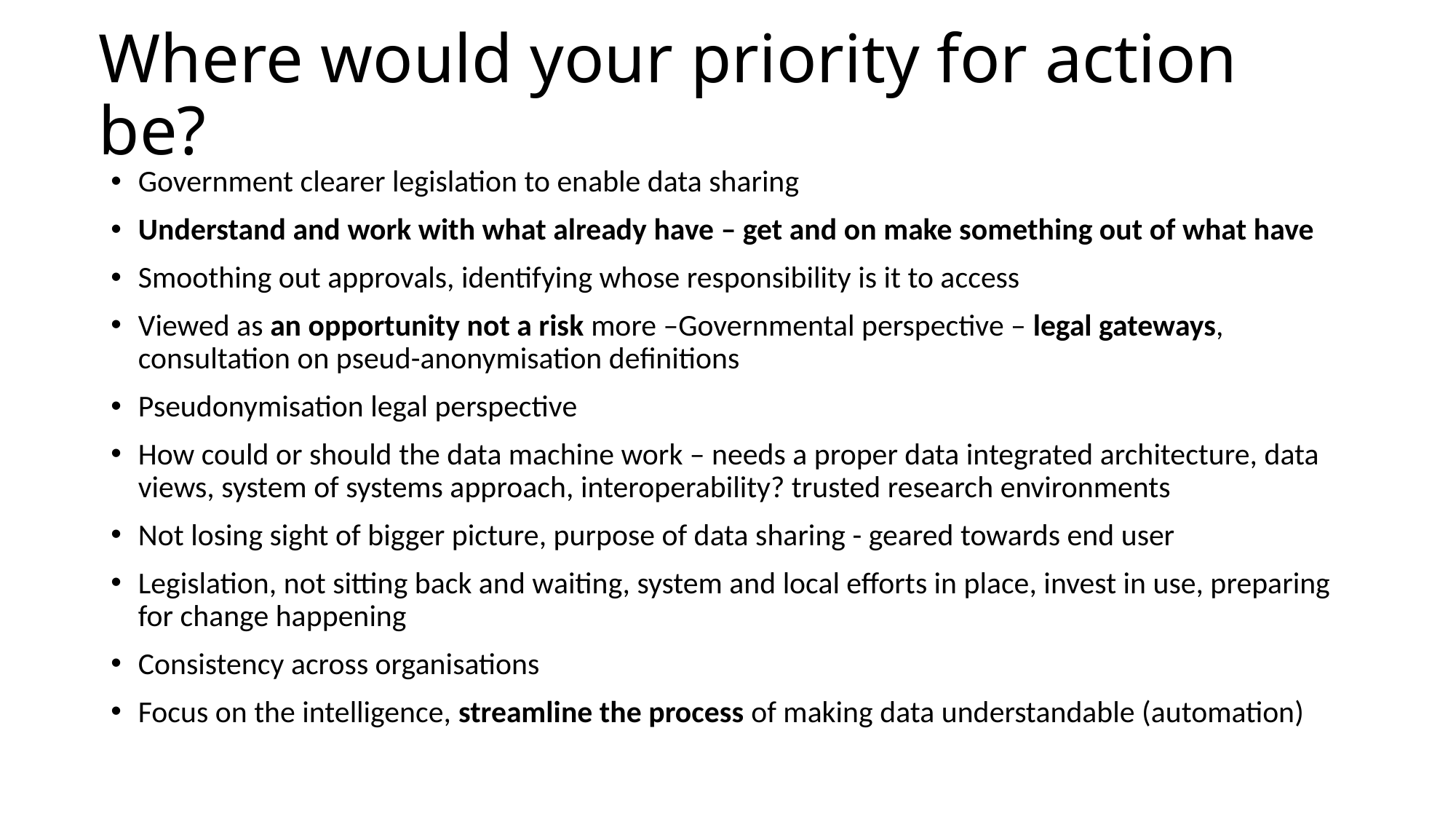

# Where would your priority for action be?
Government clearer legislation to enable data sharing
Understand and work with what already have – get and on make something out of what have
Smoothing out approvals, identifying whose responsibility is it to access
Viewed as an opportunity not a risk more –Governmental perspective – legal gateways, consultation on pseud-anonymisation definitions
Pseudonymisation legal perspective
How could or should the data machine work – needs a proper data integrated architecture, data views, system of systems approach, interoperability? trusted research environments
Not losing sight of bigger picture, purpose of data sharing - geared towards end user
Legislation, not sitting back and waiting, system and local efforts in place, invest in use, preparing for change happening
Consistency across organisations
Focus on the intelligence, streamline the process of making data understandable (automation)

## Slide 35
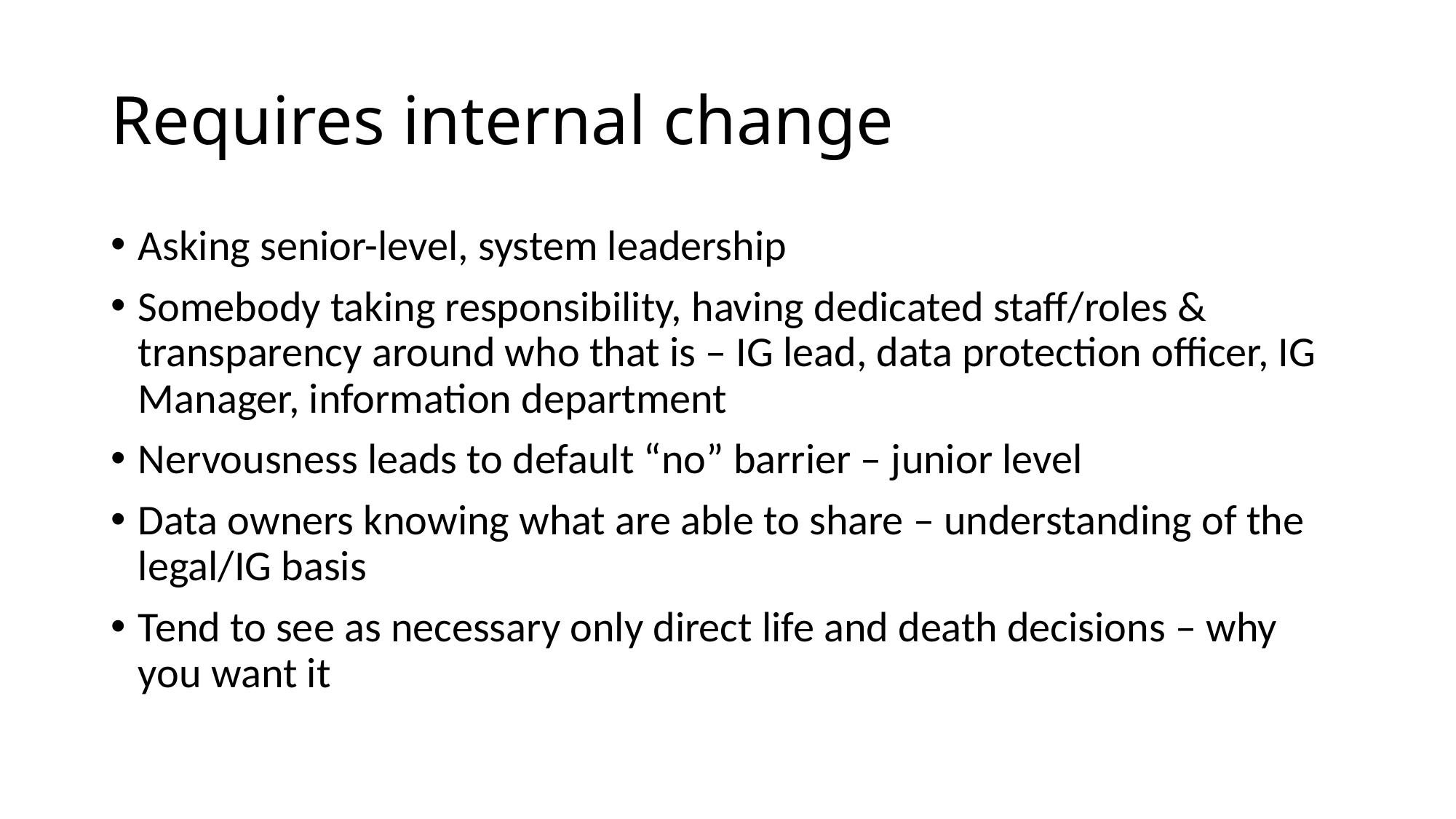

# Requires internal change
Asking senior-level, system leadership
Somebody taking responsibility, having dedicated staff/roles & transparency around who that is – IG lead, data protection officer, IG Manager, information department
Nervousness leads to default “no” barrier – junior level
Data owners knowing what are able to share – understanding of the legal/IG basis
Tend to see as necessary only direct life and death decisions – why you want it

## Slide 36
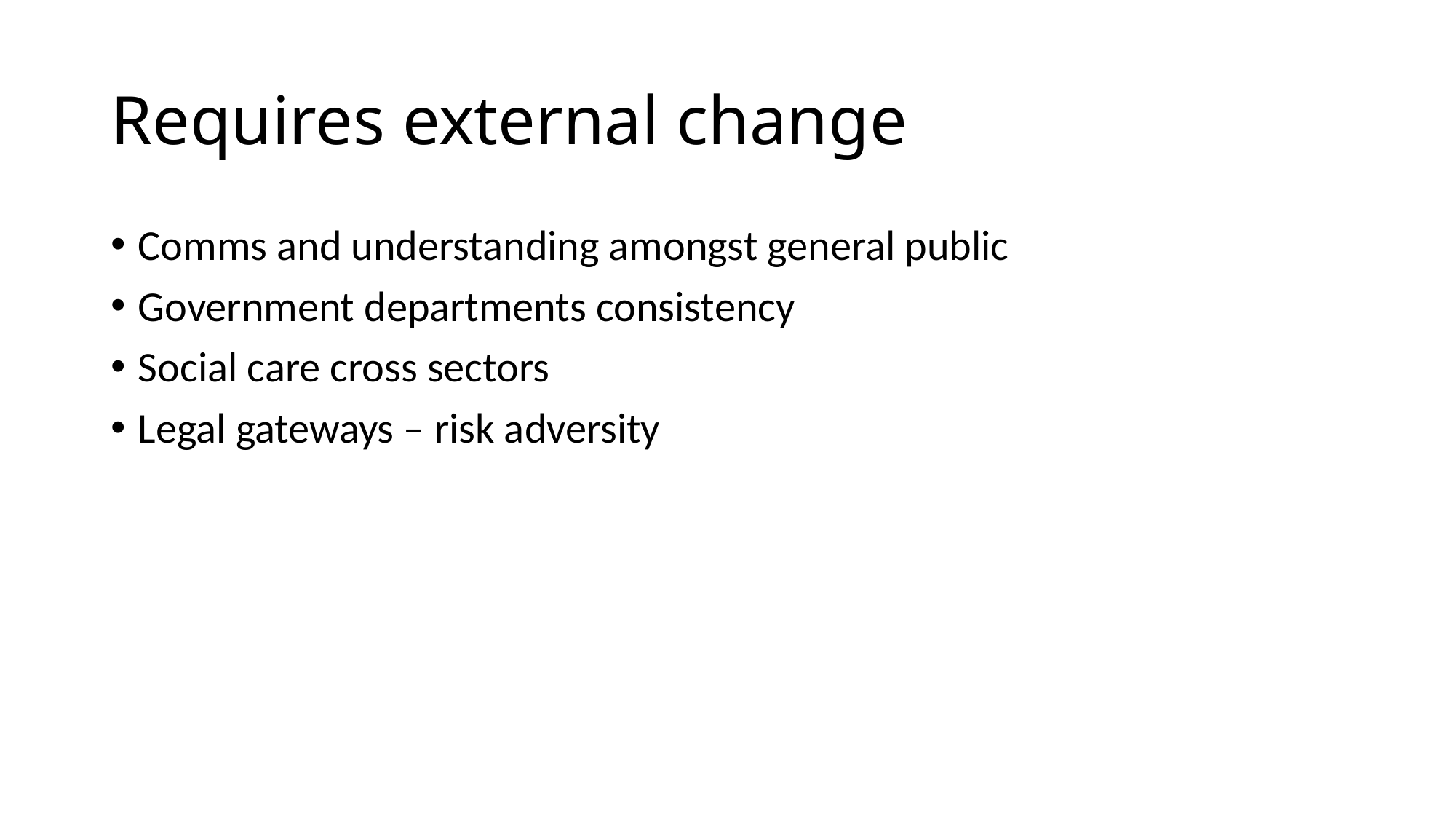

# Requires external change
Comms and understanding amongst general public
Government departments consistency
Social care cross sectors
Legal gateways – risk adversity

## Slide 37
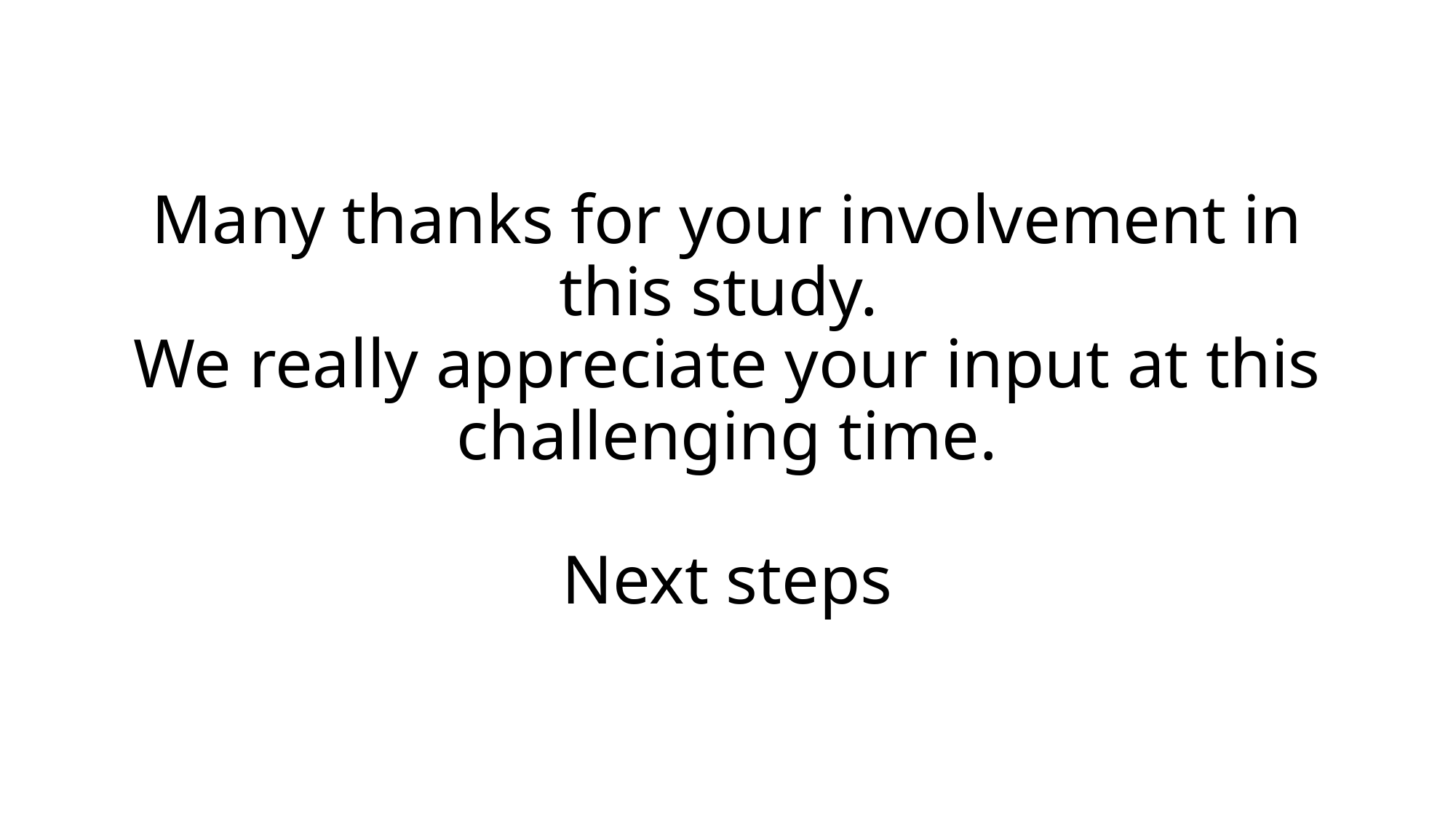

# Many thanks for your involvement in this study. We really appreciate your input at this challenging time. Next steps

## Slide 38
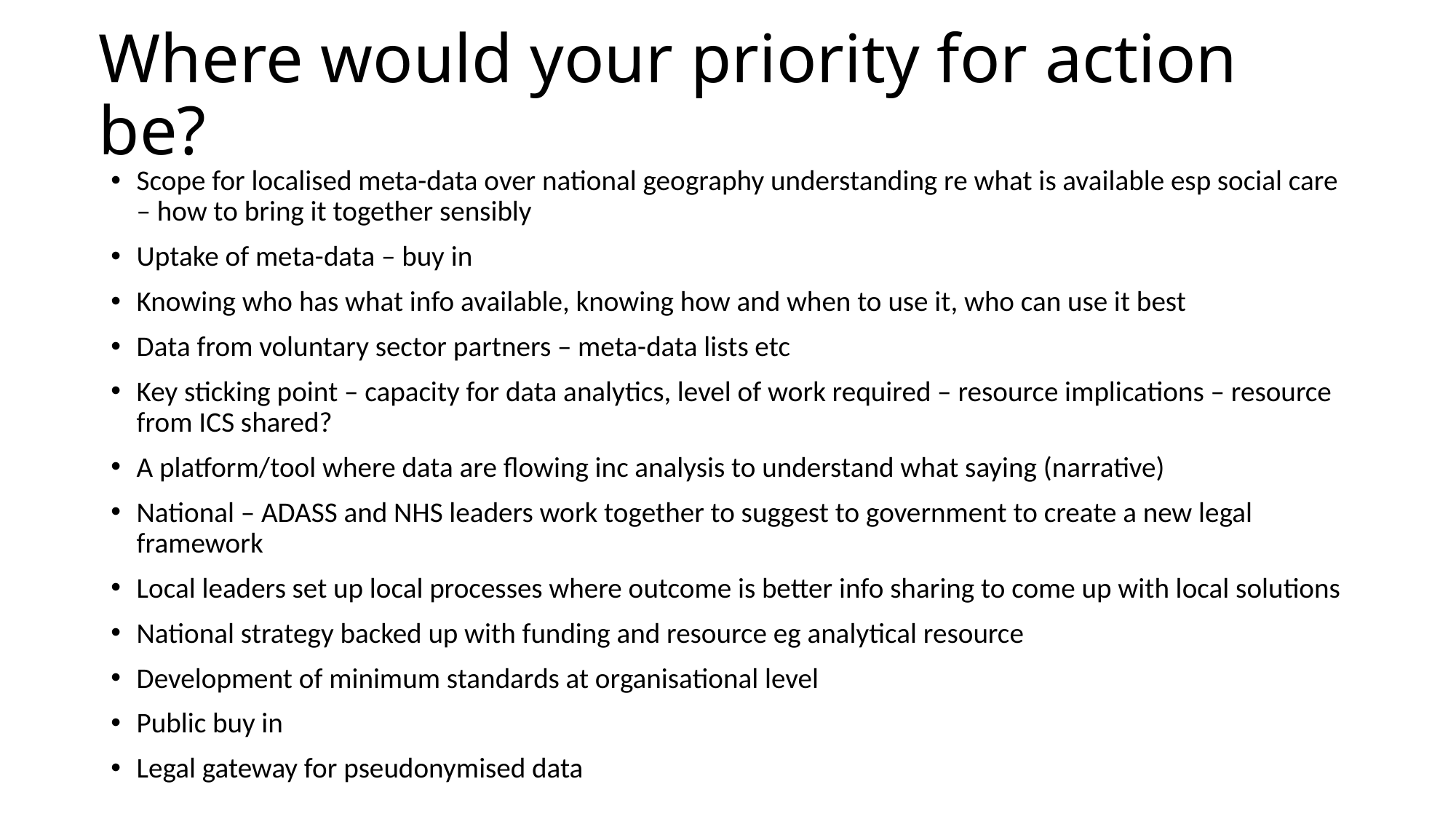

# Where would your priority for action be?
Scope for localised meta-data over national geography understanding re what is available esp social care – how to bring it together sensibly
Uptake of meta-data – buy in
Knowing who has what info available, knowing how and when to use it, who can use it best
Data from voluntary sector partners – meta-data lists etc
Key sticking point – capacity for data analytics, level of work required – resource implications – resource from ICS shared?
A platform/tool where data are flowing inc analysis to understand what saying (narrative)
National – ADASS and NHS leaders work together to suggest to government to create a new legal framework
Local leaders set up local processes where outcome is better info sharing to come up with local solutions
National strategy backed up with funding and resource eg analytical resource
Development of minimum standards at organisational level
Public buy in
Legal gateway for pseudonymised data

## Slide 39
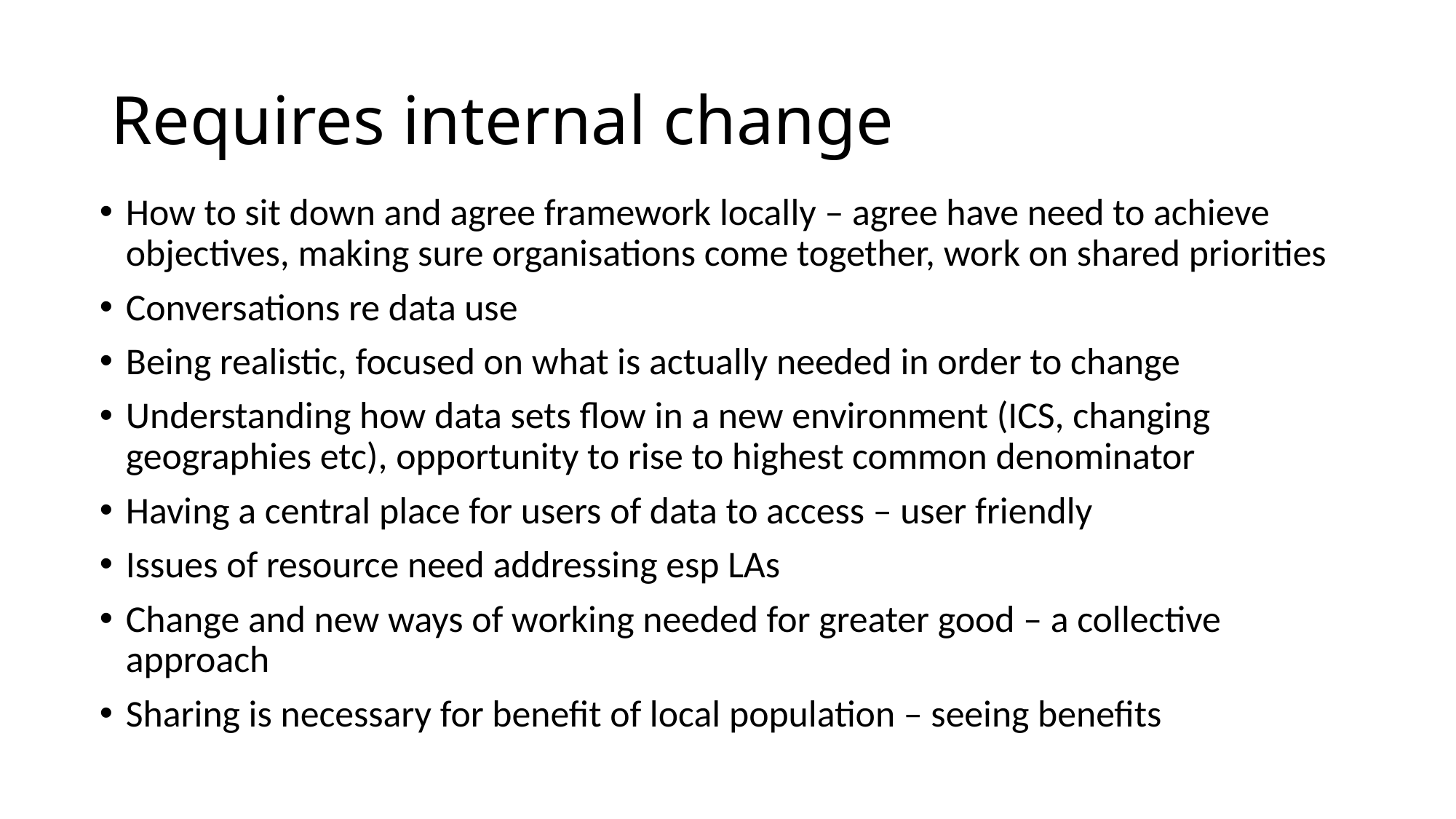

# Requires internal change
How to sit down and agree framework locally – agree have need to achieve objectives, making sure organisations come together, work on shared priorities
Conversations re data use
Being realistic, focused on what is actually needed in order to change
Understanding how data sets flow in a new environment (ICS, changing geographies etc), opportunity to rise to highest common denominator
Having a central place for users of data to access – user friendly
Issues of resource need addressing esp LAs
Change and new ways of working needed for greater good – a collective approach
Sharing is necessary for benefit of local population – seeing benefits

## Slide 40
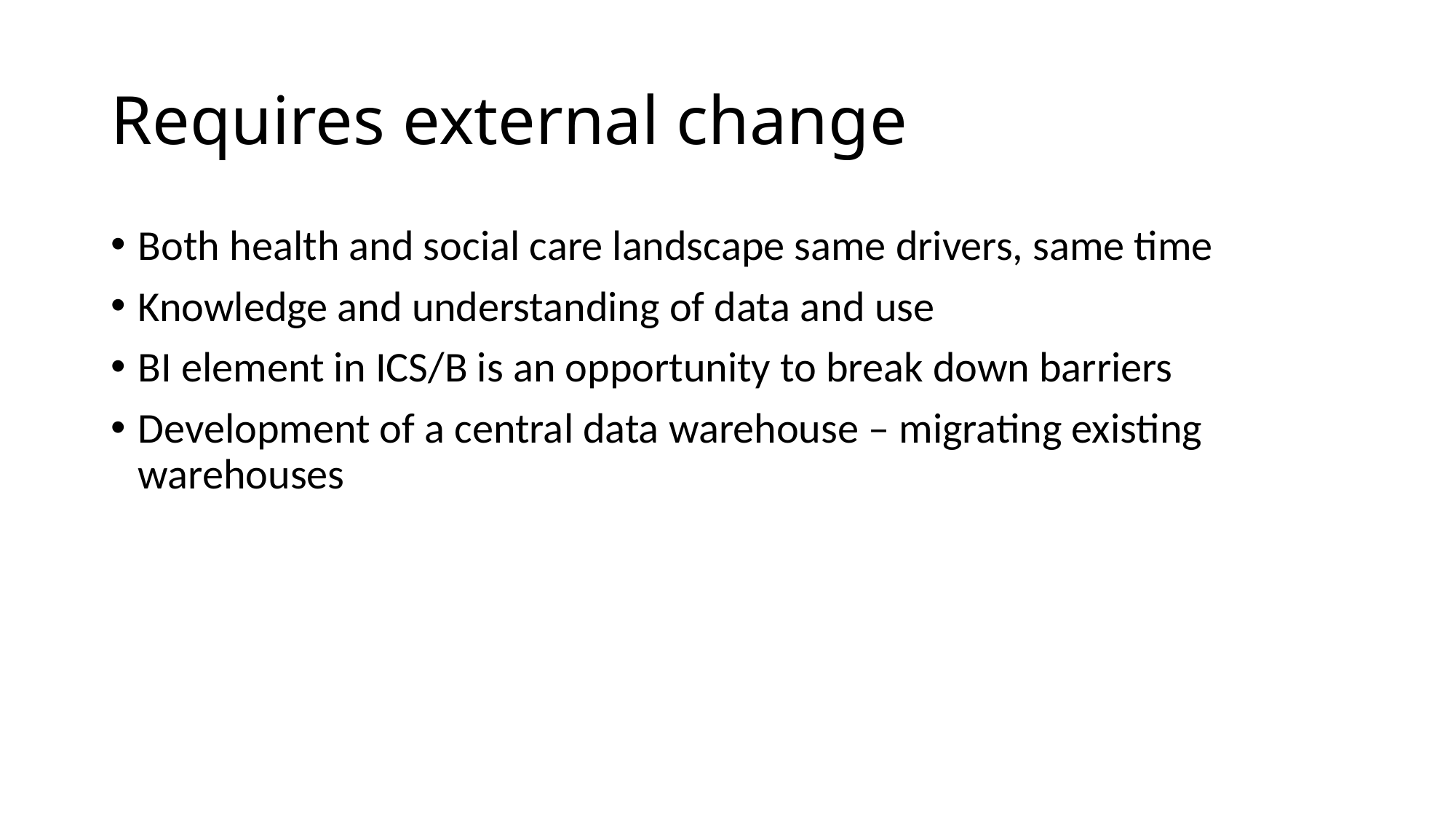

# Requires external change
Both health and social care landscape same drivers, same time
Knowledge and understanding of data and use
BI element in ICS/B is an opportunity to break down barriers
Development of a central data warehouse – migrating existing warehouses

## Slide 41
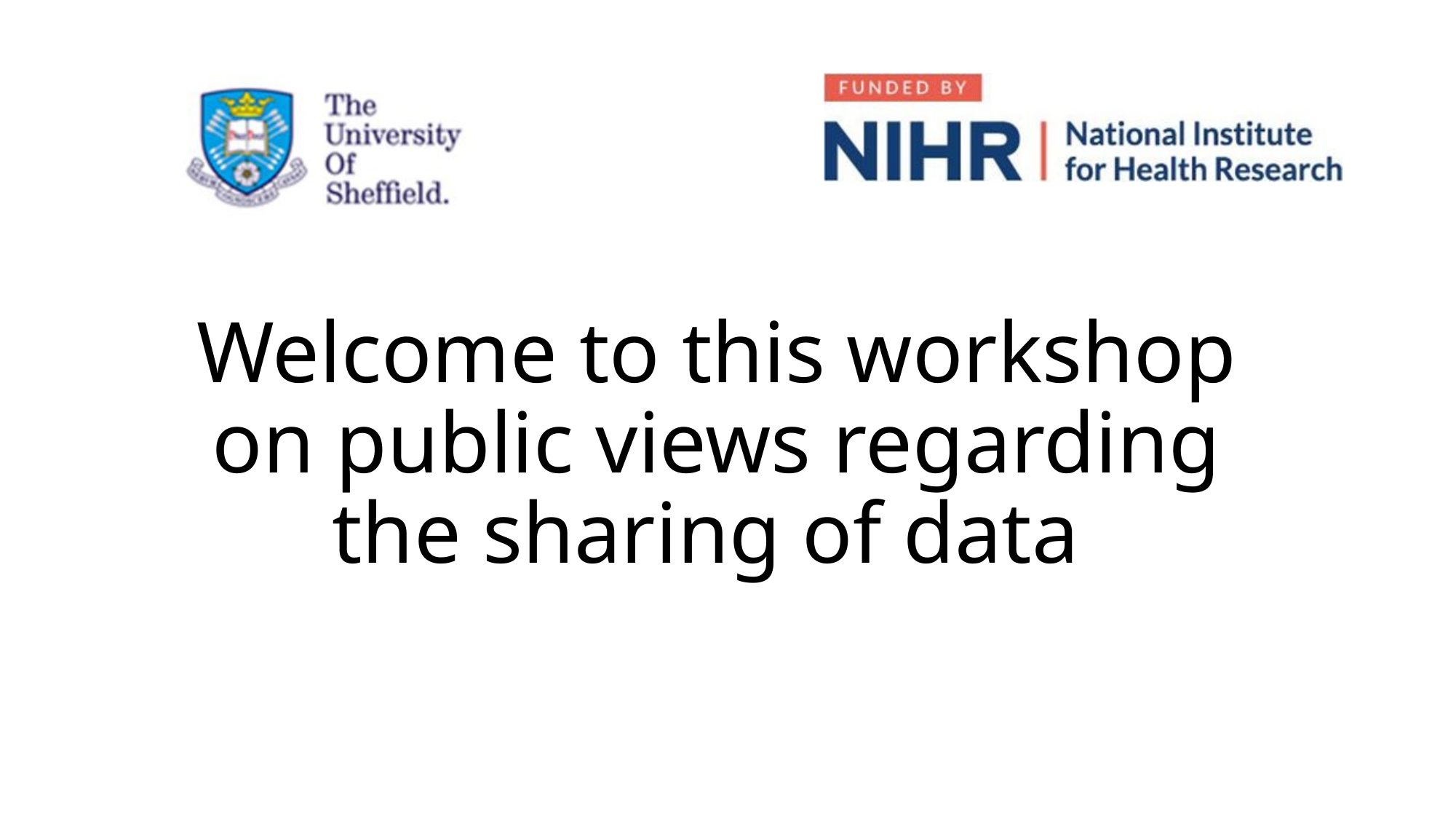

# Welcome to this workshop on public views regarding the sharing of data

## Slide 42
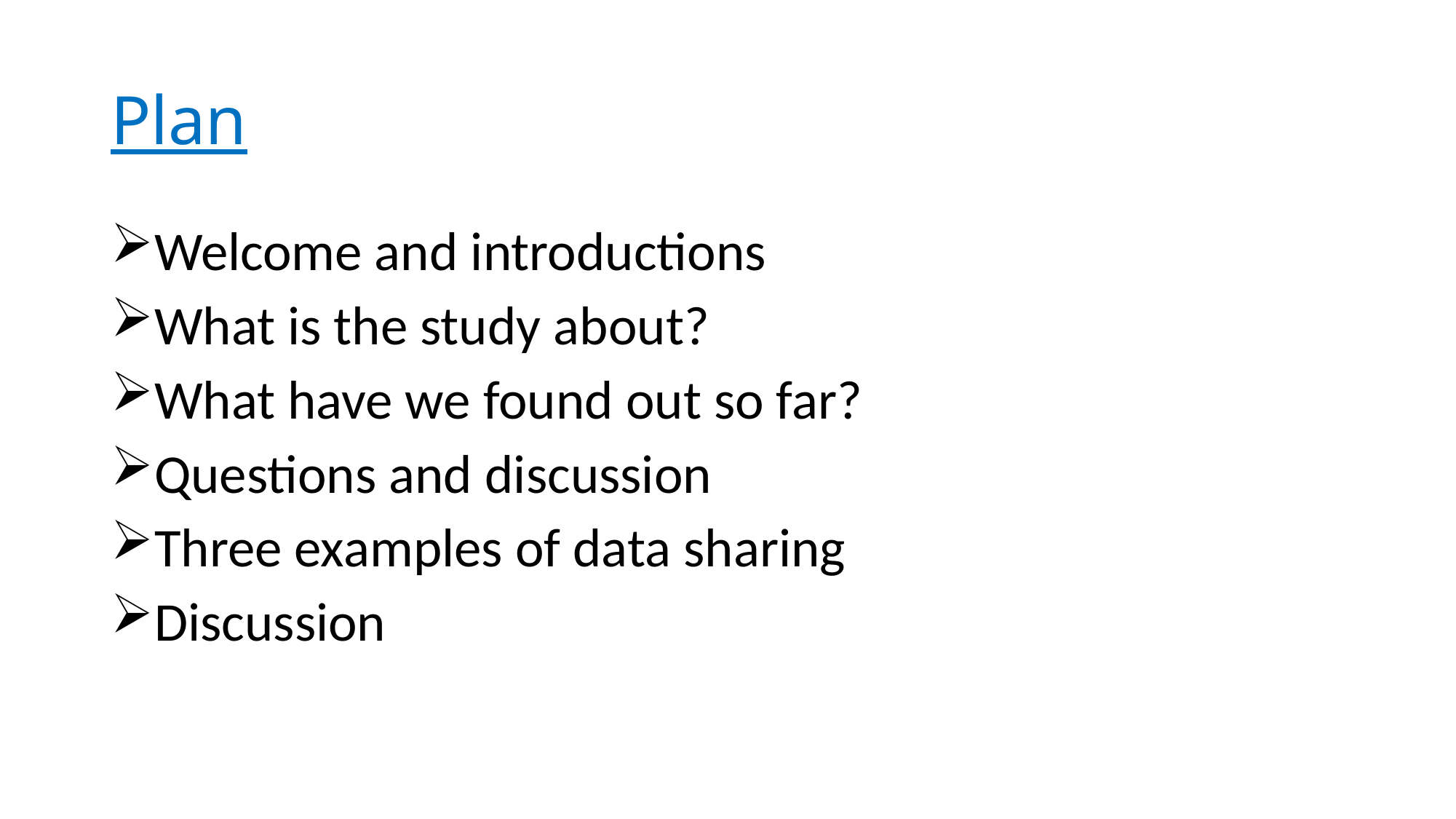

# Plan
Welcome and introductions
What is the study about?
What have we found out so far?
Questions and discussion
Three examples of data sharing
Discussion

## Slide 43
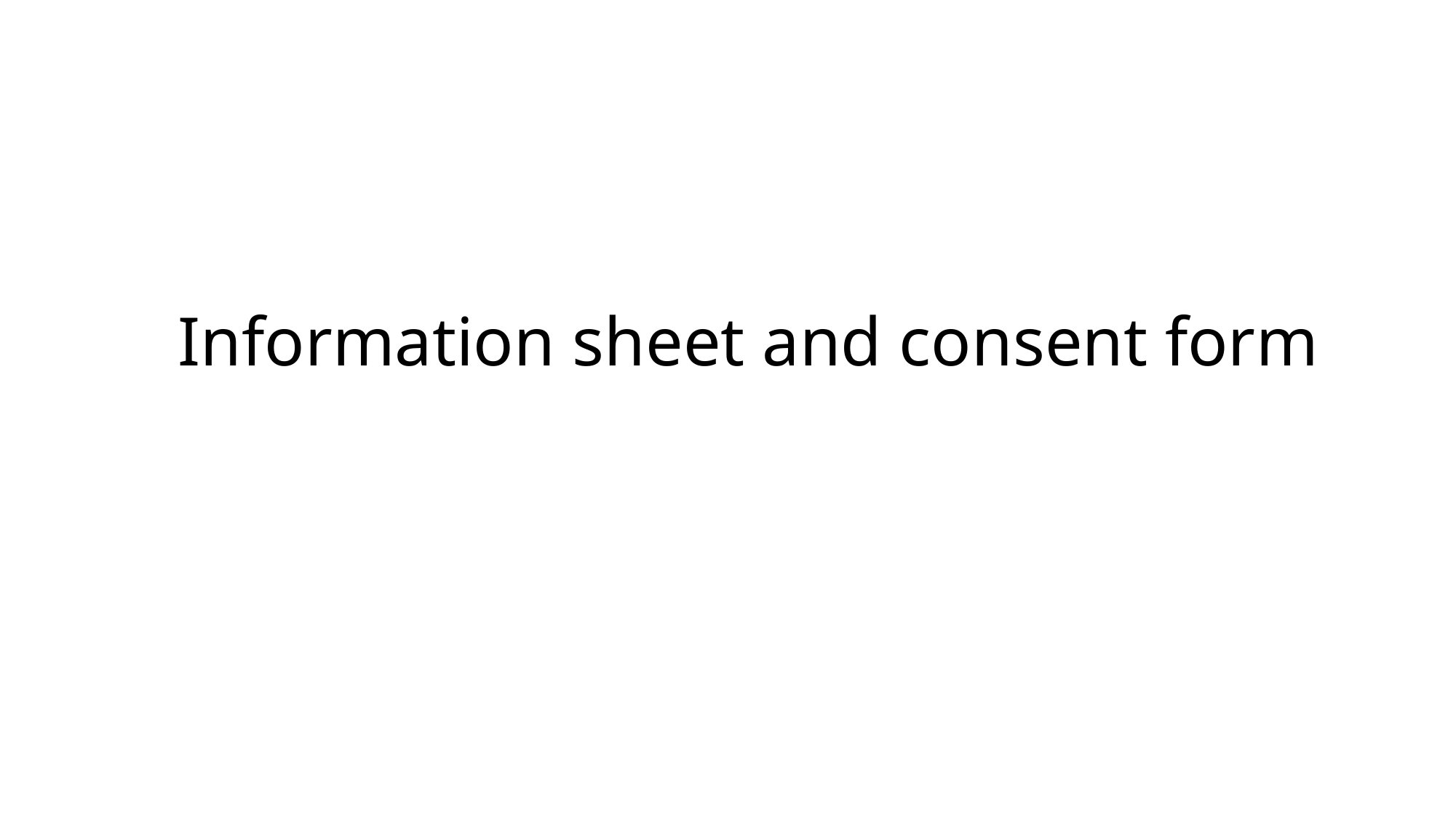

# Information sheet and consent form

## Slide 44
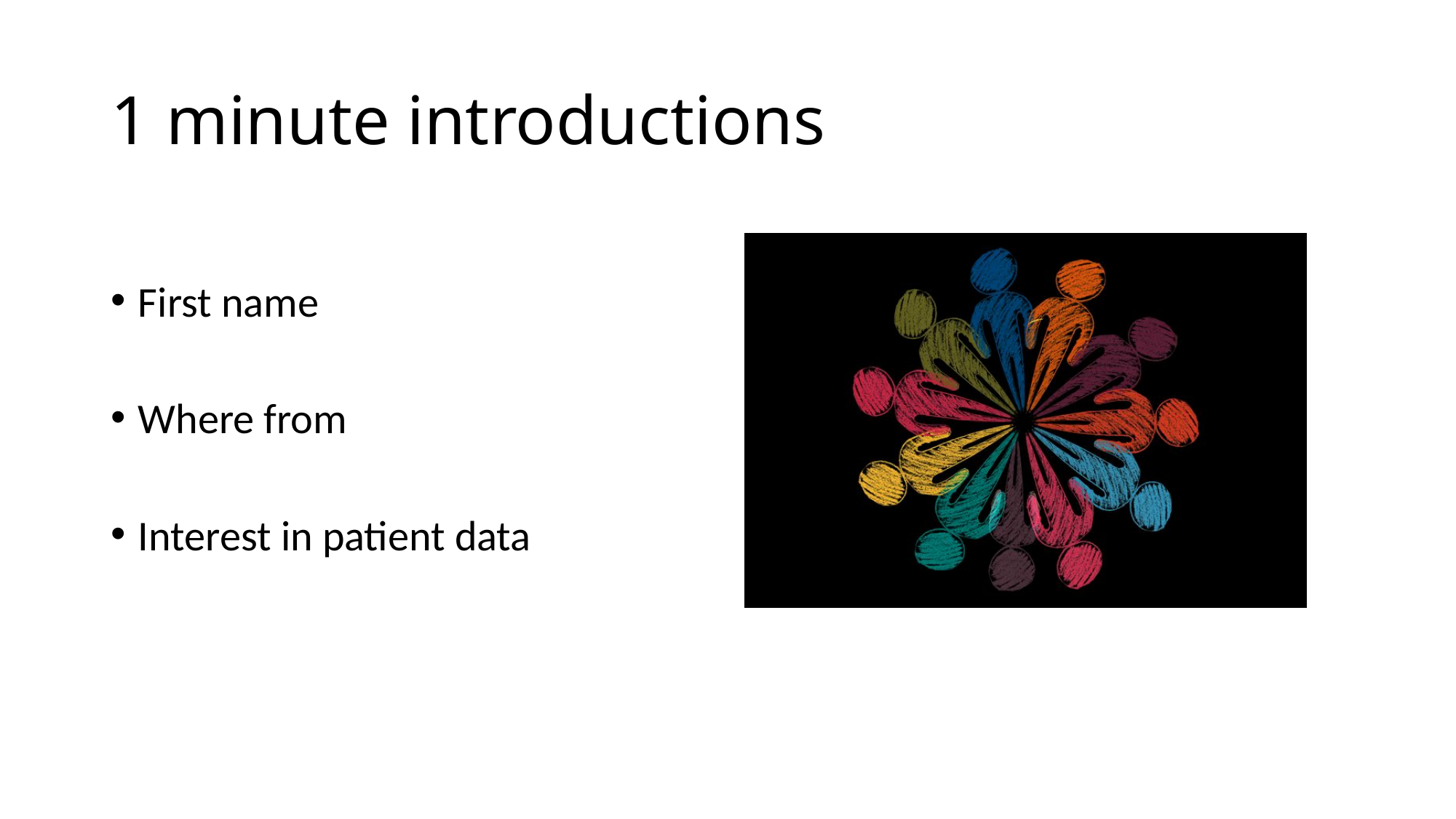

# 1 minute introductions
First name
Where from
Interest in patient data

## Slide 45
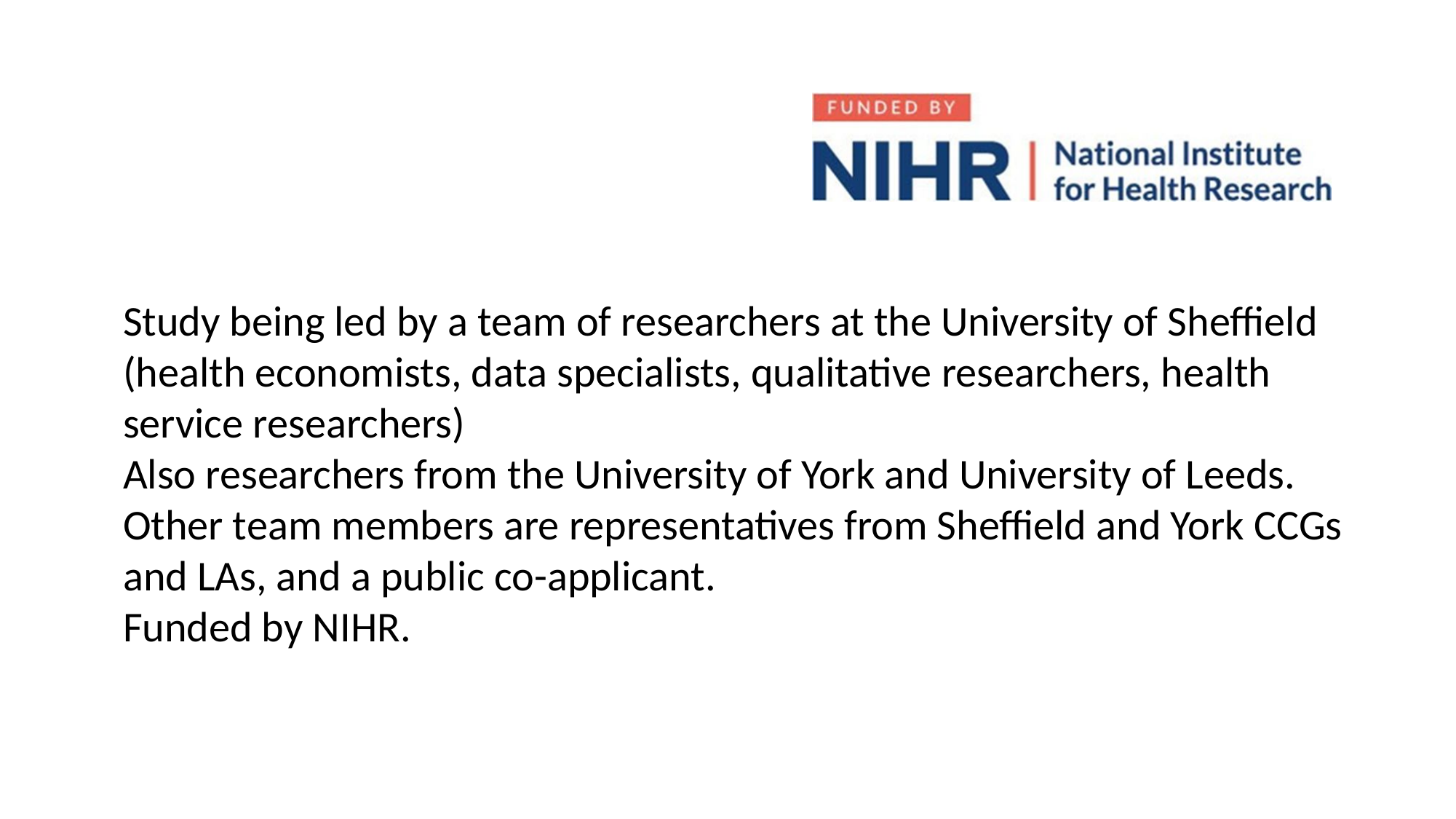

Study being led by a team of researchers at the University of Sheffield (health economists, data specialists, qualitative researchers, health service researchers)
Also researchers from the University of York and University of Leeds.
Other team members are representatives from Sheffield and York CCGs and LAs, and a public co-applicant.
Funded by NIHR.

## Slide 46
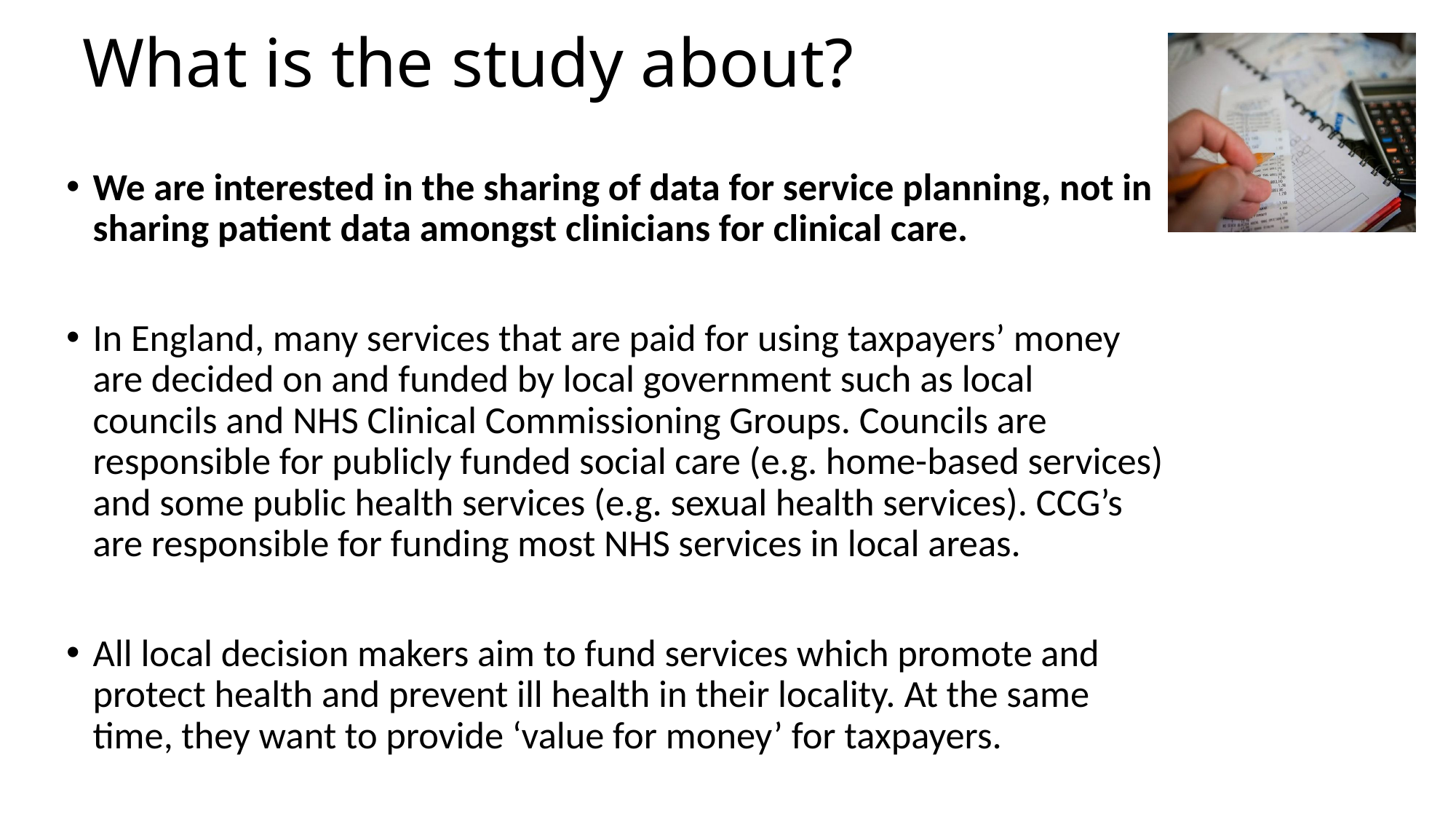

# What is the study about?
We are interested in the sharing of data for service planning, not in sharing patient data amongst clinicians for clinical care.
In England, many services that are paid for using taxpayers’ money are decided on and funded by local government such as local councils and NHS Clinical Commissioning Groups. Councils are responsible for publicly funded social care (e.g. home-based services) and some public health services (e.g. sexual health services). CCG’s are responsible for funding most NHS services in local areas.
All local decision makers aim to fund services which promote and protect health and prevent ill health in their locality. At the same time, they want to provide ‘value for money’ for taxpayers.

## Slide 47
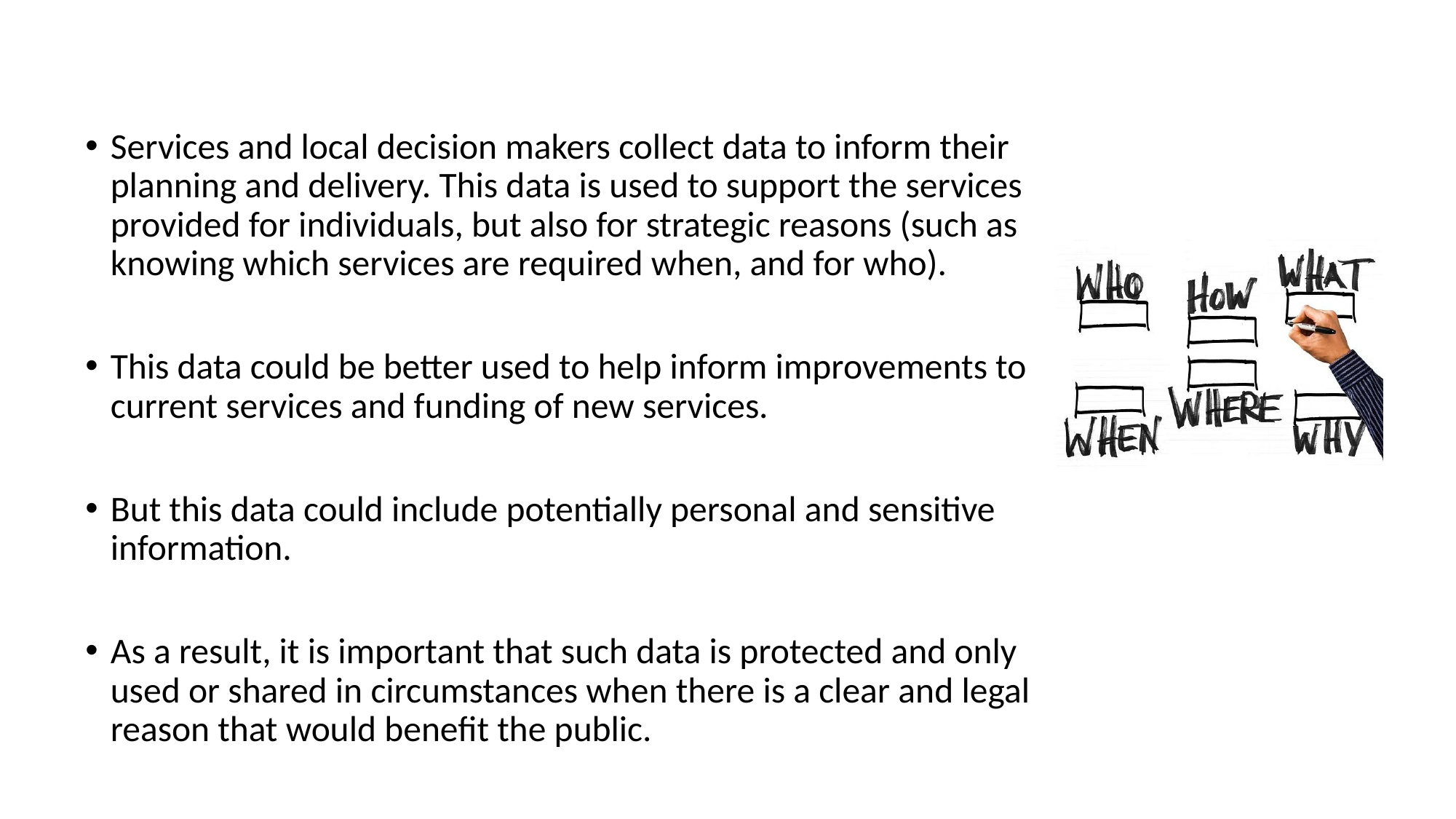

Services and local decision makers collect data to inform their planning and delivery. This data is used to support the services provided for individuals, but also for strategic reasons (such as knowing which services are required when, and for who).
This data could be better used to help inform improvements to current services and funding of new services.
But this data could include potentially personal and sensitive information.
As a result, it is important that such data is protected and only used or shared in circumstances when there is a clear and legal reason that would benefit the public.

## Slide 48
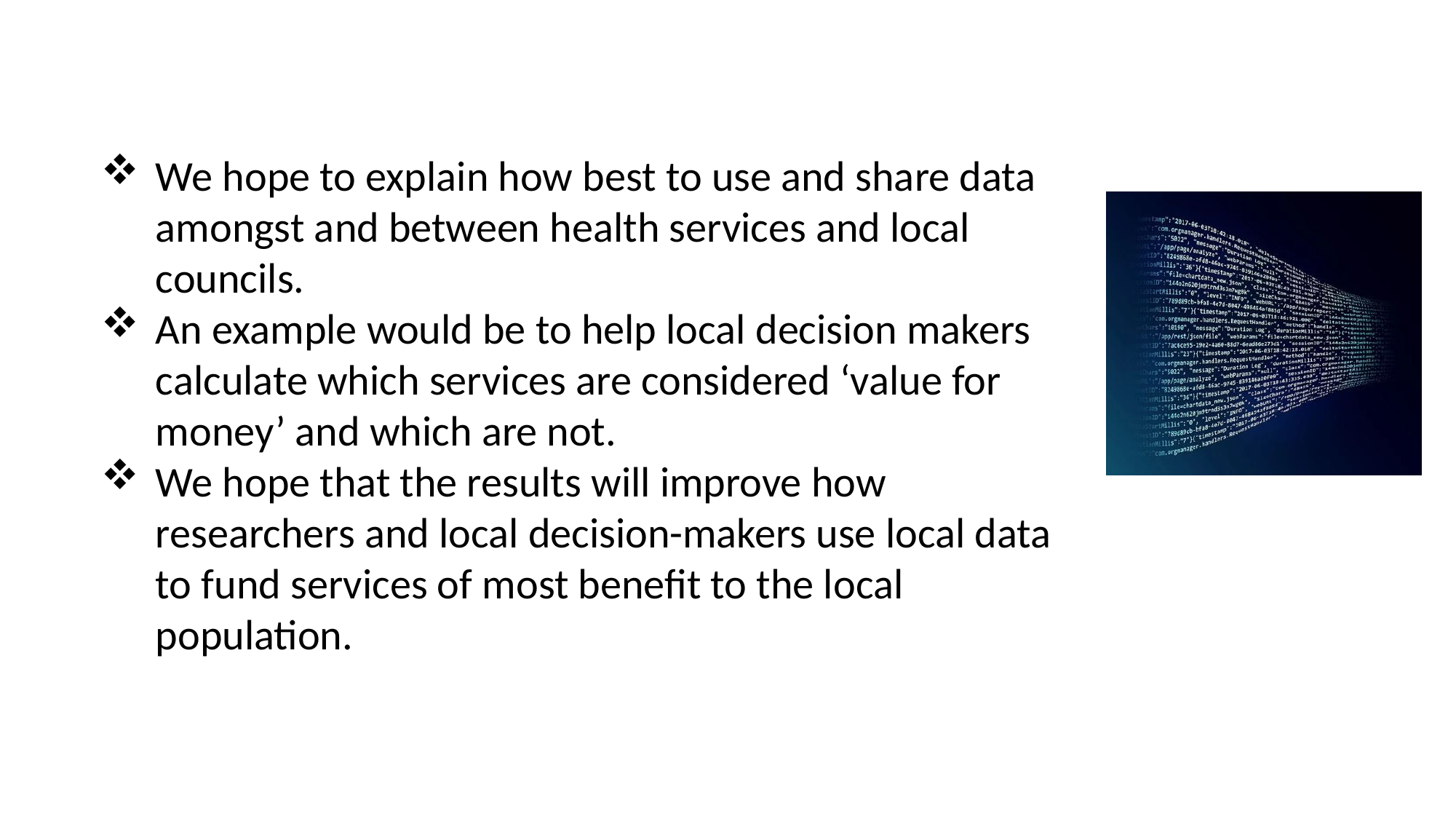

We hope to explain how best to use and share data amongst and between health services and local councils.
An example would be to help local decision makers calculate which services are considered ‘value for money’ and which are not.
We hope that the results will improve how researchers and local decision-makers use local data to fund services of most benefit to the local population.

## Slide 49
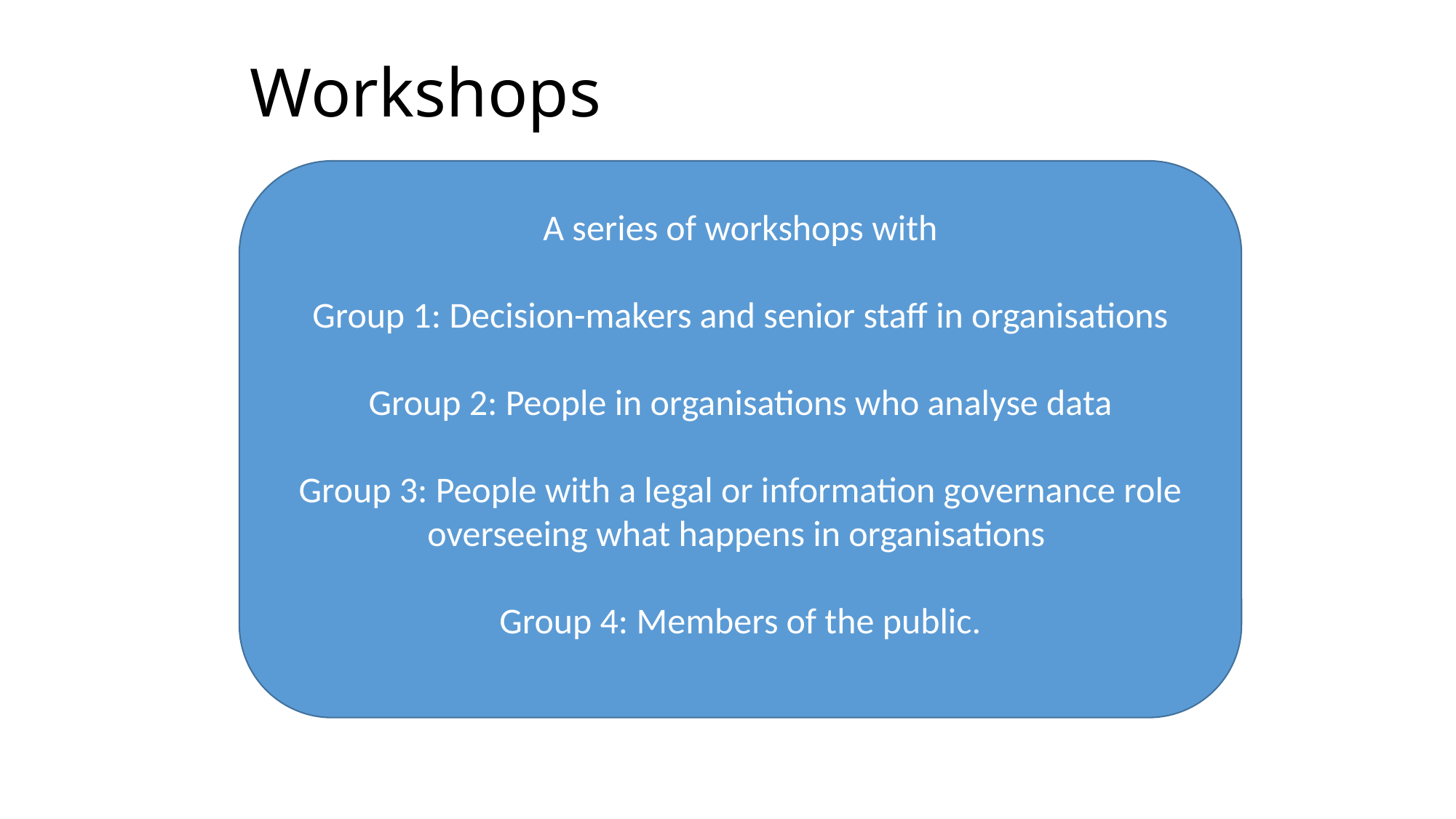

# Workshops
A series of workshops with
Group 1: Decision-makers and senior staff in organisations
Group 2: People in organisations who analyse data
Group 3: People with a legal or information governance role overseeing what happens in organisations
Group 4: Members of the public.

## Slide 50
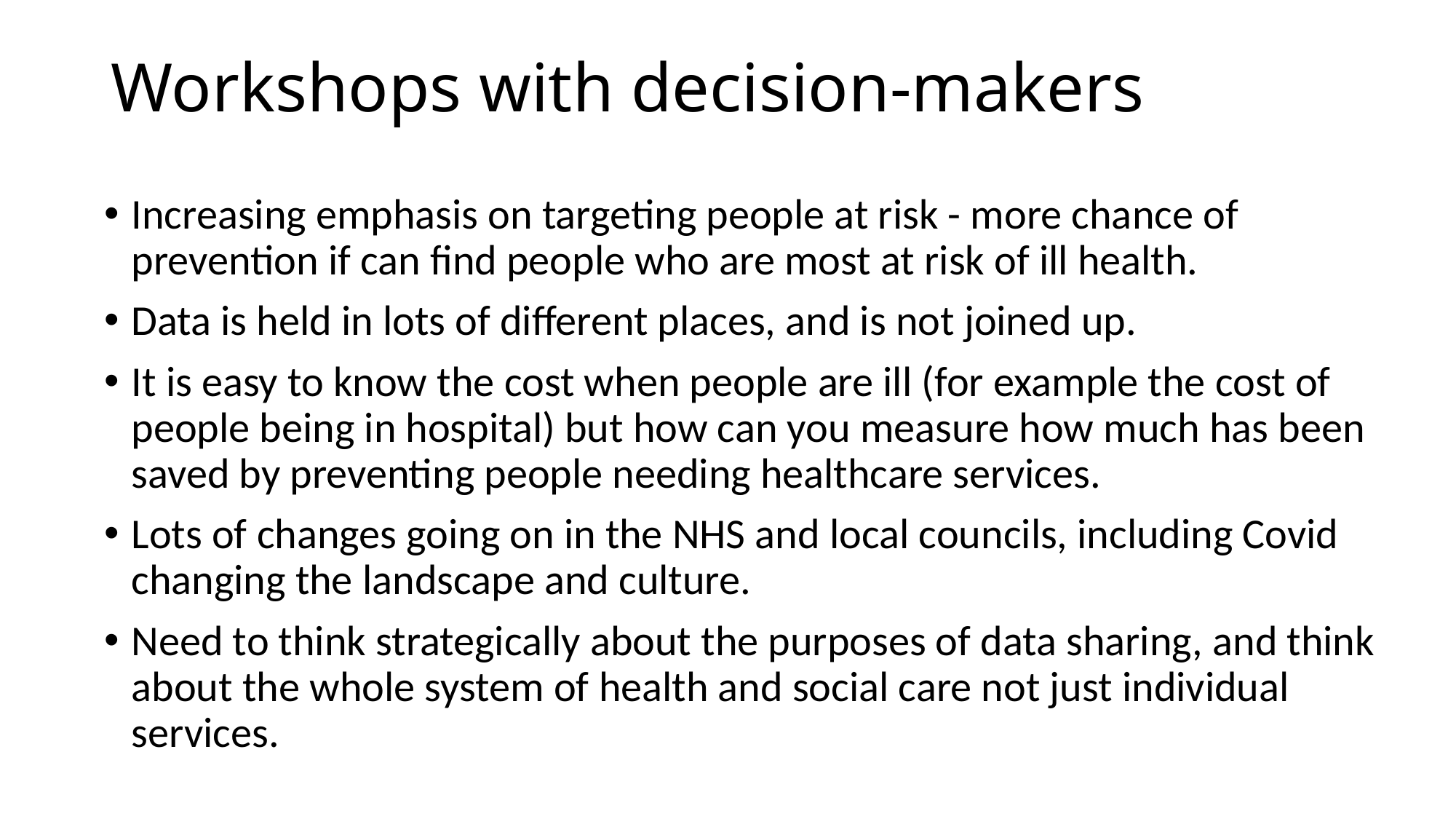

# Workshops with decision-makers
Increasing emphasis on targeting people at risk - more chance of prevention if can find people who are most at risk of ill health.
Data is held in lots of different places, and is not joined up.
It is easy to know the cost when people are ill (for example the cost of people being in hospital) but how can you measure how much has been saved by preventing people needing healthcare services.
Lots of changes going on in the NHS and local councils, including Covid changing the landscape and culture.
Need to think strategically about the purposes of data sharing, and think about the whole system of health and social care not just individual services.

## Slide 51
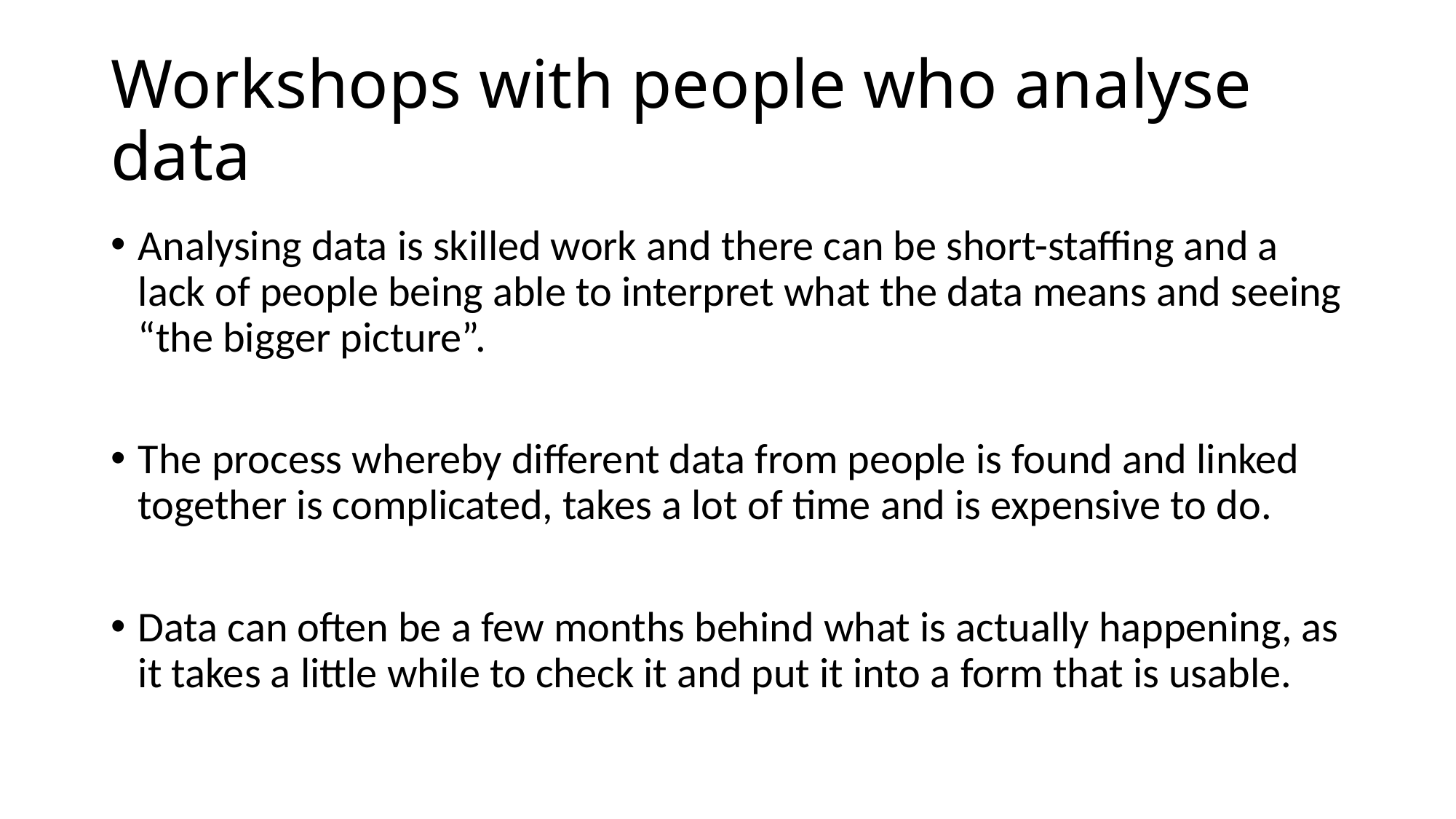

# Workshops with people who analyse data
Analysing data is skilled work and there can be short-staffing and a lack of people being able to interpret what the data means and seeing “the bigger picture”.
The process whereby different data from people is found and linked together is complicated, takes a lot of time and is expensive to do.
Data can often be a few months behind what is actually happening, as it takes a little while to check it and put it into a form that is usable.

## Slide 52
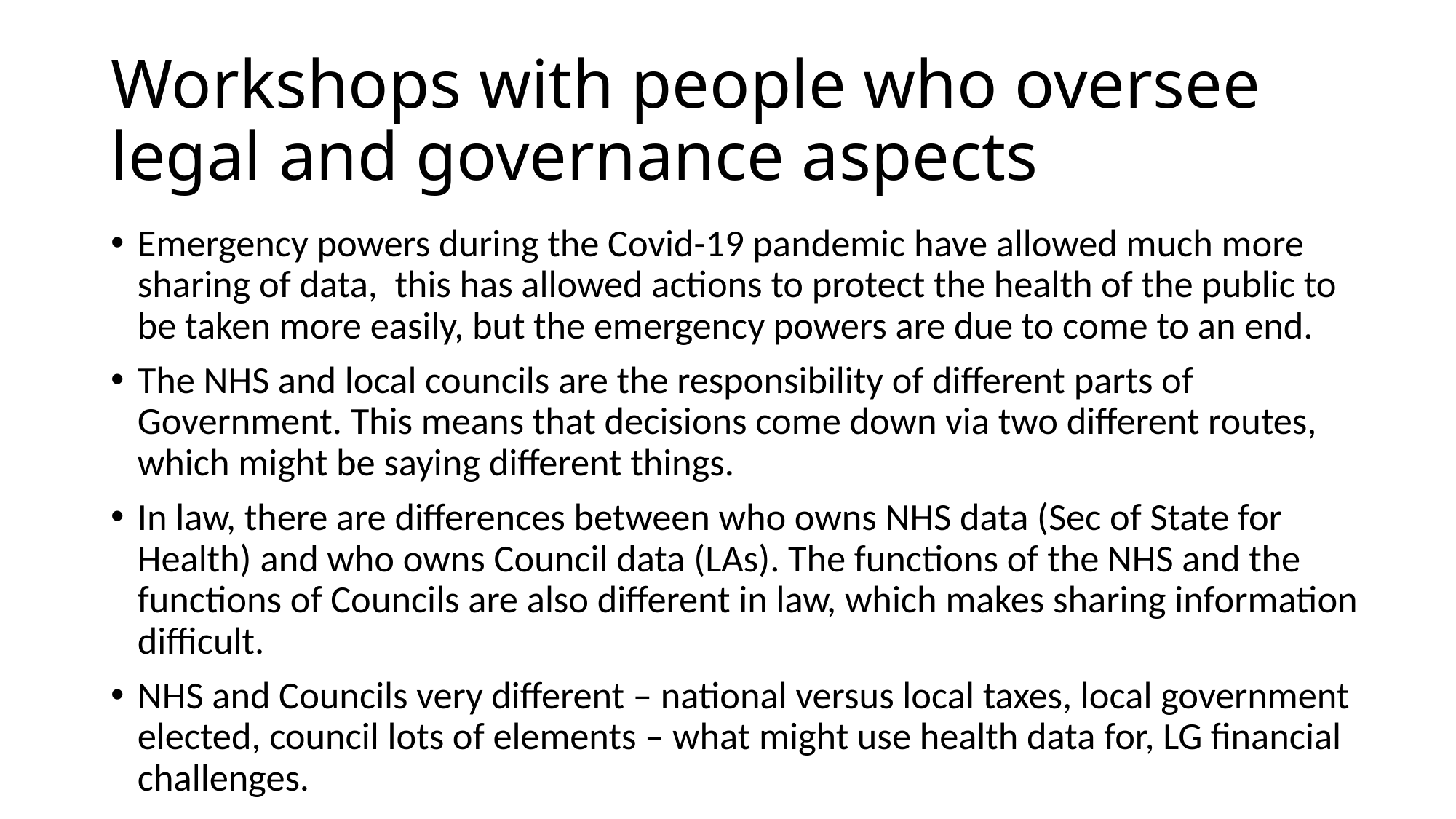

# Workshops with people who oversee legal and governance aspects
Emergency powers during the Covid-19 pandemic have allowed much more sharing of data, this has allowed actions to protect the health of the public to be taken more easily, but the emergency powers are due to come to an end.
The NHS and local councils are the responsibility of different parts of Government. This means that decisions come down via two different routes, which might be saying different things.
In law, there are differences between who owns NHS data (Sec of State for Health) and who owns Council data (LAs). The functions of the NHS and the functions of Councils are also different in law, which makes sharing information difficult.
NHS and Councils very different – national versus local taxes, local government elected, council lots of elements – what might use health data for, LG financial challenges.

## Slide 53
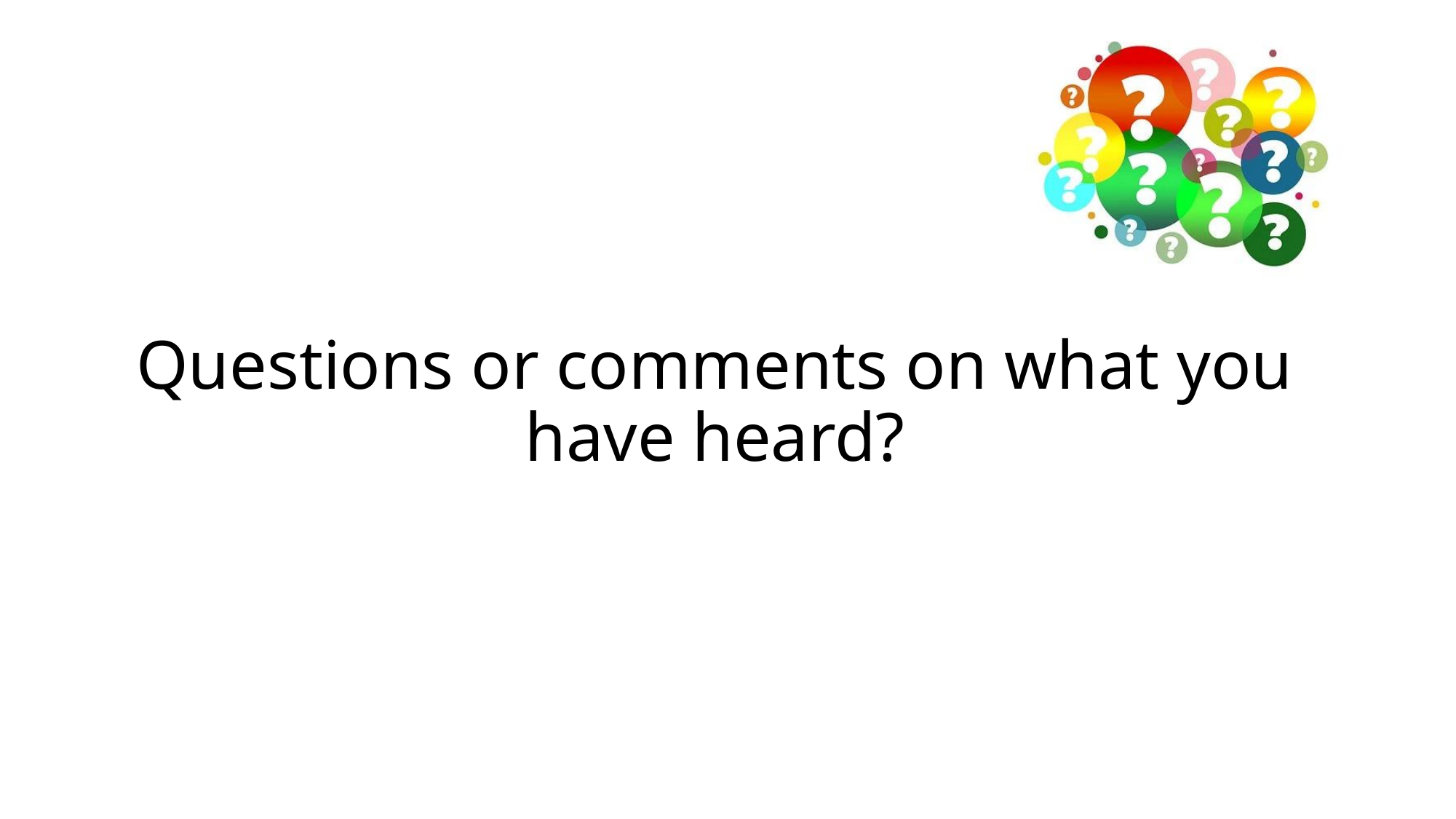

# Questions or comments on what you have heard?

## Slide 54
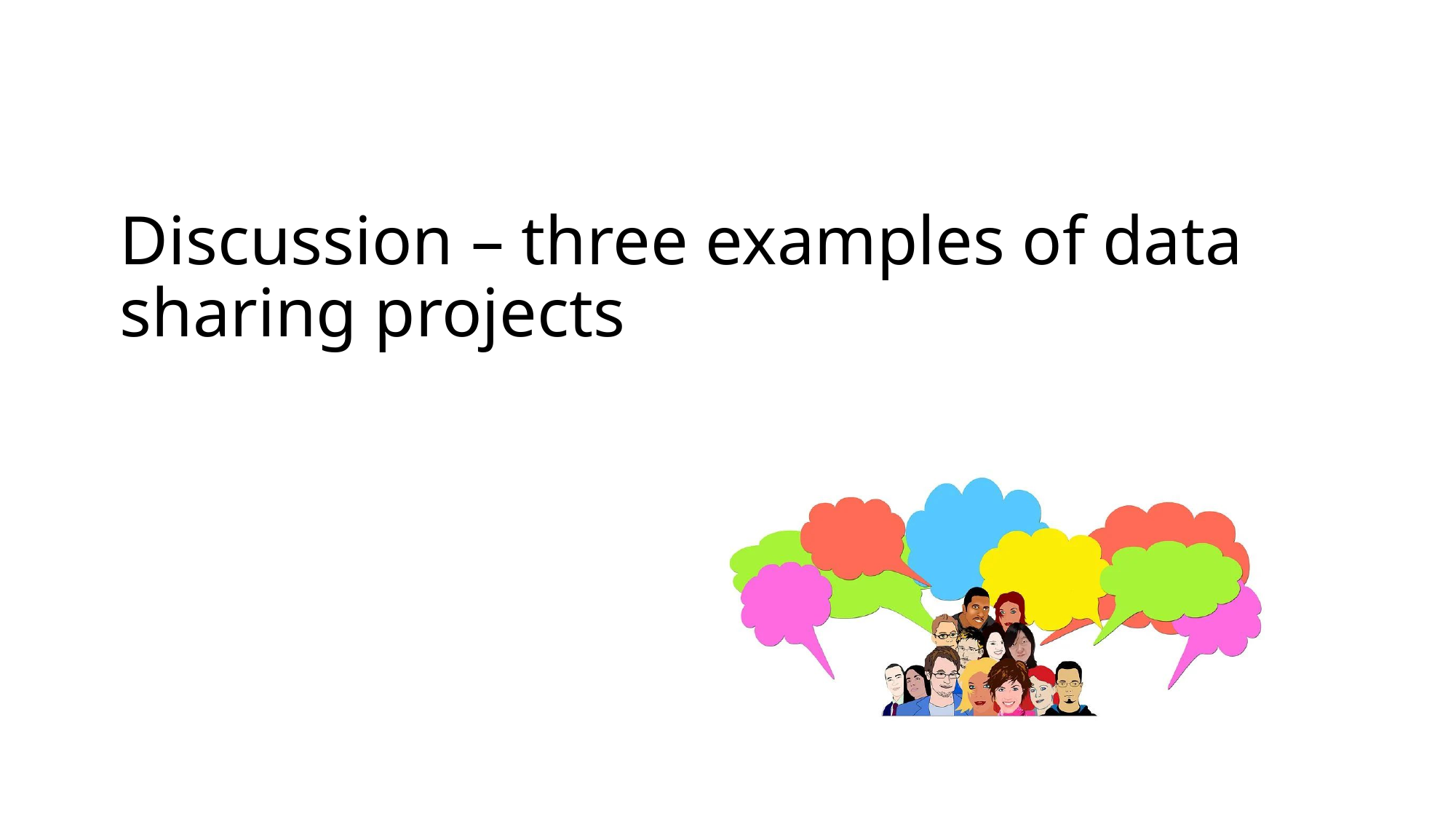

# Discussion – three examples of data sharing projects

## Slide 55
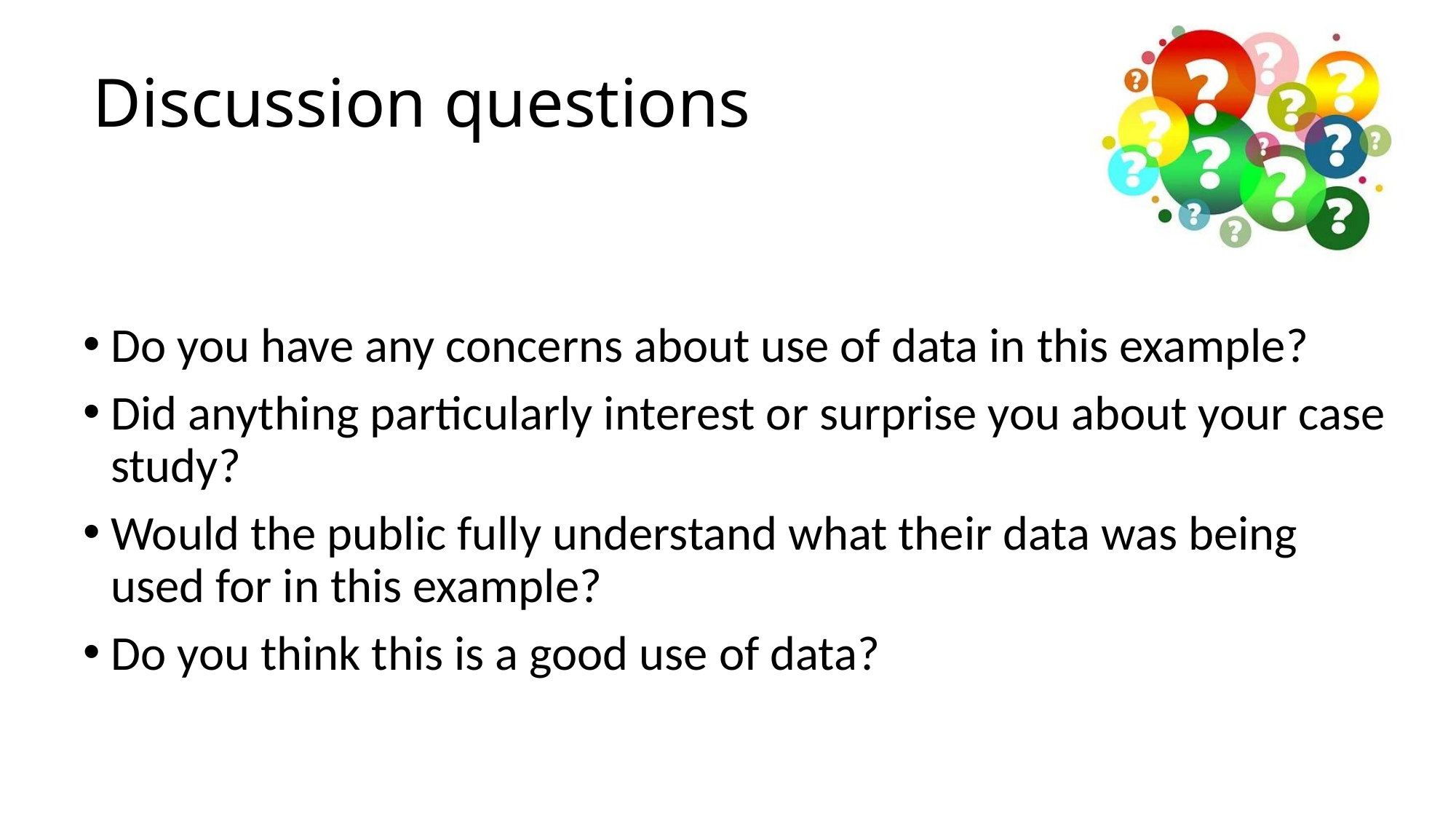

# Discussion questions
Do you have any concerns about use of data in this example?
Did anything particularly interest or surprise you about your case study?
Would the public fully understand what their data was being used for in this example?
Do you think this is a good use of data?

## Slide 56
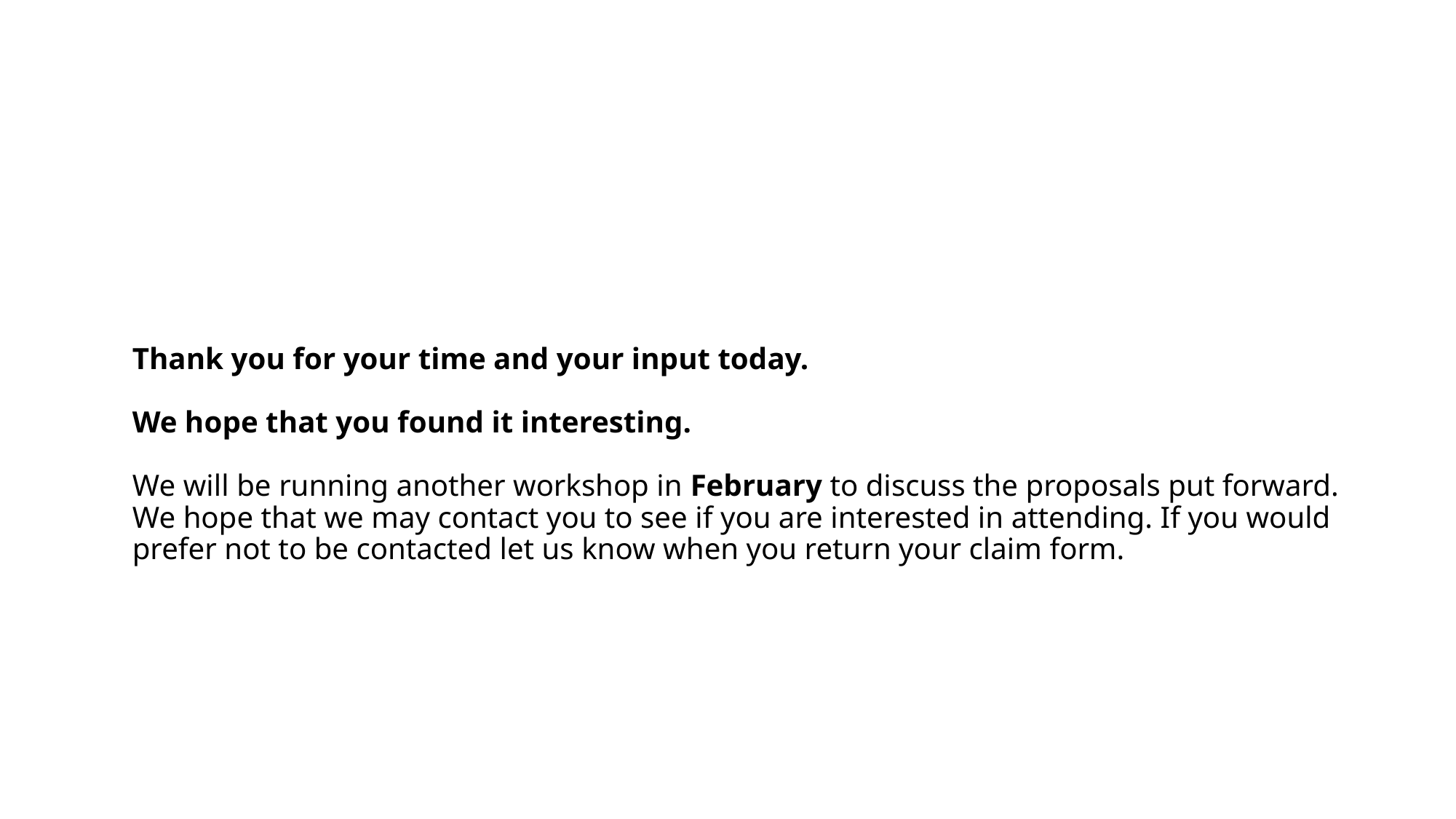

# Thank you for your time and your input today. We hope that you found it interesting. We will be running another workshop in February to discuss the proposals put forward. We hope that we may contact you to see if you are interested in attending. If you would prefer not to be contacted let us know when you return your claim form.

## Slide 57
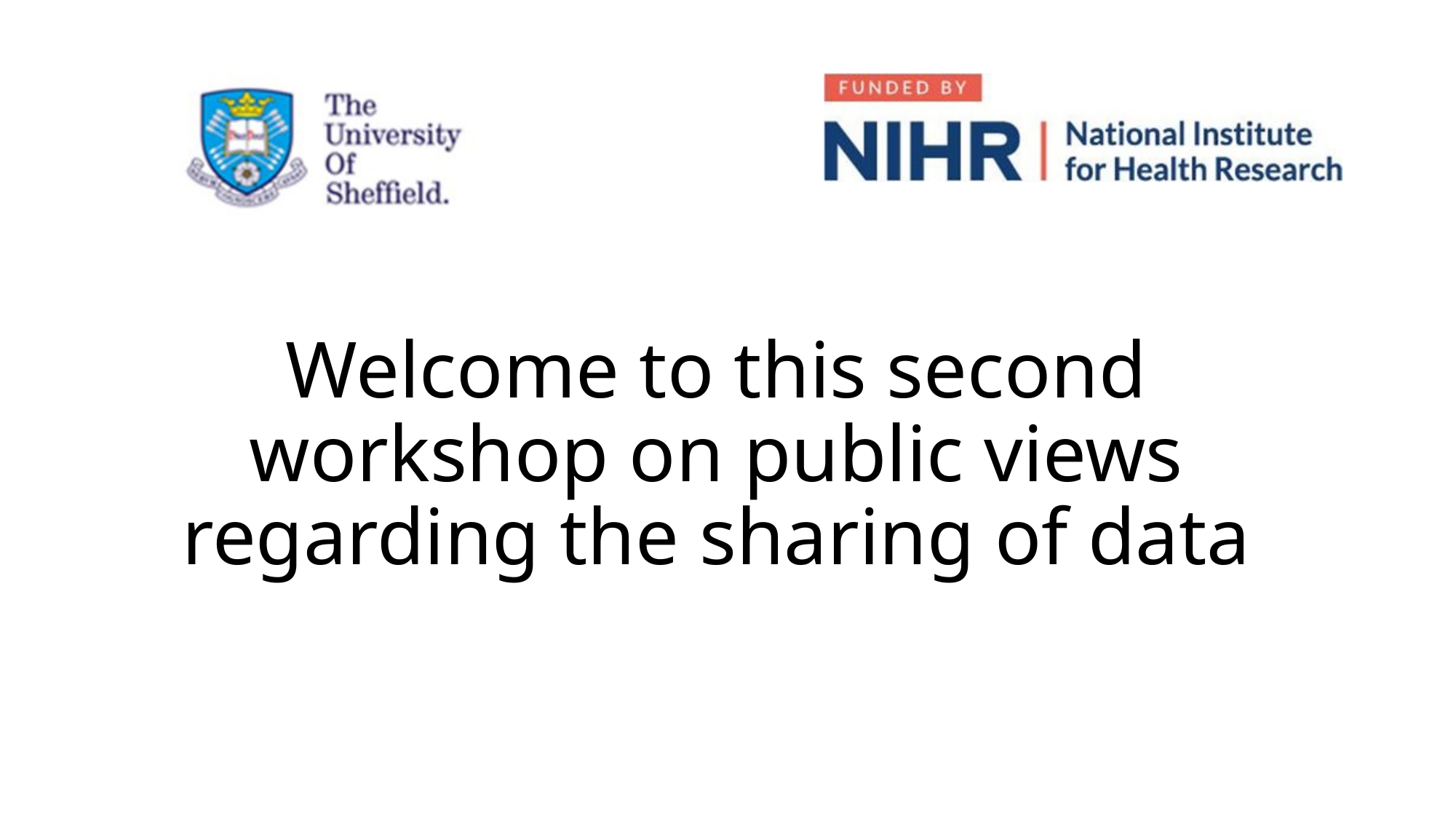

# Welcome to this second workshop on public views regarding the sharing of data

## Slide 58
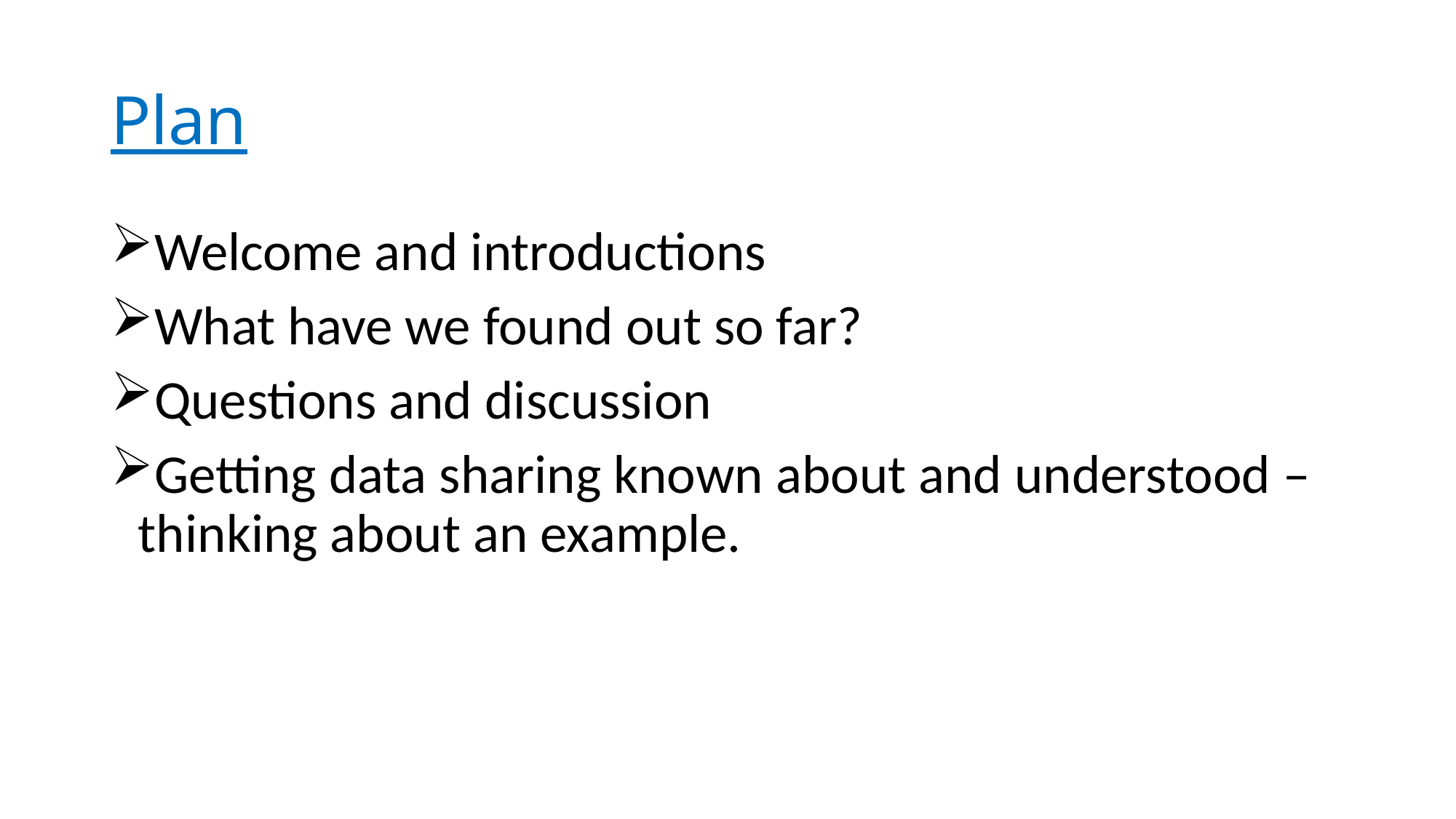

# Plan
Welcome and introductions
What have we found out so far?
Questions and discussion
Getting data sharing known about and understood – thinking about an example.

## Slide 59
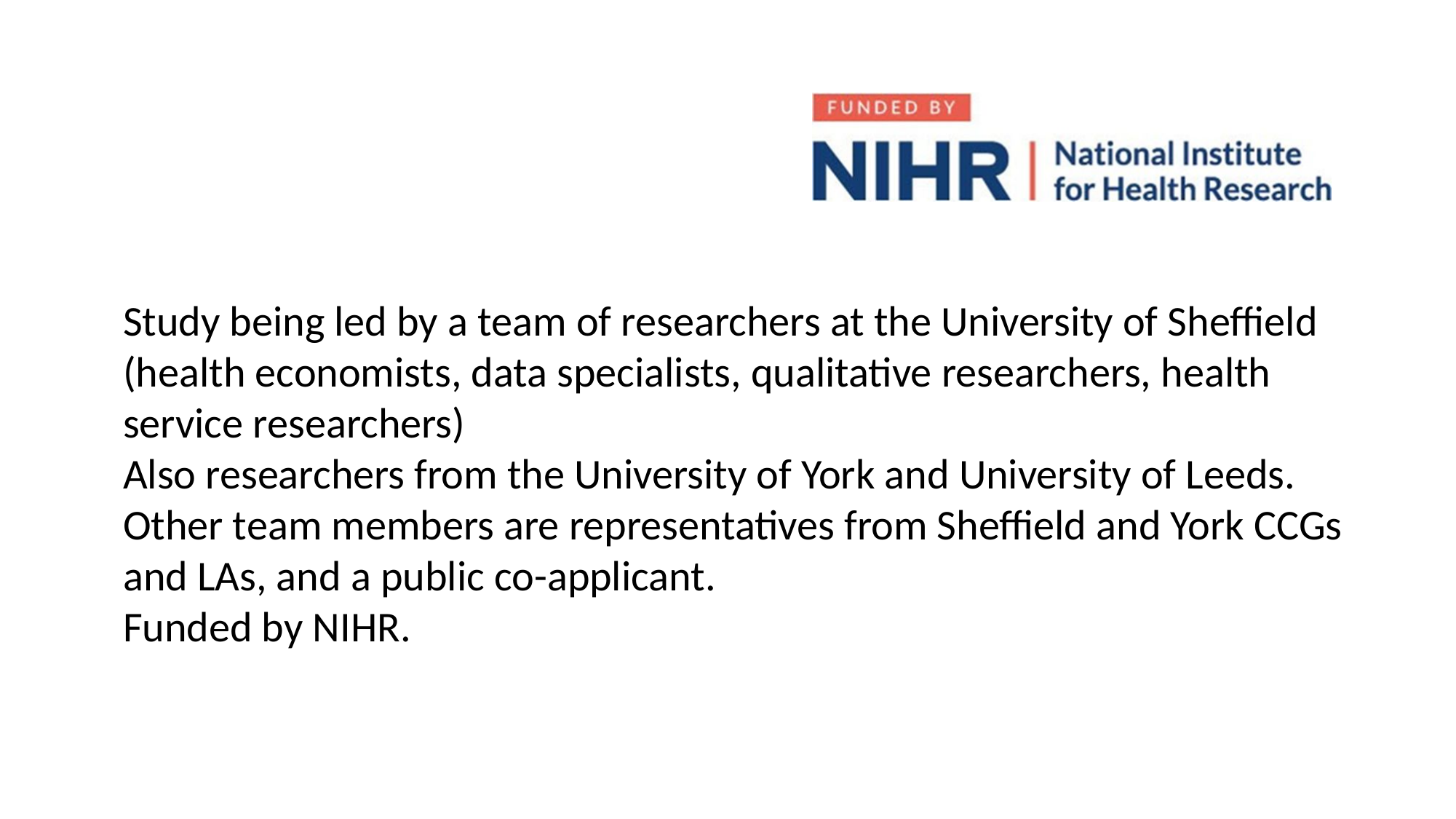

Study being led by a team of researchers at the University of Sheffield (health economists, data specialists, qualitative researchers, health service researchers)
Also researchers from the University of York and University of Leeds.
Other team members are representatives from Sheffield and York CCGs and LAs, and a public co-applicant.
Funded by NIHR.

## Slide 60
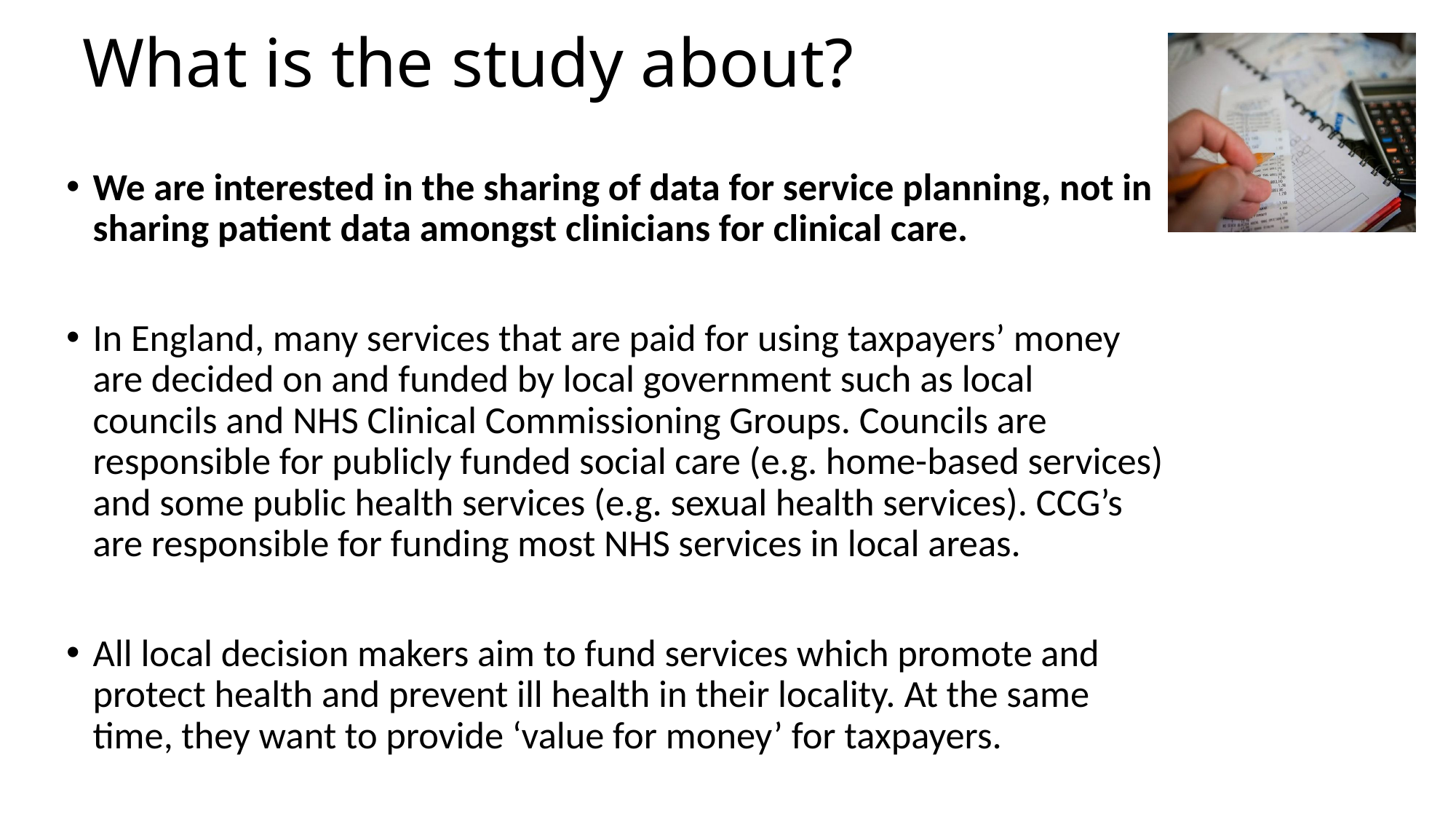

# What is the study about?
We are interested in the sharing of data for service planning, not in sharing patient data amongst clinicians for clinical care.
In England, many services that are paid for using taxpayers’ money are decided on and funded by local government such as local councils and NHS Clinical Commissioning Groups. Councils are responsible for publicly funded social care (e.g. home-based services) and some public health services (e.g. sexual health services). CCG’s are responsible for funding most NHS services in local areas.
All local decision makers aim to fund services which promote and protect health and prevent ill health in their locality. At the same time, they want to provide ‘value for money’ for taxpayers.

## Slide 61
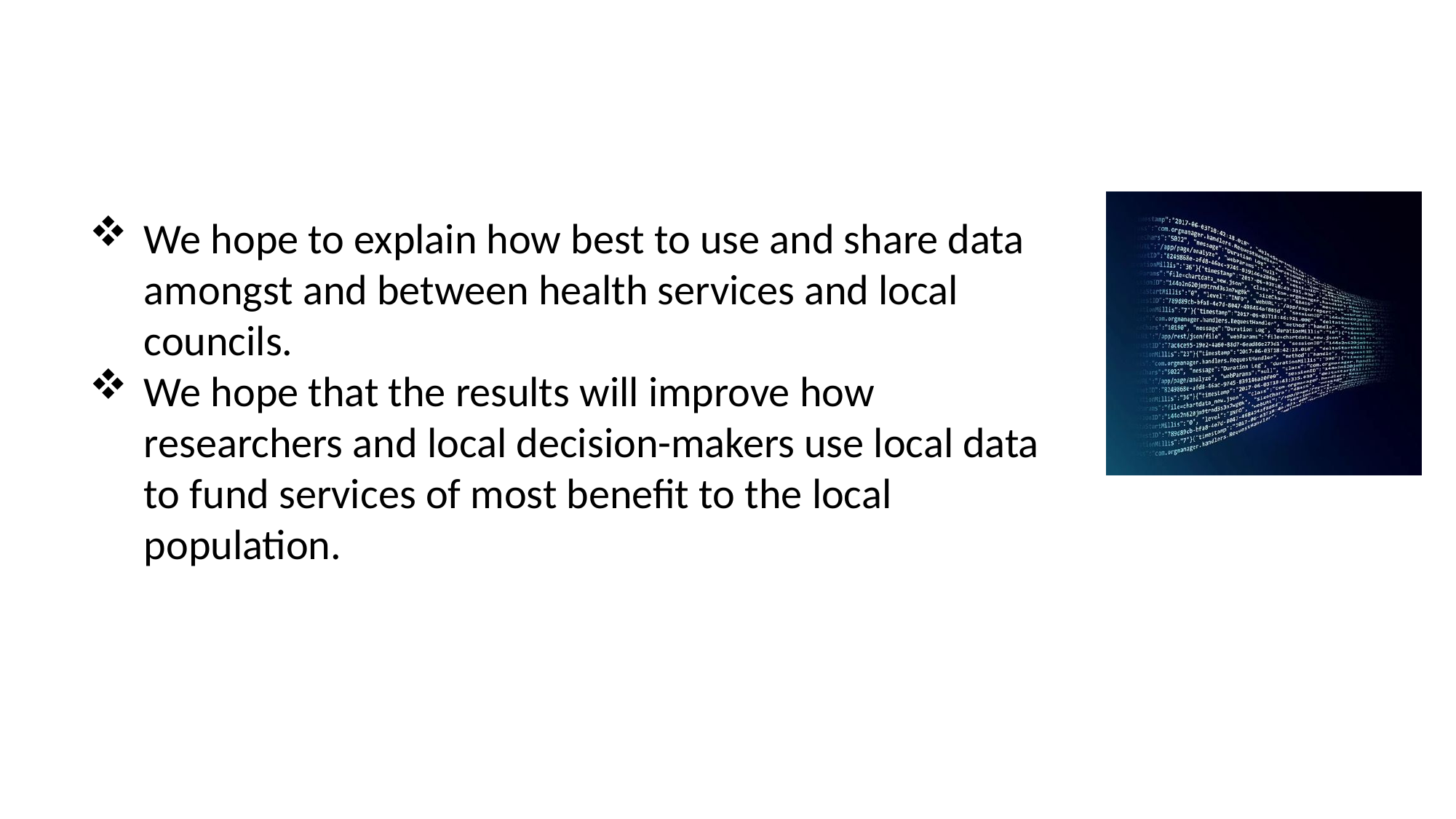

We hope to explain how best to use and share data amongst and between health services and local councils.
We hope that the results will improve how researchers and local decision-makers use local data to fund services of most benefit to the local population.

## Slide 62
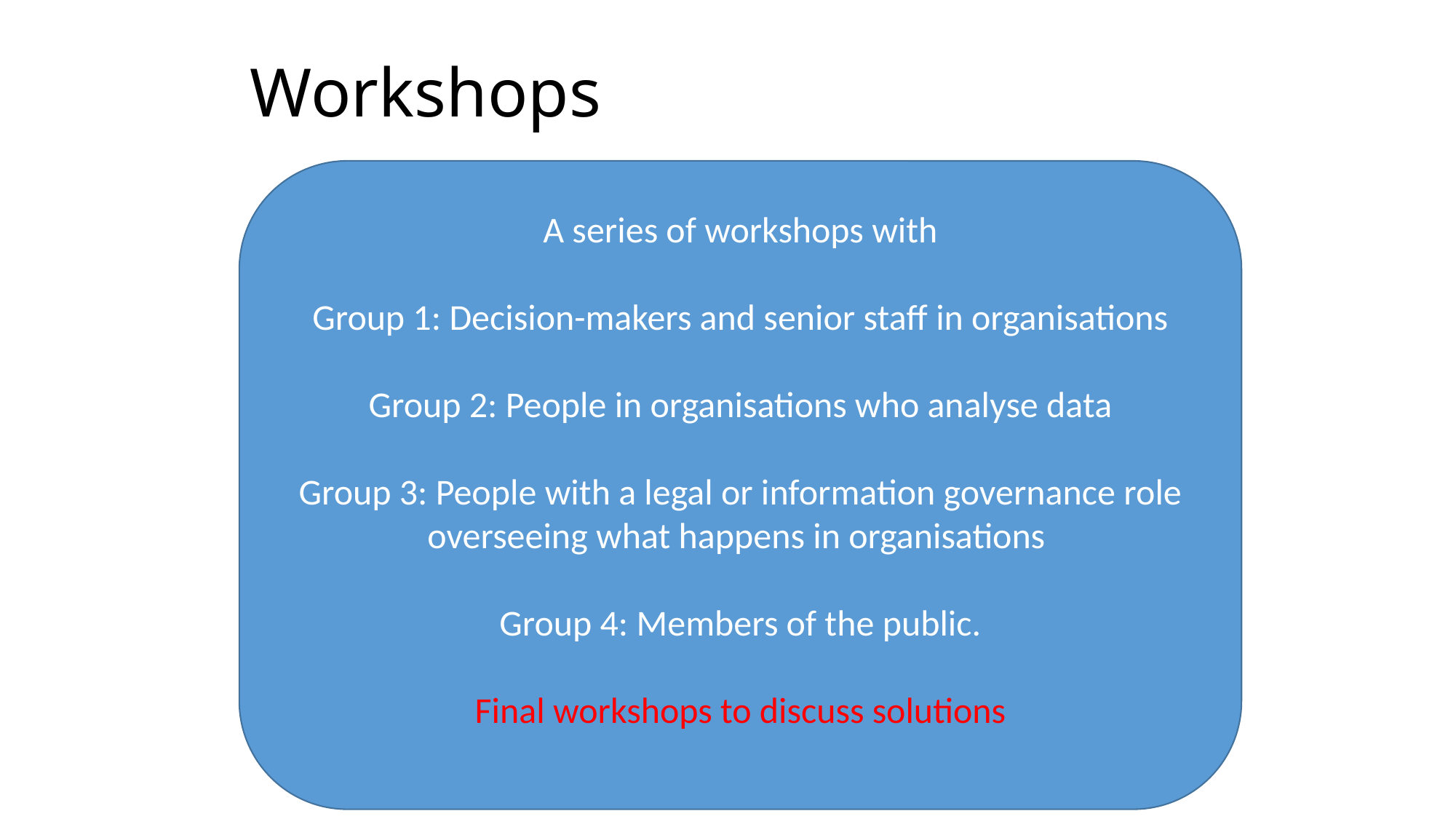

# Workshops
A series of workshops with
Group 1: Decision-makers and senior staff in organisations
Group 2: People in organisations who analyse data
Group 3: People with a legal or information governance role overseeing what happens in organisations
Group 4: Members of the public.
Final workshops to discuss solutions

## Slide 63
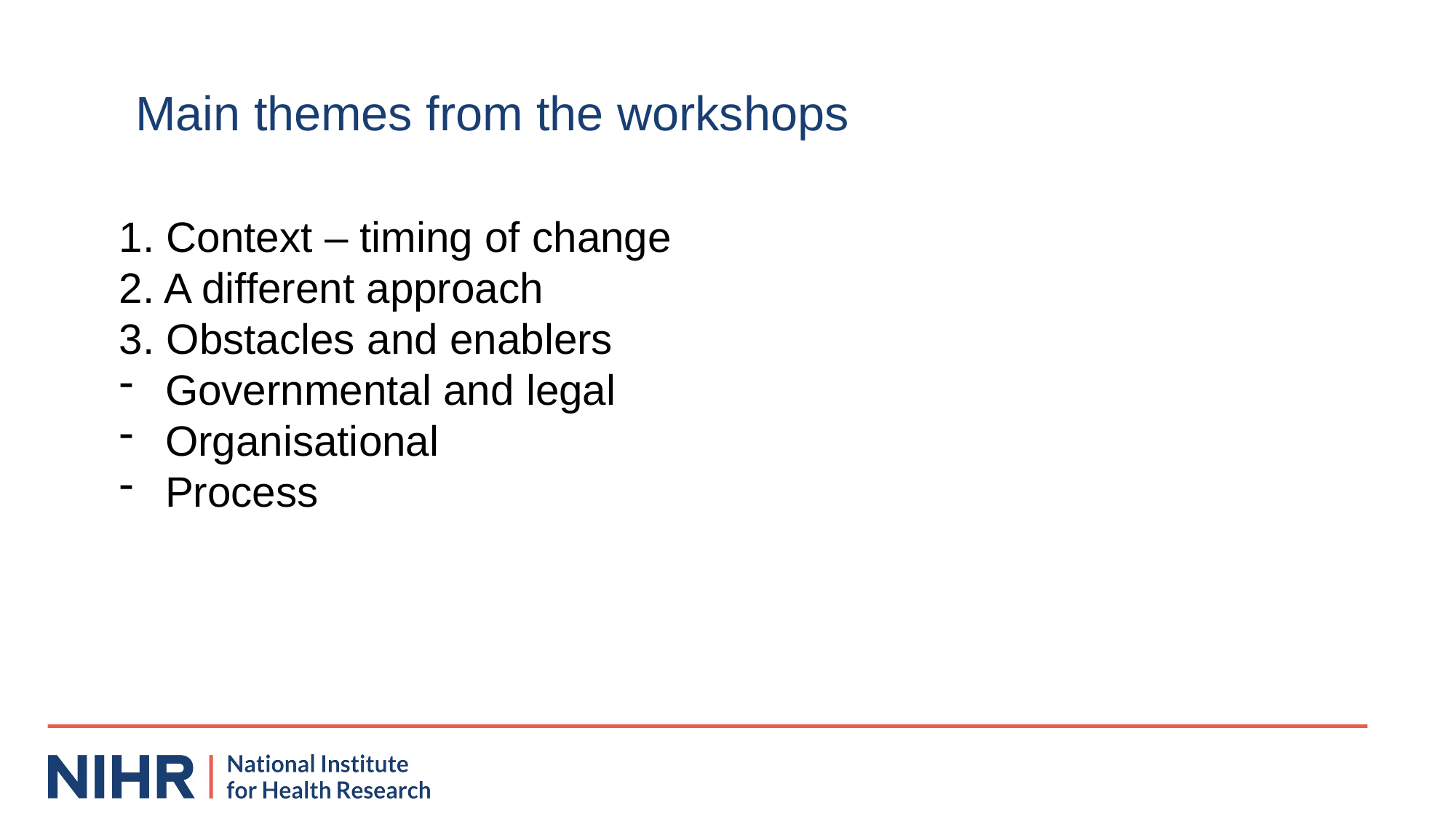

# Main themes from the workshops
1. Context – timing of change
2. A different approach
3. Obstacles and enablers
 Governmental and legal
 Organisational
 Process

## Slide 64
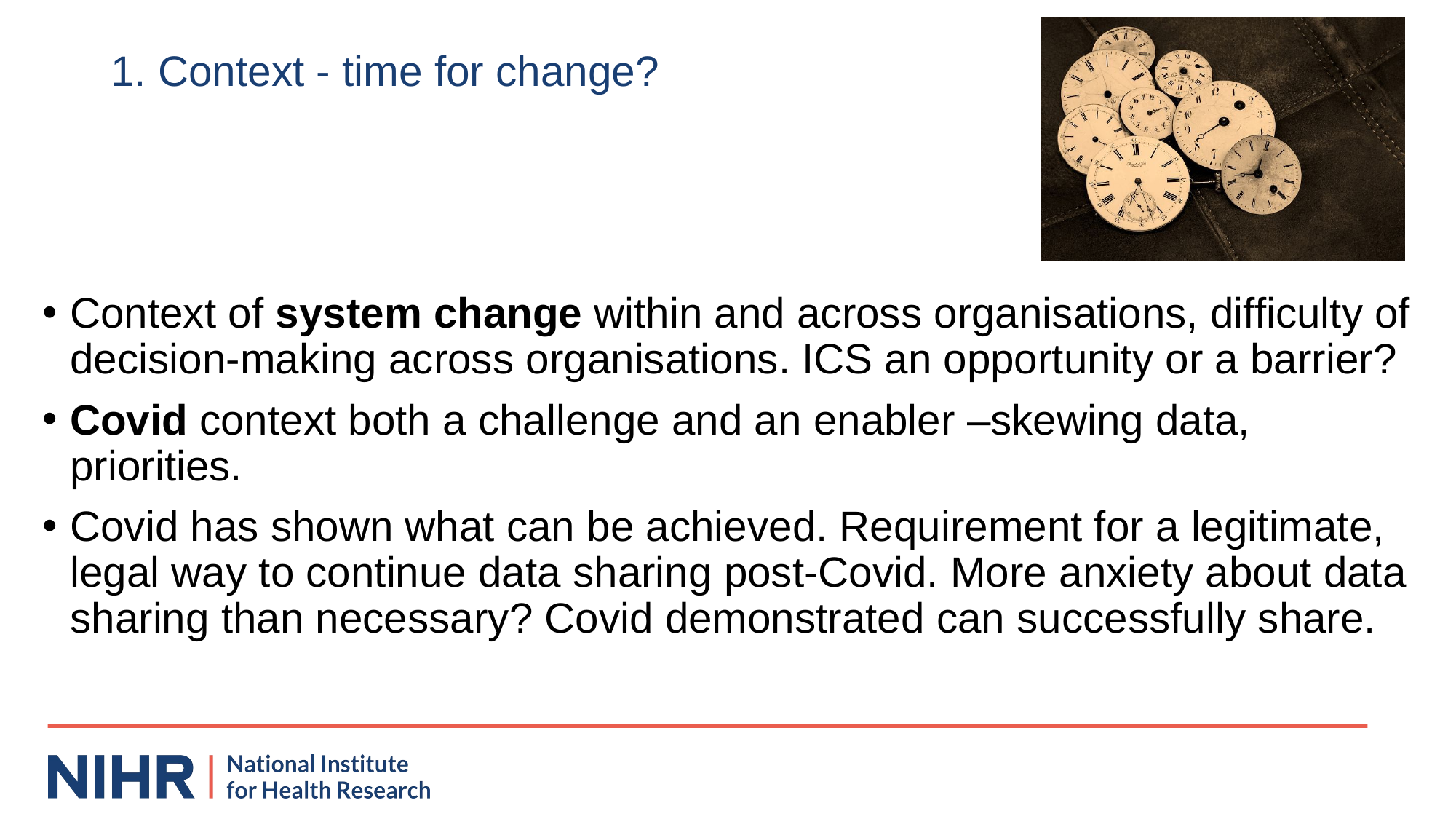

# 1. Context - time for change?
Context of system change within and across organisations, difficulty of decision-making across organisations. ICS an opportunity or a barrier?
Covid context both a challenge and an enabler –skewing data, priorities.
Covid has shown what can be achieved. Requirement for a legitimate, legal way to continue data sharing post-Covid. More anxiety about data sharing than necessary? Covid demonstrated can successfully share.

## Slide 65
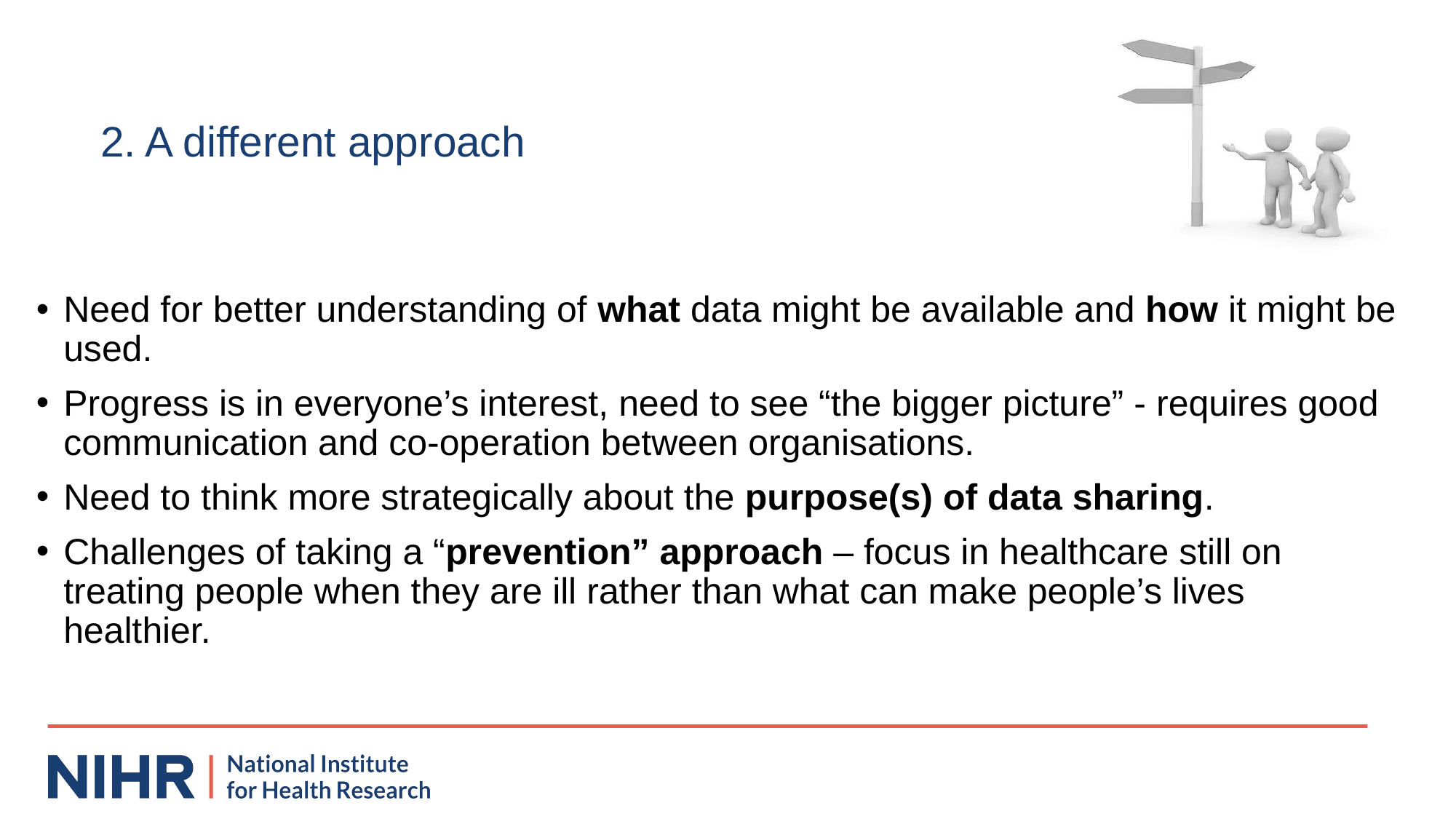

# 2. A different approach
Need for better understanding of what data might be available and how it might be used.
Progress is in everyone’s interest, need to see “the bigger picture” - requires good communication and co-operation between organisations.
Need to think more strategically about the purpose(s) of data sharing.
Challenges of taking a “prevention” approach – focus in healthcare still on treating people when they are ill rather than what can make people’s lives healthier.

## Slide 66
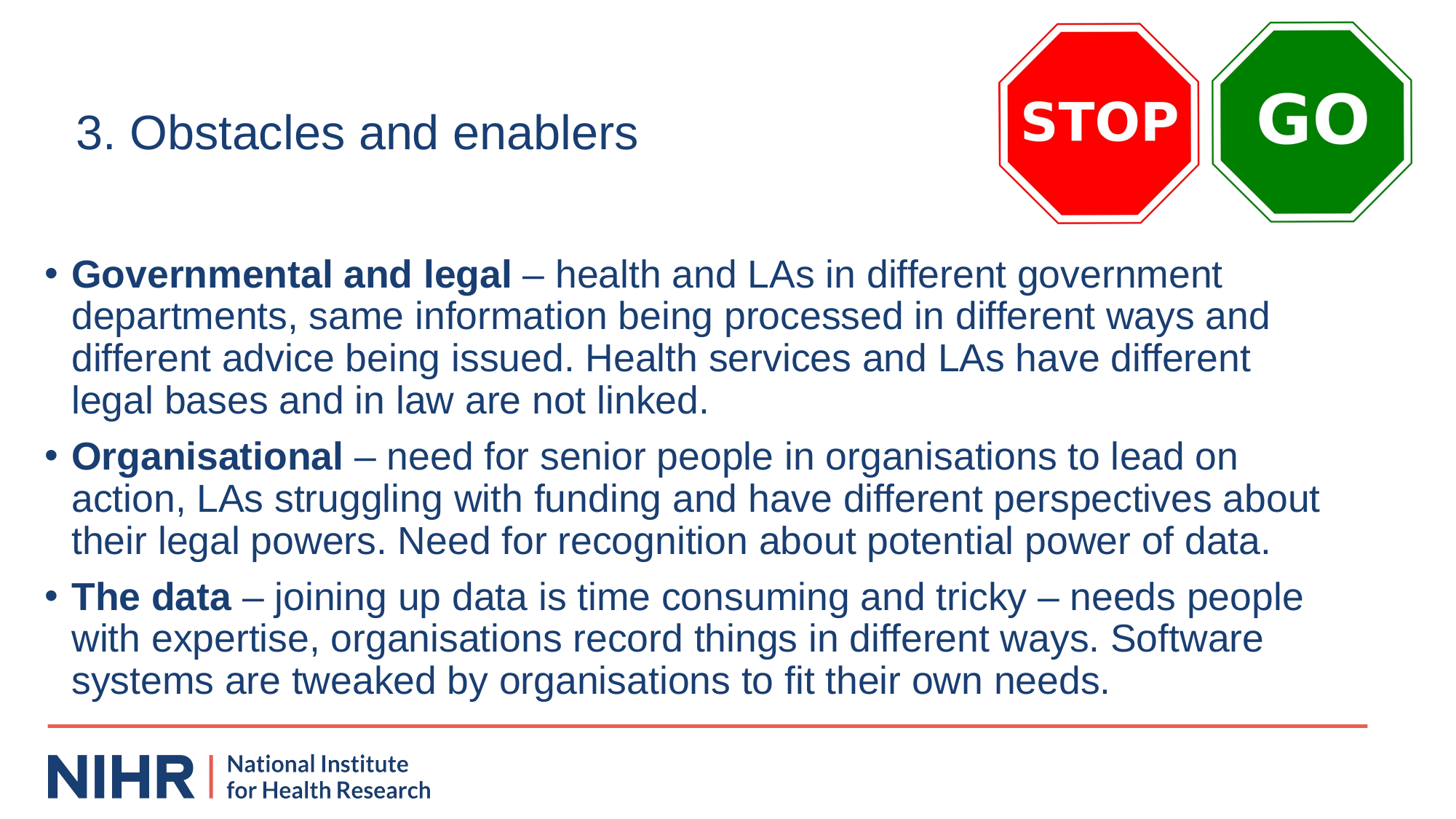

# 3. Obstacles and enablers
Governmental and legal – health and LAs in different government departments, same information being processed in different ways and different advice being issued. Health services and LAs have different legal bases and in law are not linked.
Organisational – need for senior people in organisations to lead on action, LAs struggling with funding and have different perspectives about their legal powers. Need for recognition about potential power of data.
The data – joining up data is time consuming and tricky – needs people with expertise, organisations record things in different ways. Software systems are tweaked by organisations to fit their own needs.

## Slide 67
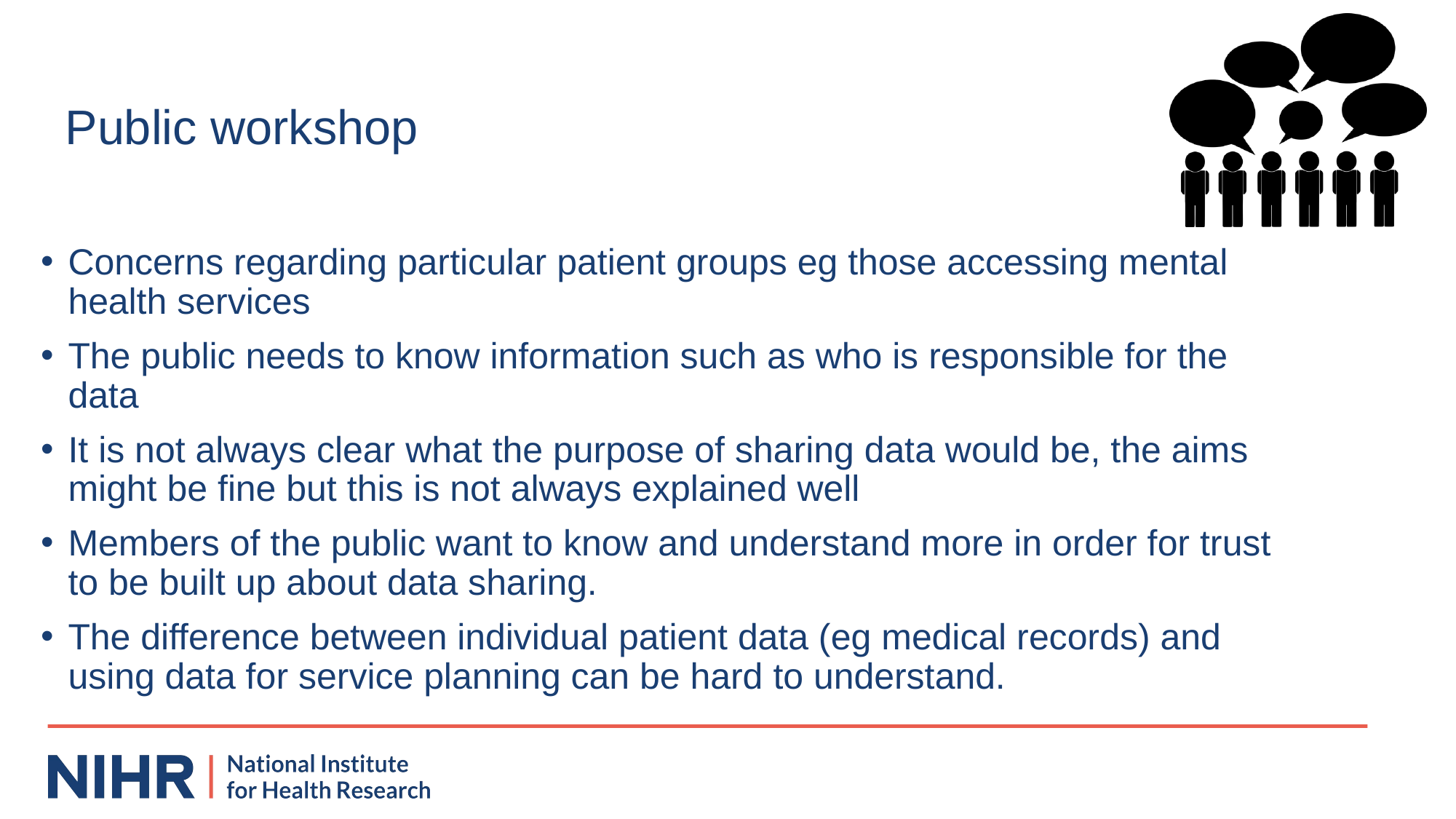

# Public workshop
Concerns regarding particular patient groups eg those accessing mental health services
The public needs to know information such as who is responsible for the data
It is not always clear what the purpose of sharing data would be, the aims might be fine but this is not always explained well
Members of the public want to know and understand more in order for trust to be built up about data sharing.
The difference between individual patient data (eg medical records) and using data for service planning can be hard to understand.

## Slide 68
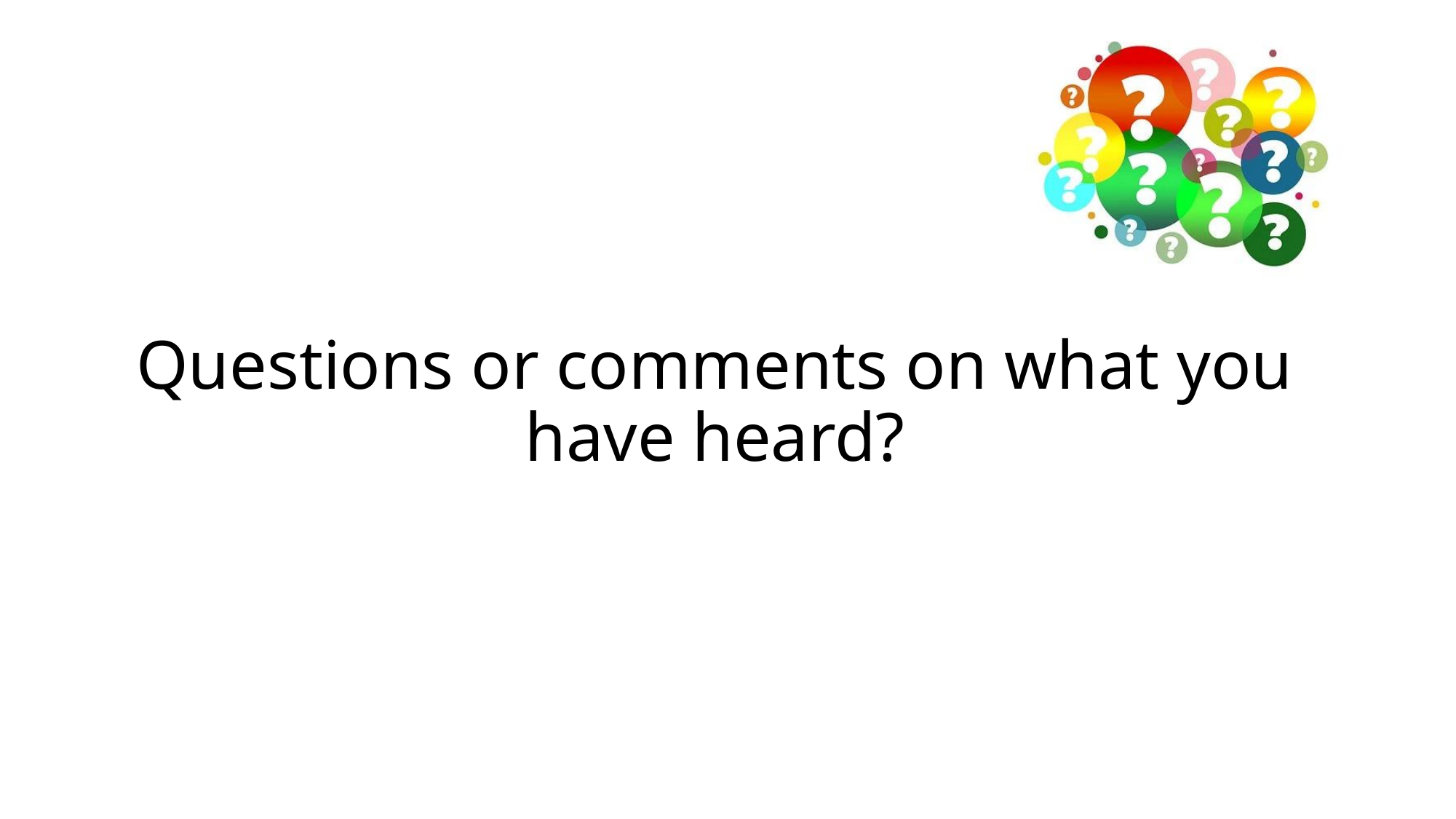

# Questions or comments on what you have heard?

## Slide 69
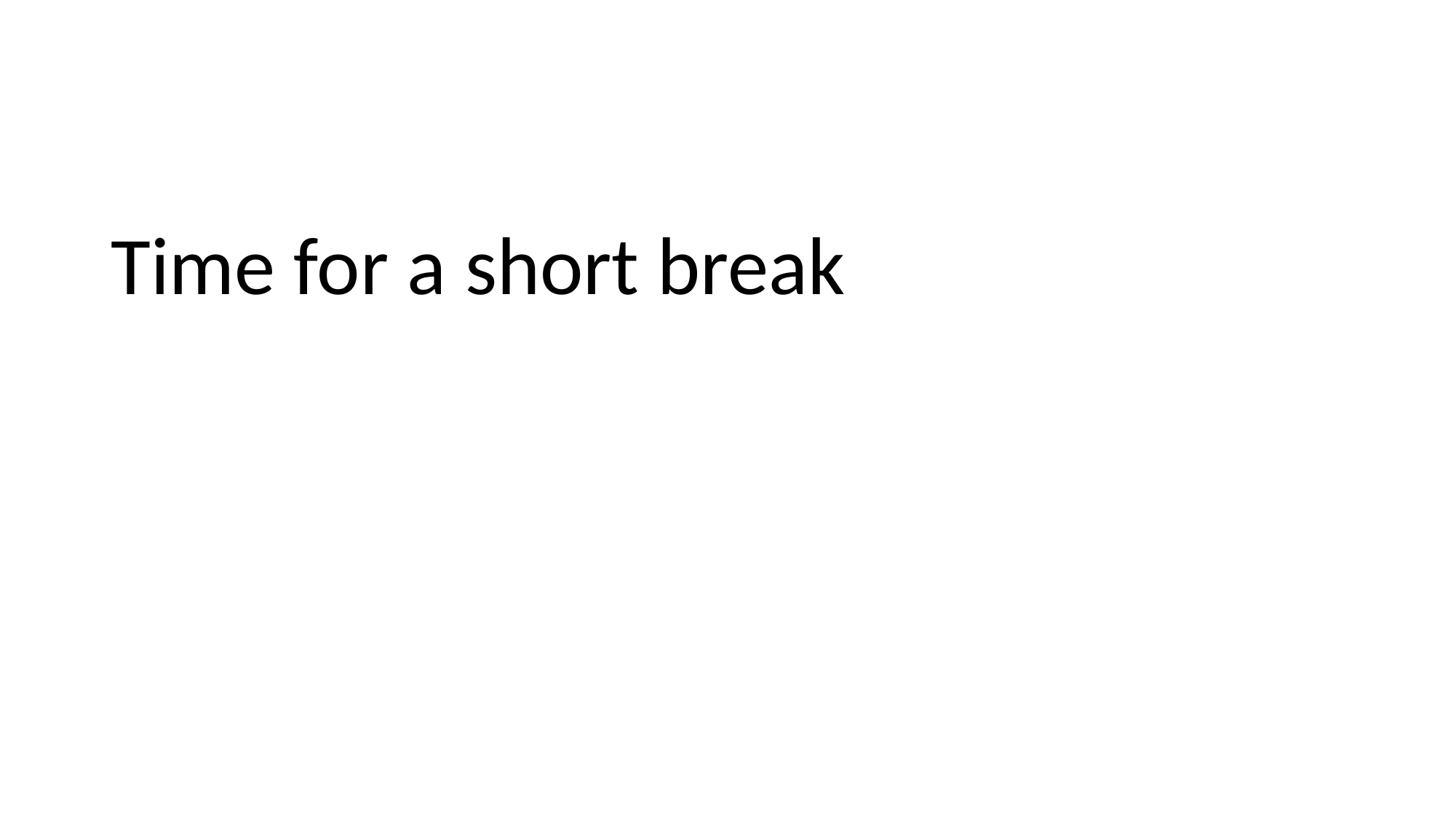

Time for a short break

## Slide 70
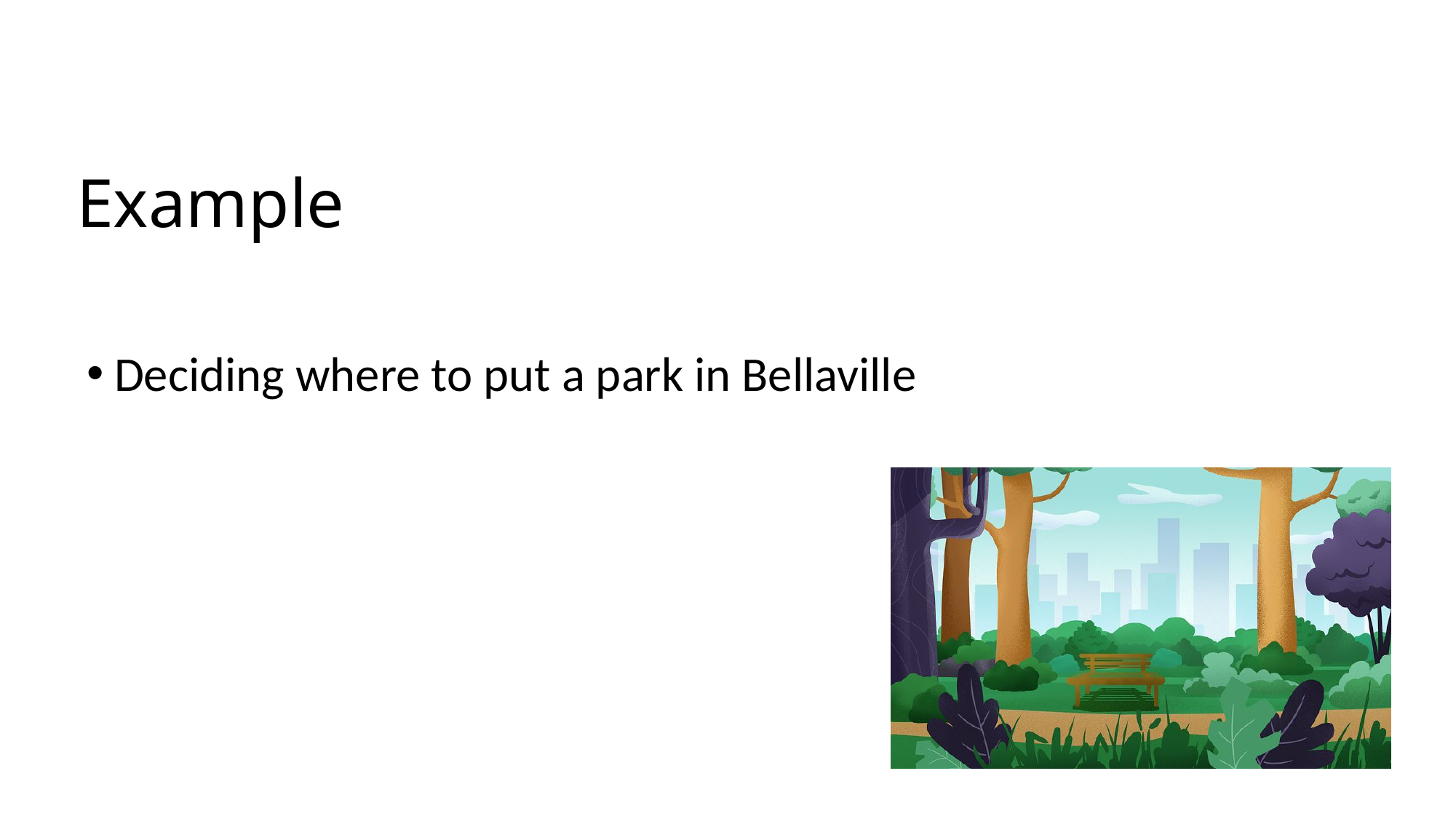

# Example
Deciding where to put a park in Bellaville

## Slide 71
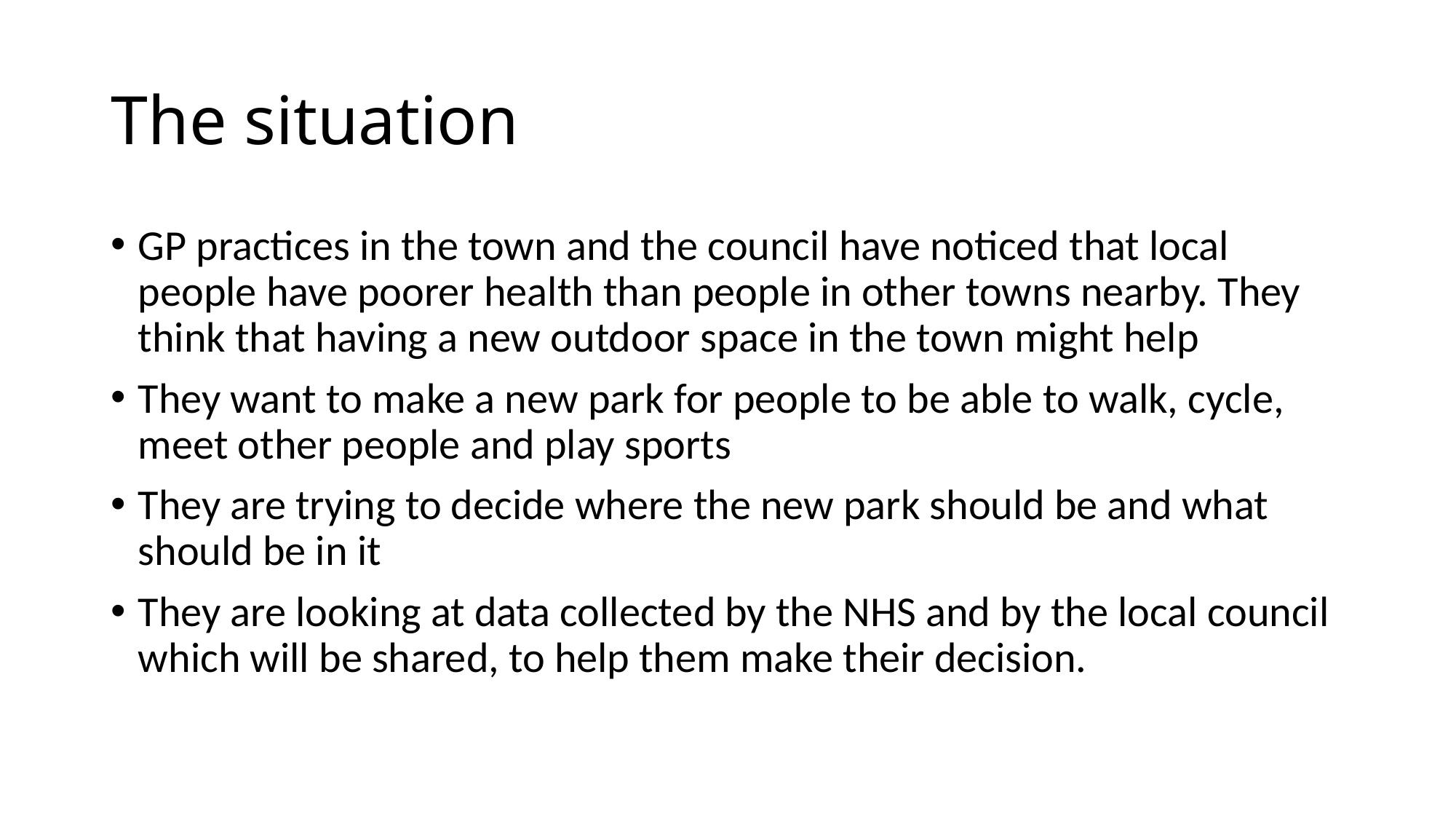

# The situation
GP practices in the town and the council have noticed that local people have poorer health than people in other towns nearby. They think that having a new outdoor space in the town might help
They want to make a new park for people to be able to walk, cycle, meet other people and play sports
They are trying to decide where the new park should be and what should be in it
They are looking at data collected by the NHS and by the local council which will be shared, to help them make their decision.

## Slide 72
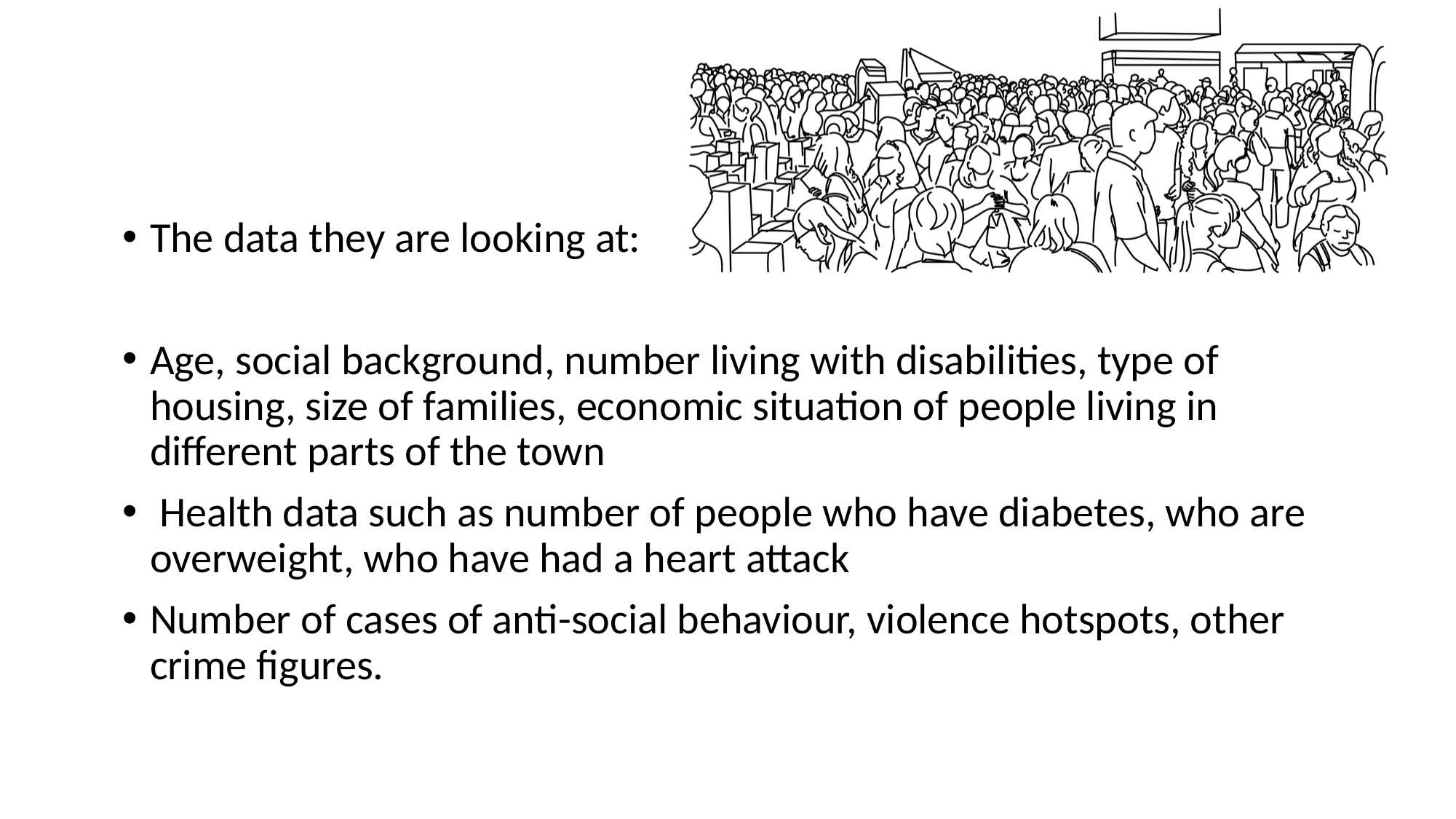

The data they are looking at:
Age, social background, number living with disabilities, type of housing, size of families, economic situation of people living in different parts of the town
 Health data such as number of people who have diabetes, who are overweight, who have had a heart attack
Number of cases of anti-social behaviour, violence hotspots, other crime figures.

## Slide 73
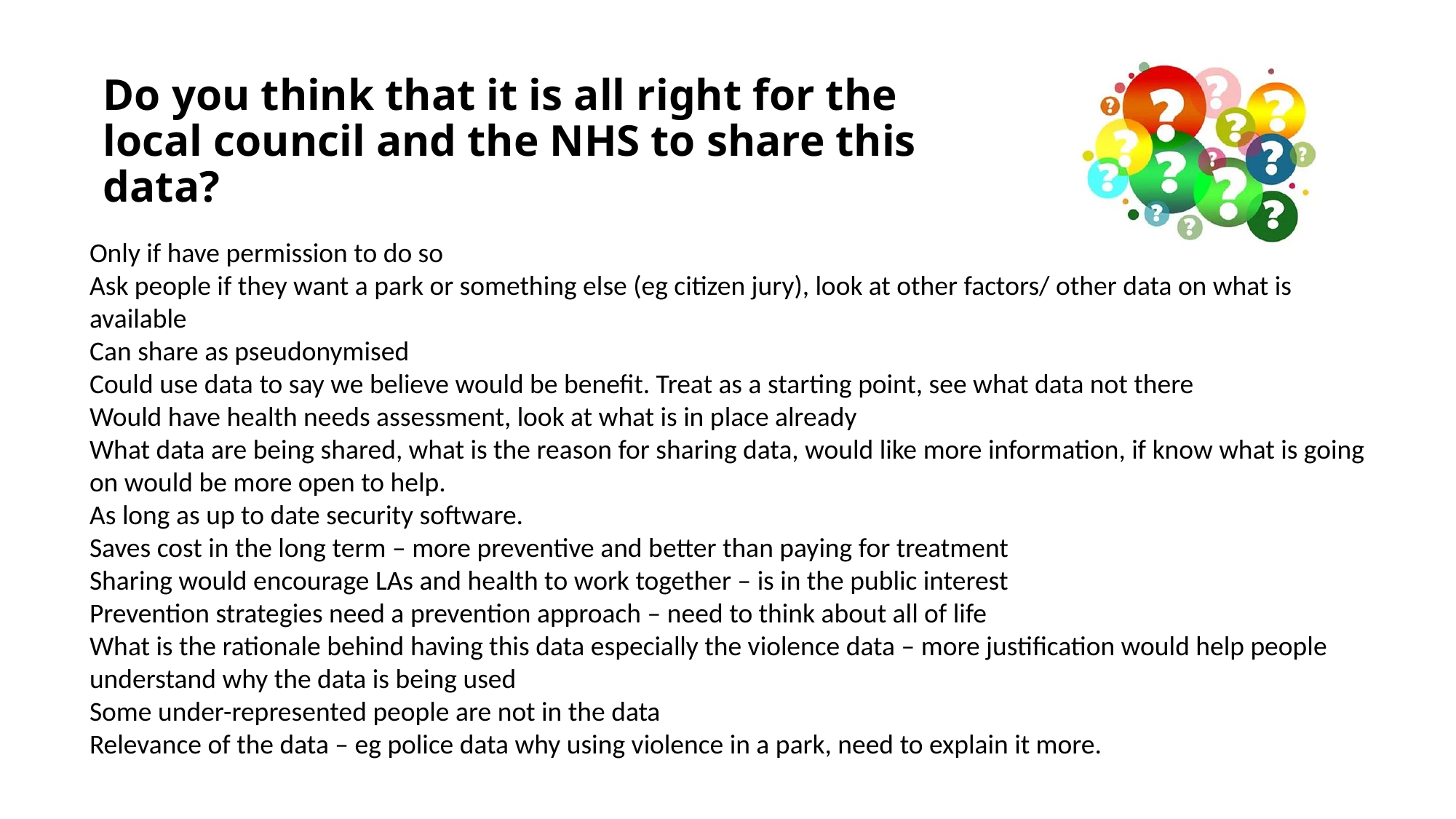

# Do you think that it is all right for the local council and the NHS to share this data?
Only if have permission to do so
Ask people if they want a park or something else (eg citizen jury), look at other factors/ other data on what is available
Can share as pseudonymised
Could use data to say we believe would be benefit. Treat as a starting point, see what data not there
Would have health needs assessment, look at what is in place already
What data are being shared, what is the reason for sharing data, would like more information, if know what is going on would be more open to help.
As long as up to date security software.
Saves cost in the long term – more preventive and better than paying for treatment
Sharing would encourage LAs and health to work together – is in the public interest
Prevention strategies need a prevention approach – need to think about all of life
What is the rationale behind having this data especially the violence data – more justification would help people understand why the data is being used
Some under-represented people are not in the data
Relevance of the data – eg police data why using violence in a park, need to explain it more.

## Slide 74
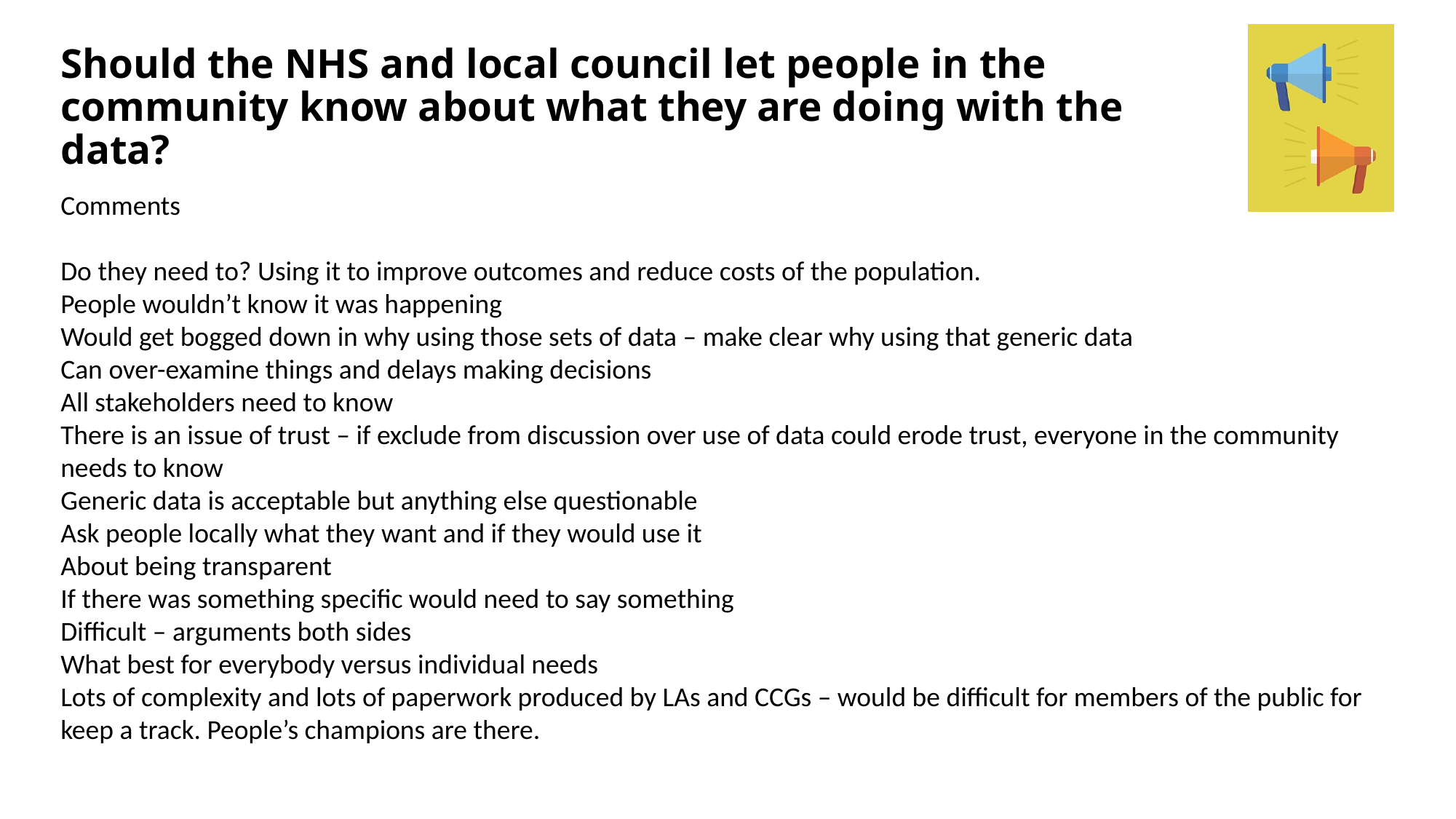

# Should the NHS and local council let people in the community know about what they are doing with the data?
Comments
Do they need to? Using it to improve outcomes and reduce costs of the population.
People wouldn’t know it was happening
Would get bogged down in why using those sets of data – make clear why using that generic data
Can over-examine things and delays making decisions
All stakeholders need to know
There is an issue of trust – if exclude from discussion over use of data could erode trust, everyone in the community needs to know
Generic data is acceptable but anything else questionable
Ask people locally what they want and if they would use it
About being transparent
If there was something specific would need to say something
Difficult – arguments both sides
What best for everybody versus individual needs
Lots of complexity and lots of paperwork produced by LAs and CCGs – would be difficult for members of the public for keep a track. People’s champions are there.

## Slide 75
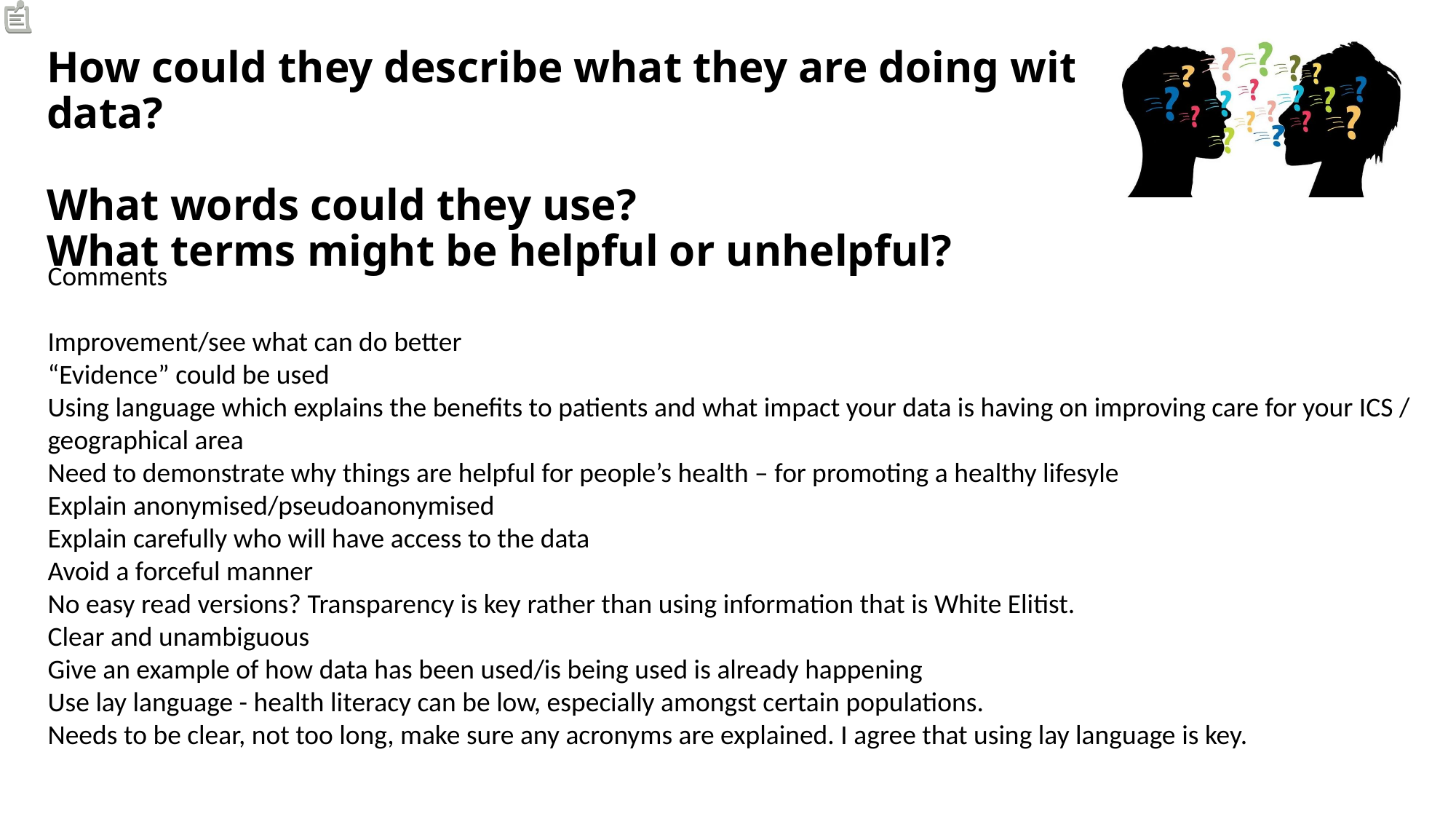

# How could they describe what they are doing with the data? What words could they use? What terms might be helpful or unhelpful?
Comments
Improvement/see what can do better
“Evidence” could be used
Using language which explains the benefits to patients and what impact your data is having on improving care for your ICS / geographical area
Need to demonstrate why things are helpful for people’s health – for promoting a healthy lifesyle
Explain anonymised/pseudoanonymised
Explain carefully who will have access to the data
Avoid a forceful manner
No easy read versions? Transparency is key rather than using information that is White Elitist.
Clear and unambiguous
Give an example of how data has been used/is being used is already happening
Use lay language - health literacy can be low, especially amongst certain populations.
Needs to be clear, not too long, make sure any acronyms are explained. I agree that using lay language is key.

## Slide 76
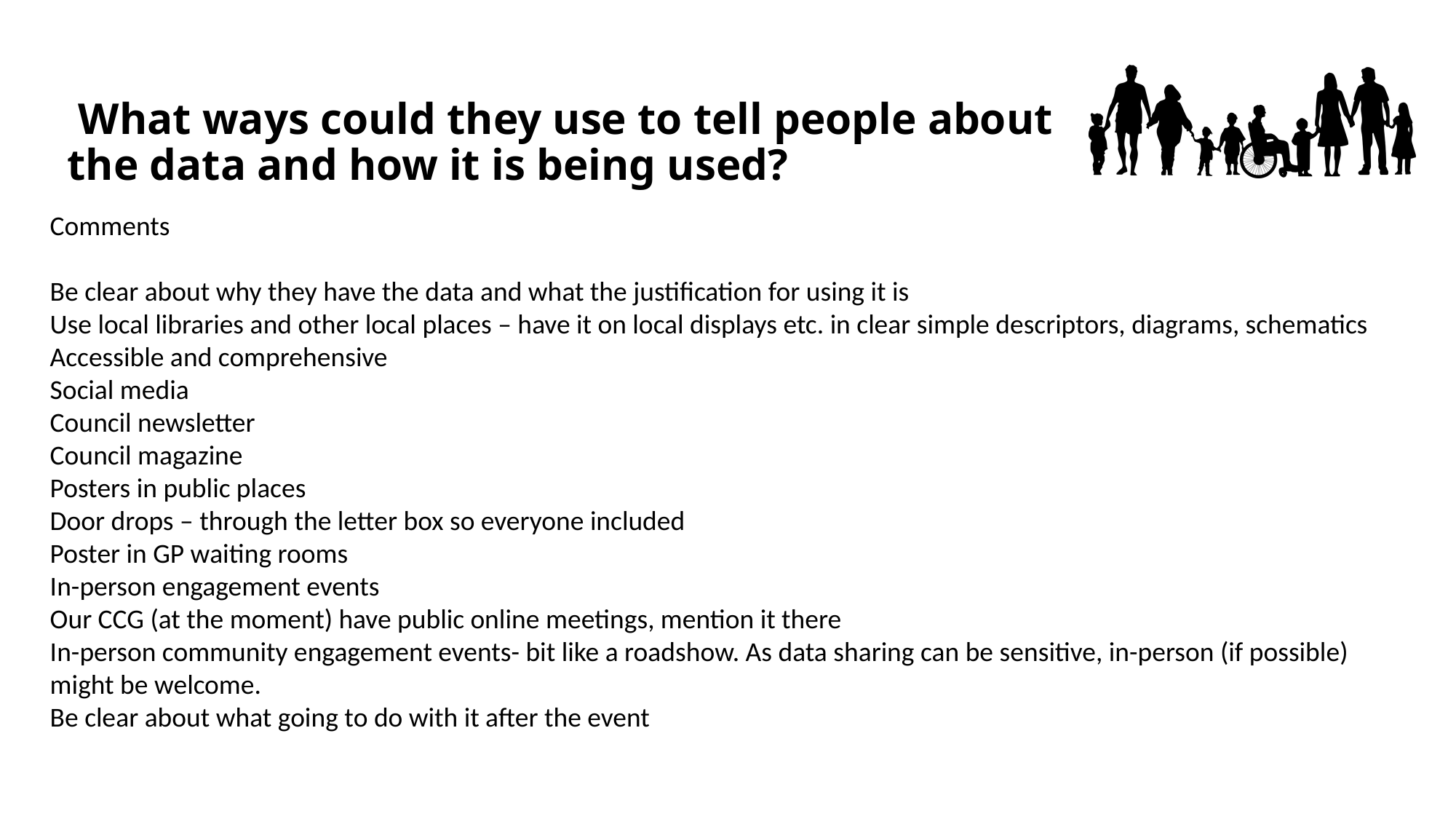

# What ways could they use to tell people about the data and how it is being used?
Comments
Be clear about why they have the data and what the justification for using it is
Use local libraries and other local places – have it on local displays etc. in clear simple descriptors, diagrams, schematics
Accessible and comprehensive
Social media
Council newsletter
Council magazine
Posters in public places
Door drops – through the letter box so everyone included
Poster in GP waiting rooms
In-person engagement events
Our CCG (at the moment) have public online meetings, mention it there
In-person community engagement events- bit like a roadshow. As data sharing can be sensitive, in-person (if possible) might be welcome.
Be clear about what going to do with it after the event

## Slide 77
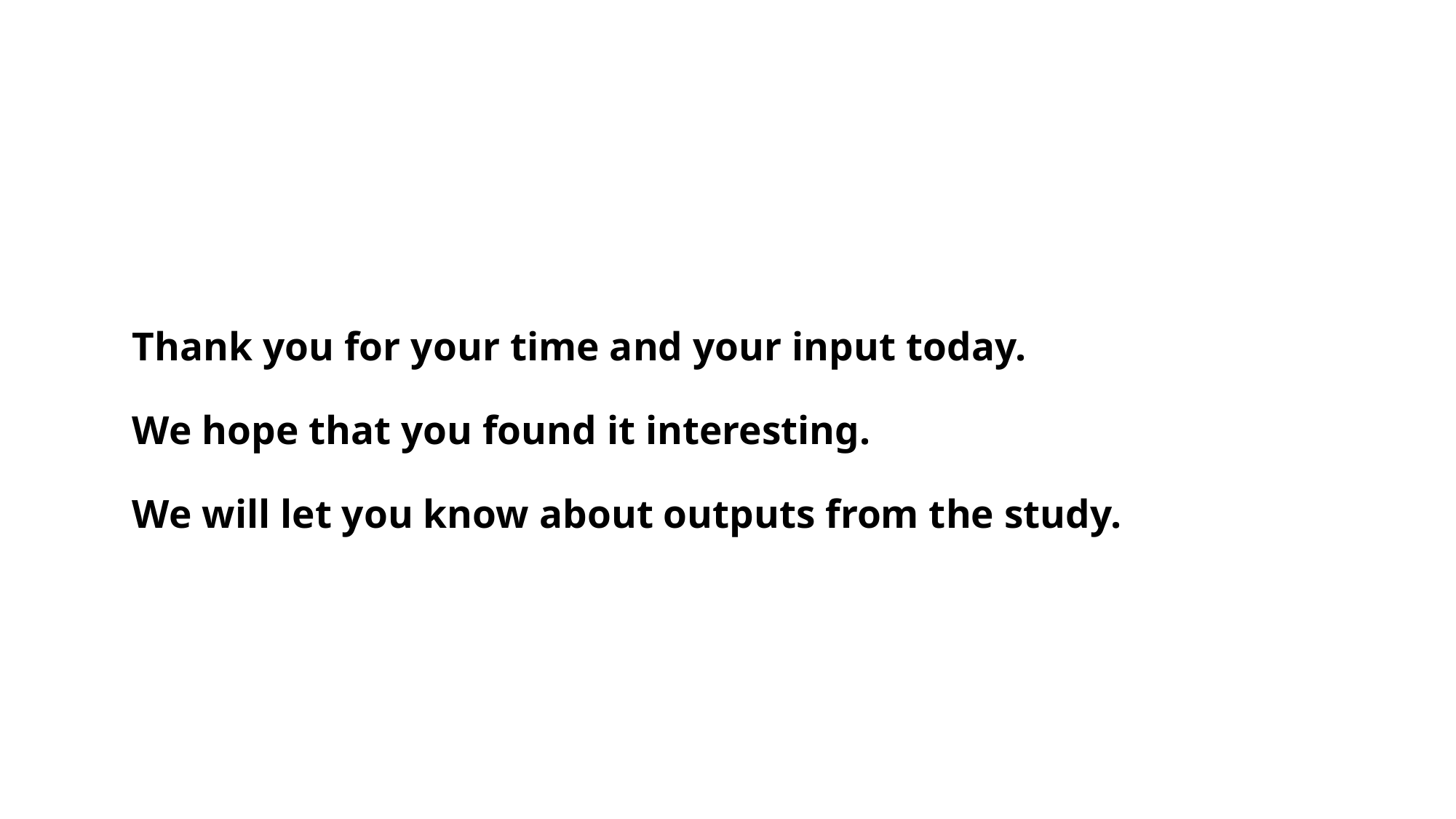

# Thank you for your time and your input today. We hope that you found it interesting. We will let you know about outputs from the study.
